# Cellular and molecular determinants of all-*trans* retinoic acid sensitivity in breast cancer: *Luminal* phenotype and RARα expression

**DOI:** 10.15252/emmm.201404670

**Published:** 2015-04-17

**Authors:** Floriana Centritto, Gabriela Paroni, Marco Bolis, Silvio Ken Garattini, Mami Kurosaki, Maria Monica Barzago, Adriana Zanetti, James Neil Fisher, Mark Francis Scott, Linda Pattini, Monica Lupi, Paolo Ubezio, Francesca Piccotti, Alberto Zambelli, Paola Rizzo, Maurizio Gianni', Maddalena Fratelli, Mineko Terao, Enrico Garattini

**Affiliations:** 1 Laboratory of Molecular Biology, IRCCS-Istituto di Ricerche Farmacologiche “Mario Negri”Milano, Italy; 2 Department of Electronics, Information and Bioengineering, Politecnico di MilanoMilan, Italy; 3 Department of Oncology, IRCCS-Istituto di Ricerche Farmacologiche “Mario Negri”Milano, Italy; 4 IRCCS-Fondazione “Salvatore Maugeri”Pavia, Italy; 5 Oncologia Medica, Ospedale Papa Giovanni XXIIIBergamo, Italy; 6 Gene Therapy and Cellular Reprogramming, IRCCS- Istituto di Ricerche Farmacologiche “Mario Negri”Bergamo, Italy

**Keywords:** breast cancer, luminal phenotype, nuclear receptor, RARalpha, retinoic acid

## Abstract

Forty-two cell lines recapitulating mammary carcinoma heterogeneity were profiled for all-*trans* retinoic acid (ATRA) sensitivity. *Luminal* and ER^+^ (estrogen-receptor-positive) cell lines are generally sensitive to ATRA, while refractoriness/low sensitivity is associated with a *Basal* phenotype and HER2 positivity. Indeed, only 2 *Basal* cell lines (*MDA-MB157* and *HCC-1599*) are highly sensitive to the retinoid. Sensitivity of *HCC-1599* cells is confirmed in xenotransplanted mice. Short-term tissue-slice cultures of surgical samples validate the cell-line results and support the concept that a high proportion of *Luminal/*ER^+^ carcinomas are ATRA sensitive, while triple-negative (*Basal*) and HER2-positive tumors tend to be retinoid resistant. Pathway-oriented analysis of the constitutive gene-expression profiles in the cell lines identifies RARα as the member of the retinoid pathway directly associated with a *Luminal* phenotype, estrogen positivity and ATRA sensitivity. RARα3 is the major transcript in ATRA-sensitive cells and tumors. Studies in selected cell lines with agonists/antagonists confirm that RARα is the principal mediator of ATRA responsiveness. RARα over-expression sensitizes retinoid-resistant *MDA-MB453* cells to ATRA anti-proliferative action. Conversely, silencing of RARα in retinoid-sensitive *SKBR3* cells abrogates ATRA responsiveness. All this is paralleled by similar effects on ATRA-dependent inhibition of cell motility, indicating that RARα may mediate also ATRA anti-metastatic effects. We define gene sets of predictive potential which are associated with ATRA sensitivity in breast cancer cell lines and validate them in short-term tissue cultures of *Luminal/*ER^+^ and triple-negative tumors. In these last models, we determine the perturbations in the transcriptomic profiles afforded by ATRA. The study provides fundamental information for the development of retinoid-based therapeutic strategies aimed at the stratified treatment of breast cancer subtypes.

## Introduction

ATRA (all-*trans* retinoic acid) is used in the management of acute promyelocytic leukemia (Tallman *et al*, 1997; Lo-Coco *et al*, 2013), and the retinoid holds promise for the treatment of solid tumors like breast cancer (Garattini *et al*, 2014). The mechanisms underlying ATRA anti-tumor activity are unique, as the compound is endowed with anti-proliferative and cyto-differentiating activities, while it is only a weak cytotoxic agent (Garattini *et al*, 2007a,b). The retinoid pathway centers on ligand-dependent transcription factors belonging to the family of steroid nuclear receptors along with ERs/PRs (estrogen/progesterone receptors) and PPARs (peroxisome proliferator-activated receptors) (Chambon, 1996; Mark *et al*, 2009). Six retinoid receptors are known, that is RARα/β/γ and RXRα/β/γ. Each RAR and RXR isoform is encoded by a distinct gene which is transcribed into splicing variants (Garattini *et al*, 2014). The active receptors consist of RXR-RAR heterodimers or RXR-RXR homodimers. While the RXR-RXR homodimers are the target of 9-*cis* retinoic acid and synthetic rexinoids, which are also promising agents in the chemoprevention of mammary tumors (Wu *et al*, 2002; Kong *et al*, 2005; Kim *et al*, 2006; Abba *et al*, 2008; Uray & Brown, 2011), the RXR-RAR heterodimers are the classic mediators of ATRA activity. In the RXR-RAR complexes, RARs act as the ligand-binding moiety. ATRA is a pan-RAR agonist, binding RARα/β/γ with the same affinity (Di Lorenzo *et al*, 1993; Gianni *et al*, 1996) and it also binds and activates PPARβ/δ (Shaw *et al*, 2003; Berry & Noy, 2007; Schug *et al*, 2007). In mammary tumors, ATRA-liganded RXR-RARs are purported to mediate growth inhibition, whereas RXR-PPARβ/δ induces proliferation (Noy, 2010). ATRA is transported to the nucleus by CRABP1 and CRABP2 (cytosolic retinoic acid-binding proteins-1/-2) as well as FABP5 (fatty acid-binding protein-5) (Schug *et al*, 2007). While CRABP2 delivers ATRA to RXR-RARs, FABP5 targets RXR-PPARβ/δ (Schug *et al*, 2007).

Breast cancer is a heterogeneous disease classified into subtypes according to the gene-expression profiles (Sorlie *et al*, 2003; Guedj *et al*, 2012). A rational use of ATRA and retinoids in breast cancer requires the definition of the sensitive subtypes (Garattini *et al*, 2014). Identification of the molecular determinants underlying retinoid sensitivity is another priority. The availability of representative cell lines characterized for the gene-expression profiles is a unique opportunity to establish the cellular/molecular determinants of retinoid sensitivity in mammary tumors. In this study, we tested the susceptibility of a large panel of breast cancer cell lines to ATRA, subsequently validating and extending the results in short-term tissue cultures of primary tumors. We identify RARα as the main retinoid receptor variant mediating the anti-tumor activity of the retinoid. In addition, we define gene sets, which are associated with ATRA sensitivity, and are of predictive potential. Finally, we determine the perturbations of the transcriptome afforded by ATRA in *LuminalA/B* and triple-negative (*TN*) tumors.

## Results

### Sensitivity of breast cancer cell lines to retinoids

**Figure 1:**
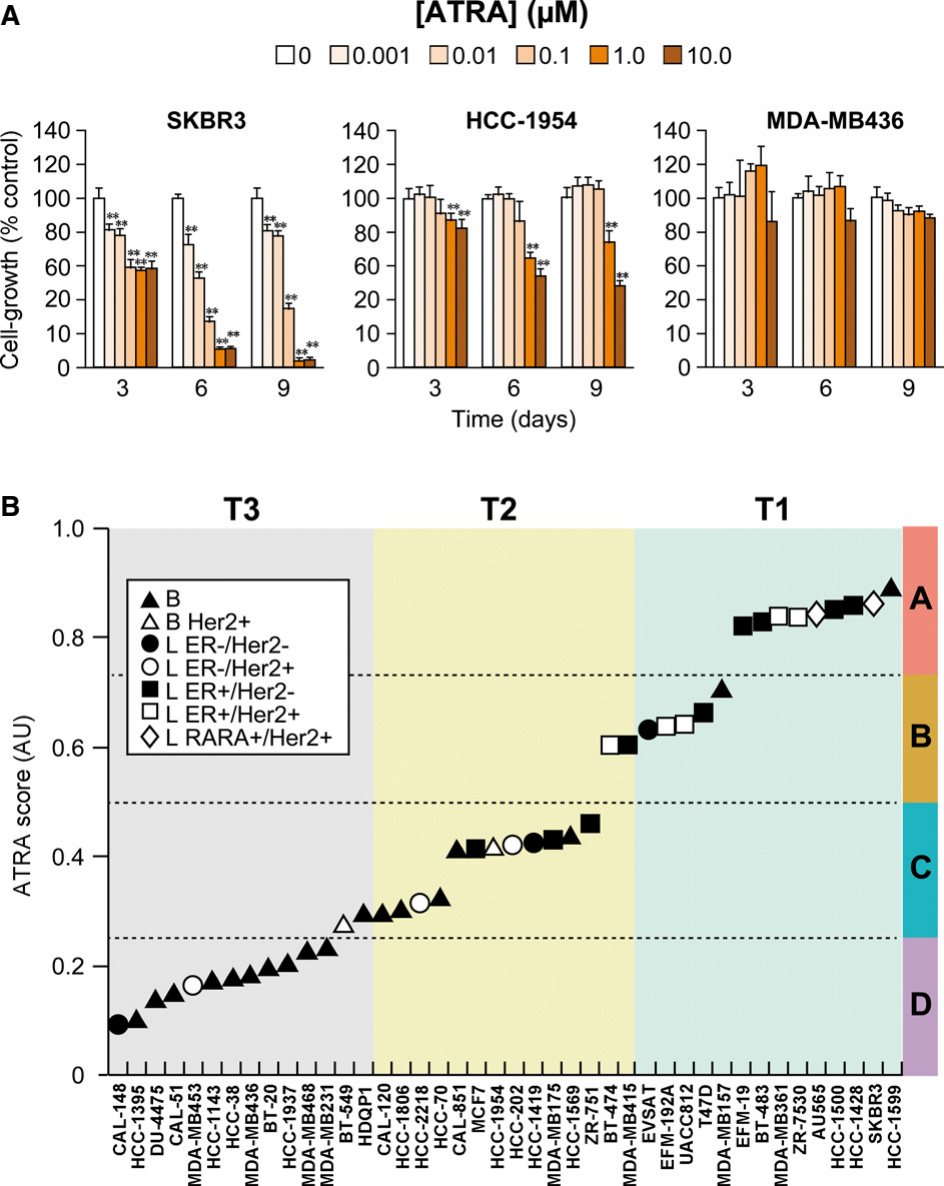
Profiling of the breast cancer cell-line panel according to ATRA sensitivity A panel of 42 breast cancer cell lines was challenged with increasing concentrations of ATRA (11 nM–10 μM) for 3, 6, and 9 days, and cell growth was determined.
The graphs show the growth-inhibitory effect exerted by the indicated concentrations of ATRA in *SKBR3*, *HCC-1954*, and *MDA-MB436* cells which are representative of lines characterized by a high, intermediate and low *ATRA score*, respectively. Each point is the mean ± SD of three replicate cultures. **Significantly lower than the corresponding vehicle-treated group (*P*-value < 0.01, Student's *t*-test).

To define ATRA sensitivity, we selected 42 cell lines representative of breast cancer heterogeneity (Supplementary Table S1) and characterized for ER, PR, and HER2 status as well as the *Luminal* or *Basal* phenotype according to PAM50 (Tibshirani *et al*, 2002; Parker *et al*, 2009) (Supplementary Fig S1). The concentration-dependent growth-inhibitory effects of ATRA (0.001–10 μM) at 3, 6, and 9 days were evaluated, as exemplified by the *SKBR3*, *HCC-1954*, and *MDA-MB436* cell lines showing different ATRA sensitivity (Fig1A). The doubling time of each cell line and a number of other parameters associated with ATRA-dependent growth inhibition were determined (Supplementary Table S2). All these parameters are the basis for the calculation of the *ATRA score*, a new and robust index defining cell sensitivity to the growth-inhibitory action of ATRA (Supplementary Methods). The higher the *ATRA score* is, the higher is ATRA sensitivity. Development of this new index was necessary, since determination of standard IC_50_ values for the definition of sensitivity to the anti-proliferative effect of ATRA was deemed inadequate for at least two reasons. The IC_50_ is routinely and successfully used to assess cell sensitivity to cytotoxic compounds, while ATRA is predominantly a growth inhibitory and cyto-differentiating agent and it is largely devoid of a direct cytotoxic action (Garattini *et al*, 2007b, 2014). Given the slow kinetics of the response to the retinoid, we calculated the *ATRA score* between days 3 and 6.

Cell lines are ranked in ascending order according to the *ATRA score*. The plot distinguishes four separate groups of cell lines (D–A) with increasing *ATRA scores*, as indicated by the colored scale on the right. The cell lines are also grouped in tertiles, T1–T3, according to an ascending *ATRA score*. Each calculated value is representative of at least two independent experiments.

The *ATRA score* provides a continuous series of values across our panel of cell lines and identifies four separable groups (A–D, Fig1B). The subsets with high and intermediate sensitivity (groups A and B) are enriched for cells with *Luminal* and ER^+^ phenotypes. Indeed, 14/16 of the cell lines in combined groups A and B are *Luminal* and 11/16 are ER^+^. Interestingly, *SKBR3* and *AU565*, representing a subgroup of HER2^+^ tumors which is predicted to be sensitive to ATRA due to co-amplification of the *RARA* and *ERBB2* loci (Paroni *et al*, 2012), are the only ER^−^/HER2^+^ cell lines present in group A. Similarly, *HCC-1599* and *MDA-MB157* are the only *Basal* cell lines in groups A and B, respectively. Group C clusters the cell lines characterized by low sensitivity to ATRA. In this group, the proportion of *Luminal* (6/14) and ER^+^ (3/14) cell lines is reduced. Group D concentrates ATRA-resistant lines, the majority of which is *Basal* (10/12). Thus, the *ATRA scores* indicate that a *Luminal* phenotype and ER expression are major determinants of cell sensitivity to the anti-proliferative action of ATRA. In contrast, a *Basal* phenotype represents a negative factor. Indeed, the proportion of *Basal* cell lines increases as the *ATRA score* decreases if our panel is divided in tertiles (T1 = 2/14; T2 = 6/14; T3 = 12/14) (Fig1B).

**Figure 2:**
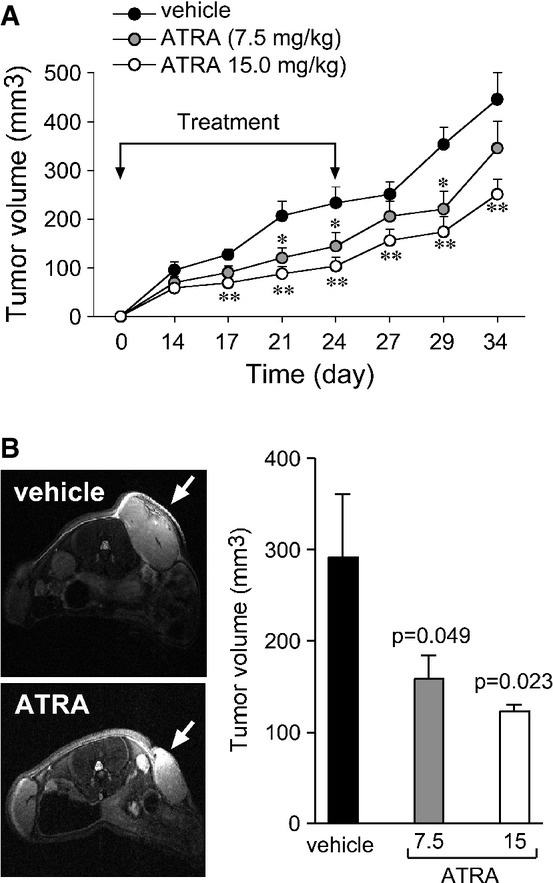
ATRA-dependent anti-tumor activity in *HCC*-*1599*-derived xenografts *in vivo* SCID mice were xenografted subcutaneously with 10 × 10^6^ *HCC-1599* cells on both sides. One week after transplantation 10 animals/experimental group were treated intraperitoneally with vehicle (DMSO) or two doses of ATRA (7.5 and 15.0 mg/kg) once/day, 5 days a week for a total of 24 days. At the end of this period, treatment was discontinued until sacrifice.
The size of the tumors was determined with a caliper and the volume plotted. Each point is the mean ± SE of 20 tumors. *Significantly lower than the corresponding vehicle-treated group (*P*-value < 0.05, Student's *t*-test). **Significantly lower than the corresponding vehicle-treated group (*P*-value < 0.01, Student's *t*-test).

Being one of the two *Basal* lines with a high *ATRA score* and one of the rare breast cancer lines transplantable in mice (Zhang *et al*, 2013), *HCC-1599* represents a unique model to validate our ATRA-sensitivity data *in vivo*. Thus, SCID mice bearing subcutaneous *HCC-1599* xenografts were treated with ATRA (15 and 7.5 mg/kg) or vehicle on a daily basis for 3 weeks, and tumor growth was followed. A time- and dose-dependent reduction in the tumor volume is evident in mice treated with ATRA (Fig2A). With the highest dose of ATRA, the effect is already significant after 17 days and is maintained for at least 10 days after treatment discontinuation. The total body weight of mice is not different in the experimental groups, demonstrating lack of ATRA-dependent toxicity (Supplementary Fig S2). The results were validated by MRI analyses performed at 24 days (Fig2B). Taken together, the results support the *in vivo* relevance of the cell-line studies.

Magnetic resonance imaging (MRI) analysis was performed on five animals/experimental group on day 24. The picture shows representative 2D images of tumor sections from one animal treated with vehicle and one animal treated with 15.0 mg/kg ATRA. The bar graph shows the volume of the tumors calculated after 3D reconstruction of the MRI images. Each point is the mean ± SE of five tumors. The *P*-value of the comparisons of ATRA versus vehicle is shown.

### Short-term cultures of mammary tumors: anti-proliferative responses to ATRA

**Figure 3:**
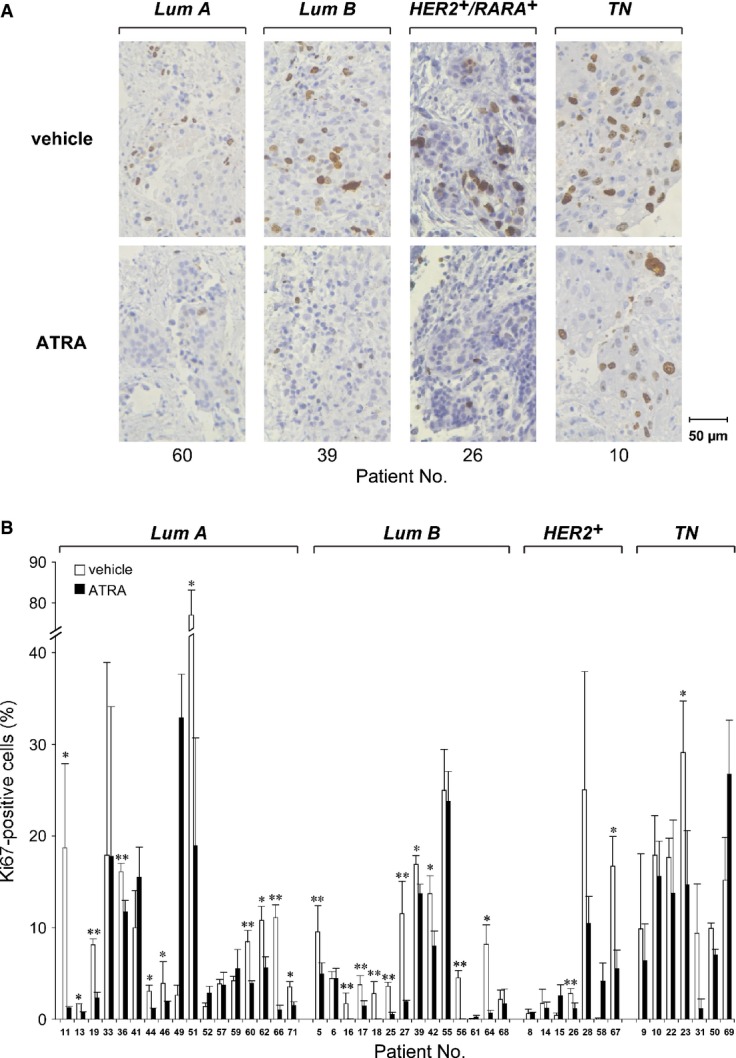
ATRA-dependent anti-tumor activity in short-term cultures of primary breast tumors Tissue slices deriving from surgical specimens were challenged with vehicle (DMSO) or ATRA (0.1 μM) for 48 h.
The panel illustrates examples of the immuno-histochemical data obtained in four representative cases: (i) *Luminal-A* (Lum A); (ii) *Luminal-B* (Lum B); (iii) *Her2*^+^ with RARA coamplification (*Her2*^+^/*RARA*^+^) and (iv) triple negative (*TN*).

To confirm the results obtained with the cell lines, we used short-term cultures of mammary tumors (van der Kuip *et al*, 2006) derived from diagnostic *Tru-cut* procedures of 45 patients (Supplementary Table S3). To assess the anti-proliferative activity of ATRA, tissue slices were challenged with vehicle or the retinoid for 48 h, the maximal time interval maintaining tumor cell viability in basal culture conditions. The growth of tumor cells was evaluated with Ki67 (Fig3A and B), which is an established biomarker of cell division and it is routinely used in the clinics to assess the proliferation rate of breast cancer. Ki67 is rapidly down-regulated by a number of anti-proliferative agents in short-term tissue cultures of primary tumors (Alagesan *et al*, 2015). Rapid down-regulation of the biomarker is of the outmost importance, given the relatively short exposure times to ATRA that our tissue culture model allows and the slow anti-proliferative effect exerted by the retinoid.

The percentage of Ki67-positive tumor cells in the 45 samples considered are illustrated by the bar graphs. Each value represents the mean ± SE of at least five separate fields for each experimental sample. *Significantly lower than the corresponding vehicle-treated control (*P*-value < 0.05, Student's *t*-test). **Significantly lower than the corresponding vehicle-treated control (*P*-value < 0.01, Student's *t*-test).

The cases used in our study are classified according to standard clinical criteria and consist of 17 *Luminal-A*, 14 *Luminal-B*, 7 HER2^+^, and 7 *TN* (triple-negative) tumors. All the *Luminal-A* and *Luminal-B* tumors are characterized by > 70% ER^+^ cells. ATRA reduces the proliferation of 11 *Luminal-A* and 10 *Luminal-B* tumors (Fig3B). Except for growth inhibition of the two cases characterized by co-amplification of the *ERBB2* and *RARA* loci (patients 26 and 67) (Paroni *et al*, 2012), the retinoid exerts no significant effect on HER2^+^ tumors. Only one of the *TN* or *Basal* cancers responds to ATRA. The data are consistent with the cell-line results and confirm that ATRA sensitivity is frequent in *Luminal* and ER^+^ tumors.

### Associations between the cellular phenotype and genes of the retinoid pathway

**Figure 4:**
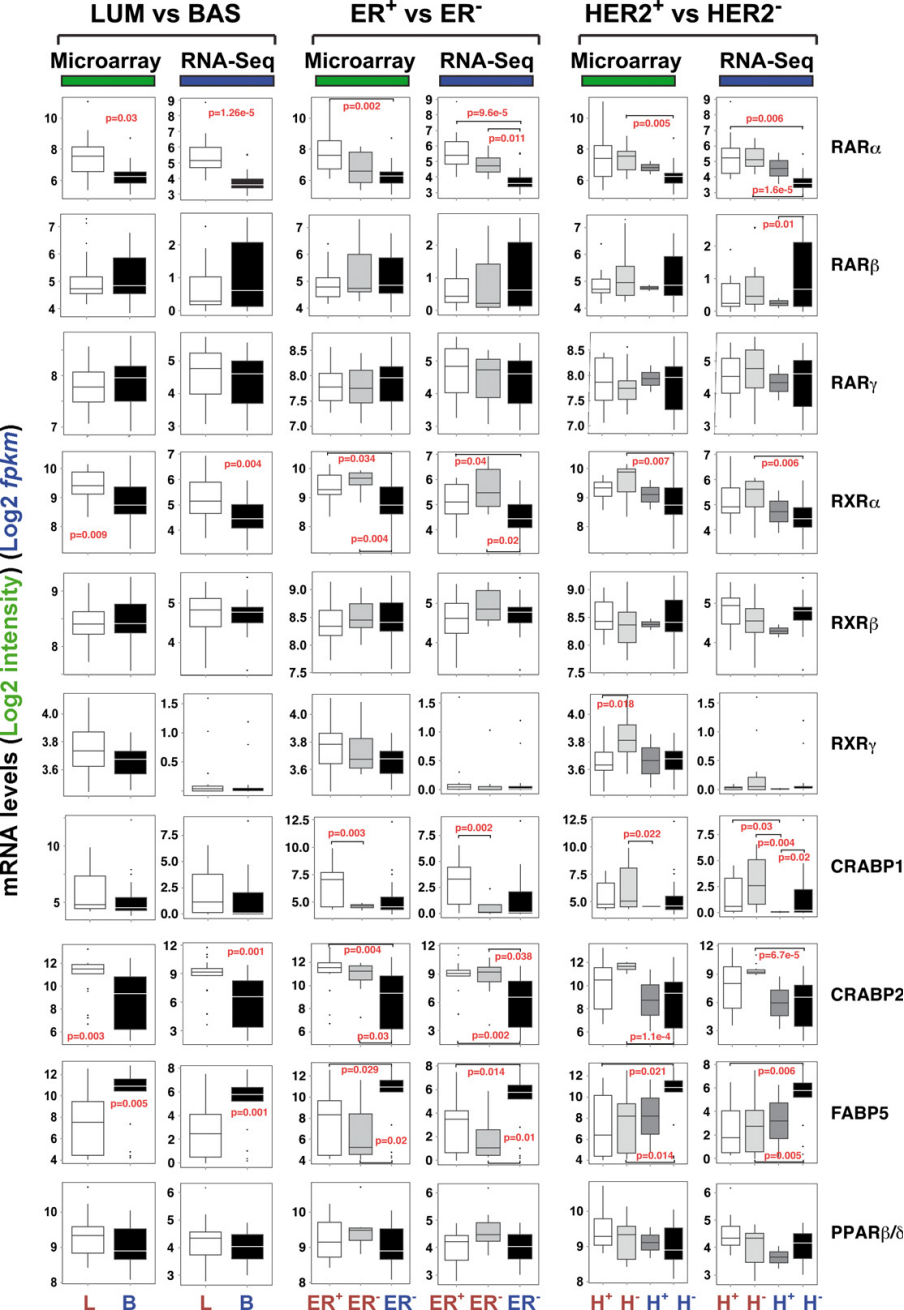
Associations between components of the retinoid signaling pathway and the phenotype in breast cancer cell lines The figure illustrates the associations between the indicated retinoid receptors/binding proteins and the *Luminal* versus *Basal* phenotype (left panels), ER positivity versus ER negativity (middle panels) as well as HER2 positivity versus HER2 negativity (right panels). The gene-expression microarray and RNA-seq data refer to 42 and 40 breast cancer cell lines, respectively. The *P*-values of the indicated comparisons after Student's *t*-test are shown in red. L (red) = *Luminal* cell lines; B (blue) = *Basal* cell lines; ER^+^ (red) = ER-positive cell lines; ER^−^ (red) = ER-negative cell lines; ER^−^ (blue) = ER-negative *Luminal* cell lines; H^+^ (red) = HER2-positive cell lines; H^−^ (red) = HER2-negative cell lines; H^+^ (blue) = HER2-positive *Luminal* cell lines; H^−^ (blue) = HER2-negative *Luminal* cell lines. fpkm = fragments per kilobase of exon per million fragments mapped.

Known members of the retinoid pathway are likely to be major mediators of ATRA anti-tumor activity. Given the respective associations with ATRA sensitivity and refractoriness observed in cell lines and primary tumors, we evaluated whether the *Luminal* and *Basal* phenotype as well as ER and HER2 positivity influence the expression of retinoid receptors/binding proteins. Both the microarray and the RNA-seq data associated with our panel of cell lines indicate that the average levels of RARα, RXRα, and CRABP2 are significantly higher in *Luminal* than *Basal* cells (Fig4), while FABP5 shows an opposite pattern. Thus, *Luminal* cells are predisposed to activate CRABP2/RARα upon ATRA challenge. In the context of *Luminal* cell lines, these mRNAs show the same expression profile in ER^+^ relative to ER^−^ cells. In the HER2^+^ cellular context (*SKBR3*, *AU565*, and *UACC812* cell lines), the data confirm that *RARA* co-amplification results in high levels of RARα (Paroni *et al*, 2012).

**Figure 5:**
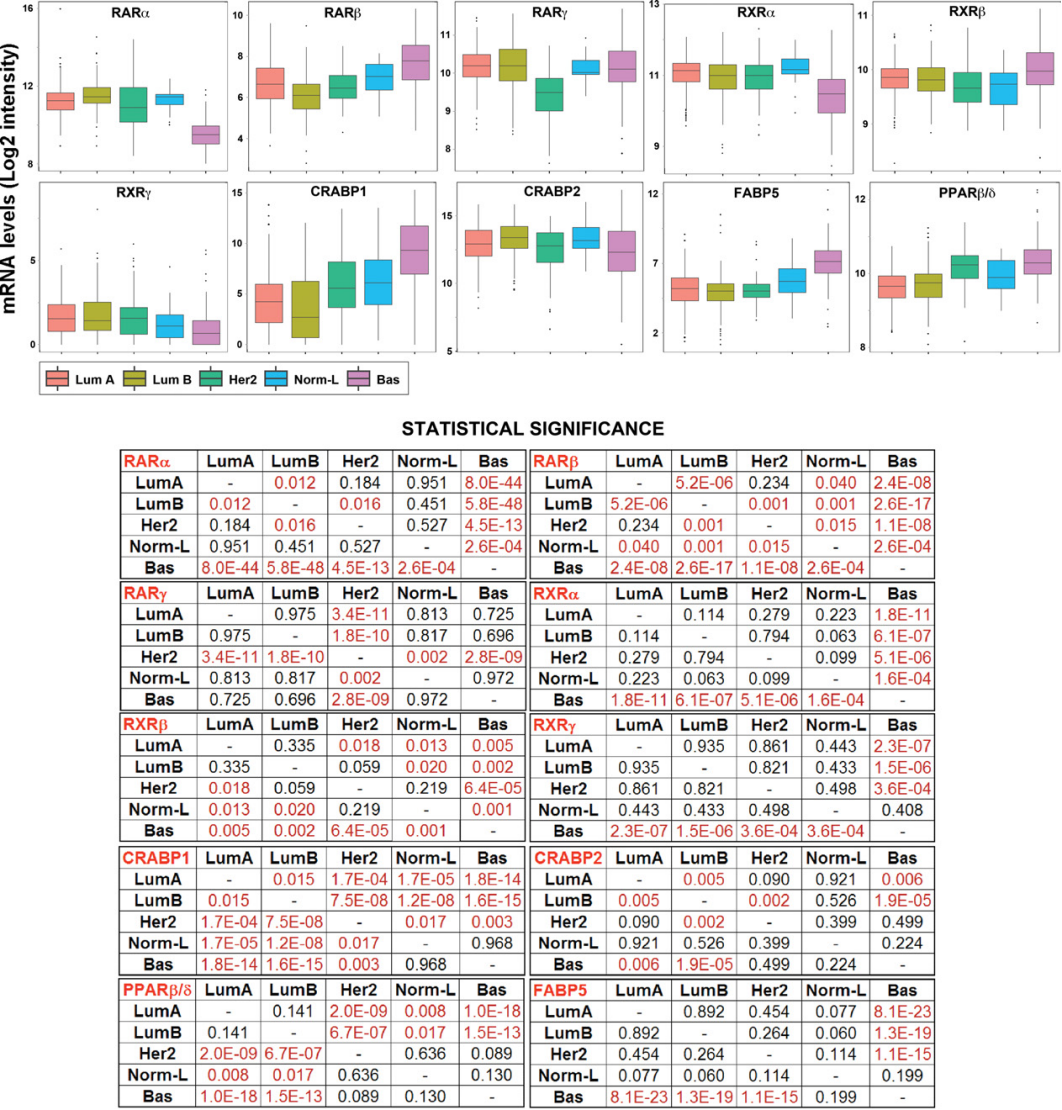
Associations between components of the retinoid pathway and breast cancer phenotype Associations between the expression of the indicated members of the retinoid pathway in the TCGA gene-expression database are shown. Mammary tumors are classified into *Luminal*-*A*, *Luminal-B*, *HER2-like*, *Normal-like*, and *Basal* according to the PAM50 fingerprint. The average expression levels and the corresponding SD values of the indicated members of the retinoid pathway are shown by the upper box plots. For each member of the retinoid pathway, significant differences between the indicated groups of tumors are shown in the lower table. Significant *P*-values for the indicated comparisons (Student's *t*-test) are shown in red.

To evaluate whether the expression patterns of retinoid receptors/binding proteins in cell lines recapitulate the situation in mammary tumors, we analyzed the TCGA RNA-seq dataset consisting of over 1,000 breast tumors classified into *Luminal-A*, *Luminal-B*, *HER2-like, Normal-like* and *Basal* according to PAM50. Consistent with the cell-line data, *Basal* tumors synthesize the smallest amounts of RARα, RXRα, and RXRγ and the highest levels of FABP5 (Fig5A and B). The analysis unmasks associations which are not evident in cell lines, that is, direct correlations between RARβ/RXRβ/CRABP1/PPARβ/δ expression and the *Basal* phenotype. Given the poor responsiveness of *Basal* cell lines to the retinoid (Fig1B), these results support the notion that FABP5 and PPARβ/δ are negative determinants of ATRA sensitivity (Balmer & Blomhoff, 2002; Kannan-Thulasiraman *et al*, 2010). As for RARβ, its expression does not seem to be important for ATRA anti-tumor activity in breast cancer (Connolly *et al*, 2013). In conclusion, our data demonstrate that RARα is the only receptor with a high level of expression in the cellular phenotypes predicted to be responsive to ATRA.

### Direct associations between RARα and ATRA sensitivity

**Figure 6:**
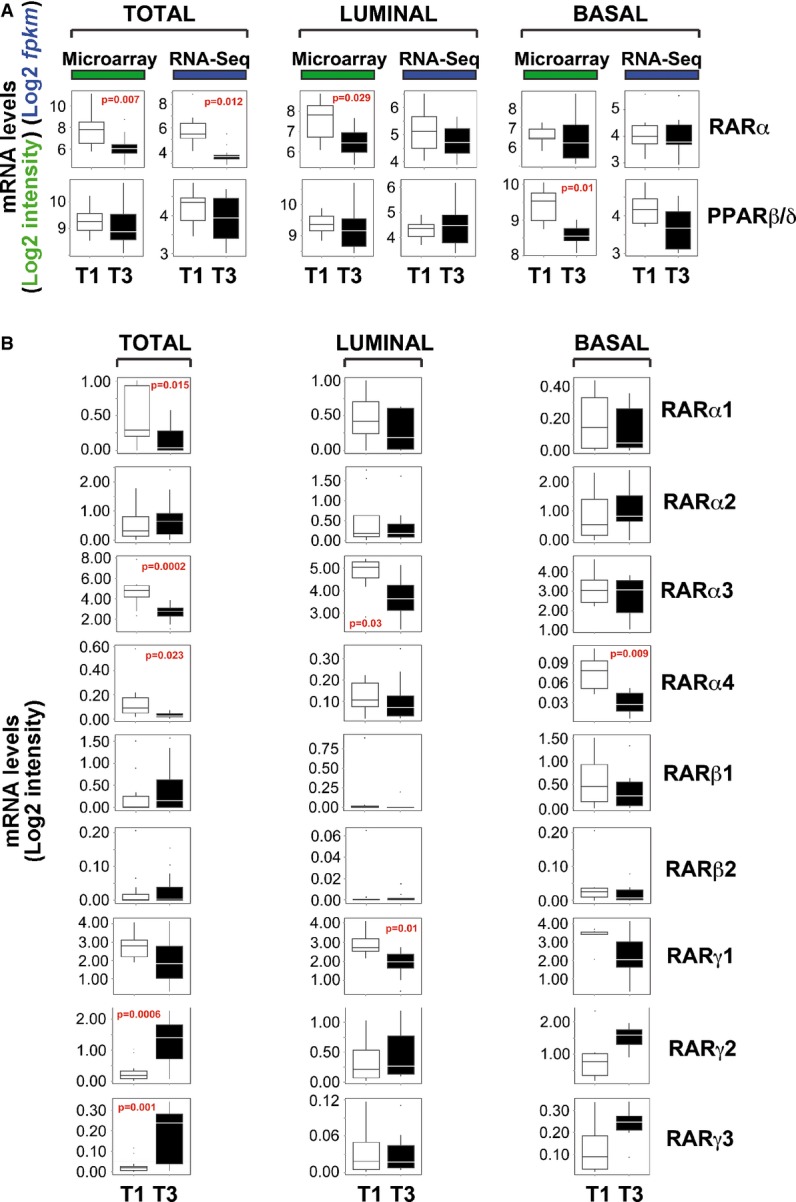
Associations between components of the retinoid signaling pathway and ATRA sensitivity

After grouping all the cell lines in ascending tertiles (T3 to T1) according to the *ATRA score*, we looked for associations between retinoid receptors/binding proteins and ATRA sensitivity. According to both the microarray and RNA-seq results, the average amounts of RARα are significantly higher in T1 (ATRA sensitive) than T3 (ATRA resistant) cell lines (Fig6A). No difference in the amounts of PPARβ/δ (Fig6A), RARβ, RARγ, RXRα, RXRβ, RXRγ, CRABP2, and FABP5 (Supplementary Fig S3) is evident. RARα is also significantly over-expressed in the T1 group, if the analysis of the microarray data is limited to the 22 *Luminal* cell lines and the trend is confirmed by RNA-seq, although the results do not reach statistical significance. In the microarray data, T1 cells express larger amounts of PPARβ/δ (Fig6A) than the T3 counterparts, after restriction to the *Basal* subset (20 cell lines). Thus, RARα is likely to be a determinant of ATRA sensitivity in both the total and *Luminal* fraction of cell lines, while PPARβ/δ may represent a positive factor in *Basal* cell lines.

The gene-expression microarray and RNA-seq data associated with 40 of the breast cancer cell lines were used for the analyses. The panels illustrate the associations of RARα and PPARβ/δ with ATRA sensitivity. The left panels show the basal average levels of the indicated transcript in the cell lines belonging to the T1 and T3 groups (13 cell lines in each of the T1 and T3 groups) defined by ascending *ATRA scores*. The intermediate and right panels indicate the same results after stratification for the *Luminal* (microarray and RNA-seq data = 7 cell lines in each of the T1 and T3 groups) and the *Basal* (microarray and RNA-seq data = 7 cell lines in each of the T1 and T3 groups) phenotype, respectively. fpkm = fragments per kilobase of exon per million fragments mapped.

The basal expression levels of the indicated RAR-isoform variants were determined with the use of specific Taqman assays. The results are associated with ATRA sensitivity before (TOTAL) and after stratification of the cell lines for the *Luminal* and *Basal* phenotype as in (A). Data information: Significant *P*-values (Student's *t*-test) are indicated in red.

### The complement of RAR splicing variants in cell lines and primary tumors: RARα3 as the major determinant of ATRA sensitivity

RARα (RARα1-4), RARβ (RARβ1-2-5), and RARγ (RARγ1-5) splicing variants are known (Supplementary Fig S4). Quantitative PCR was used to determine basal expression of these variants in our panel of cell lines. In the majority (40/42) of the cell lines (Supplementary Fig S5A), RARα3 is the most highly expressed RARα mRNA, being at least one order of magnitude more abundant than RARα2 and RARα1. Extremely low levels of RARα4 are generally observed. Only RARα3 and RARα4 show significant co-regulation across the panel (Supplementary Fig S5B), consistent with transcriptional control by the same promoter. *Luminal* cells contain significantly larger amounts of RARα3, RARα1, and RARα4 mRNAs than the *Basal* counterparts (Table1). Higher levels of the same transcripts are also associated with ER positivity, although statistical significance is not reached if analysis is restricted to the *Luminal* cell lines. If associations between RARα variants and ATRA sensitivity are searched for, significant over-expression of RARα3 in T1 relative to T3 cell lines is evident (Fig6B). A similar, though less significant, association is observed with RARα1 and RARα4. After stratification for the *Luminal*/*Basal* phenotype, the association between RARα3 and ATRA sensitivity is maintained solely in *Luminal* cells.

**Table 1:** Associations between the RARα splicing variants and the phenotype in breast cancer cells

	RARα1 (mean ± SE)	RARα2 (mean ± SE)	RARα3 (mean ± SE)	RARα4 (mean ± SE)
*Basal* cell lines (A)	0.12 ± 0.03	1.11 ± 0.29	6.92 ± 1.23	0.03 ± 0.01
*Luminal* cell lines (B)	0.34 ± 0.08	0.54 ± 0.16	30.98 ± 10.04	0.09 ± 0.02
*t*-test A versus B	*P* = 0.01	*P* = 0.09	*P* = 0.02	*P* = 0.01
ER^+^ cell lines (C)	0.40 ± 0.10	0.64 ± 0.20	39.73 ± 15.39	0.12 ± 0.04
ER^−^ cell lines (D)	0.15 ± 0.04	0.90 ± 0.22	9.42 ± 1.83	0.03 ± 0.01
ER^−^ *Luminal* cell lines (E)	0.23 ± 0.14	0.36 ± 0.26	15.68 ± 5.46	0.04 ± 0.02
*t*-test C versus D	*P* = 0.02	*P* = 0.39	*P* = 0.05	*P* = 0.03
*t*-test C versus E	*P* = 0.27	*P* = 0.39	*P* = 0.15	*P* = 0.06
*t*-test D versus E	*P* = 0.47	*P* = 0.06	*P* = 0.13	*P* = 0.38
HER2^+^ cell lines (F)	0.27 ± 0.11	0.28 ± 0.11	38.69 ± 18.58	0.09 ± 0.04
HER2^−^ cell lines (G)	0.22 ± 0.05	1.02 ± 0.21	11.86 ± 1.92	0.05 ± 0.01
HER2^+^ *Luminal* cell lines (H)	0.27 ± 0.12	0.31 ± 0.14	43.54 ± 22.26	0.10 ± 0.05
HER2^−^ *Luminal* cell lines (I)	0.35 ± 0.11	0.73 ± 0.26	20.35 ± 3.43	0.09 ± 0.02
*t*-test F versus G	*P* = 0.66	*P* = 0.003	*P* = 0.16	*P* = 0.39
*t*-test F versus H	*P* = 0.99	*P* = 0.86	*P* = 0.86	*P* = 0.86
*t*-test F versus I	*P* = 0.58	*P* = 0.13	*P* = 0.33	*P* = 0.97
*t*-test G versus H	*P* = 0.71	*P* = 0.006	*P* = 0.17	*P* = 0.35
*t*-test G versus I	*P* = 0.27	*P* = 0.38	*P* = 0.04	*P* = 0.18
*t*-test H versus I	*P* = 0.60	*P* = 0.16	*P* = 0.30	*P* = 0.86

The table shows the associations between *RAR*α1-4 and the *Luminal* versus *Basal* phenotype, ER positivity versus ER negativity, as well as HER2 positivity versus HER2 negativity. *P*-values for the indicated comparisons (Student's *t*-test) are shown.

We determined RARβ1 expression and combined expression of RARβ2 and 5, as the last two variants code for the same protein. RARβ1 is the most abundant species, although the transcript is detectable only in 15 of 42 cell lines (Supplementary Fig S6A). Despite regulation by distinct promoters, RARβ1 and RARβ2/5 are always co-expressed (Supplementary Fig S6B). Both RARβ1 and RARβ2/5 are over-expressed in *Basal* relative to *Luminal* cells. The same is true in ER^−^ versus ER^+^ cell lines, even if the analysis is restricted to the *Luminal*/ER^−^ group (Supplementary Fig S6C). Regarding possible associations with ATRA sensitivity, no significant difference in the levels of RARβ1 or RARβ2/5 between the T1 and T3 cell lines is evident before or after stratification for the *Luminal*/*Basal* phenotype (Fig6B).

As for RARγ, we focused our attention on RARγ1-3, which are predicted to code for active transcription factors. The order of expression for the RARγ forms is RARγ1 >> RARγ2 > RARγ3 (Supplementary Fig S7A). Consistent with regulation by the same promoter, only the RARγ2/RARγ3 couple is characterized by co-regulation across all the cell lines (Supplementary Fig S7B). As the RARβ variants, RARγ2 and RARγ3 show a direct association with the *Basal* and ER^−^ phenotypes (Supplementary Fig S7C), while only RARγ3 is significantly higher in HER2^−^ than HER2^+^ cells. In addition, RARγ2 and RARγ3 tend to be over-expressed in the T3 relative to the T1 group (Fig6B), supporting the idea that they represent negative factors in terms of ATRA sensitivity (Bosch *et al*, 2012).

Taken together, the results point to RARα3 as the principal element of the retinoid pathway mediating the anti-proliferative responses of *Luminal* cells to ATRA.

### RARα3 as a major player of ATRA sensitivity in primary tumors

**Figure 7:**
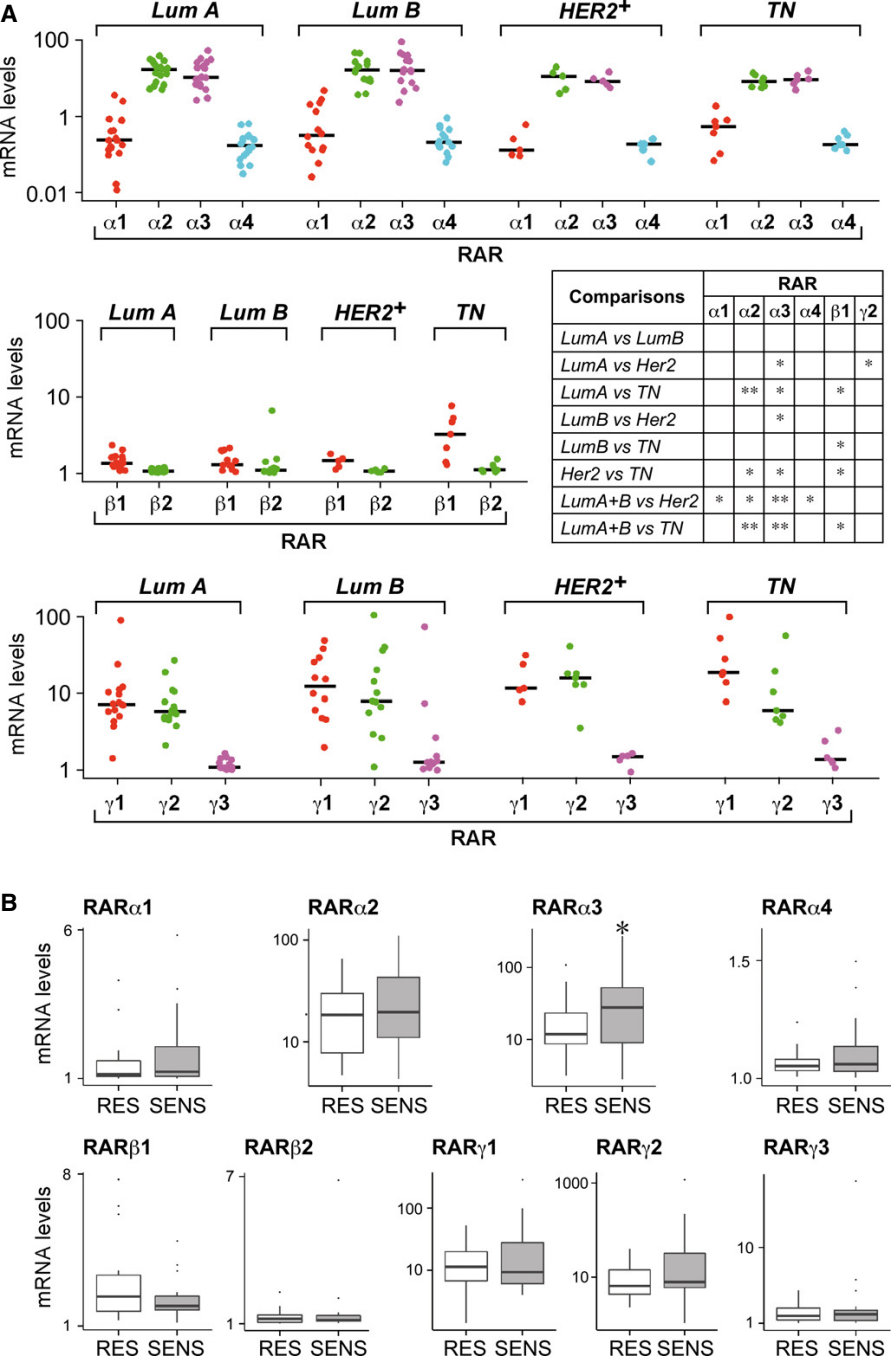
Basal levels of RARα, RARβ, and RARγ mRNA splicing variants in mammary tumors Total RNA was extracted from the tissue slices deriving from the surgical specimens of breast cancer patients used in Fig3 before any treatment with DMSO or ATRA. RNA was subjected to RT–PCR analysis to determine the basal expression of the indicated RAR splicing variants.
Each value represents the mean ± SD of two replicate measurements. The table shows the statistical significance of the indicated comparisons. *Significantly different (*P*-value < 0.05, Student's *t*-test). **Significantly different (*P*-value < 0.01, Student's *t*-test).

The profiles of expression of the RAR splicing variants were defined in the primary tumors used to evaluate ATRA sensitivity (see Fig3). In all the specimens considered, RARα3 and RARα2 are the most abundant RARα mRNAs and have a similar level of expression (Fig7A), which is different from what is observed in the cell lines. Across all the samples, RARβ1 is more abundant than RARβ2, although RARβ1 levels are at least one order of magnitude lower than the RARα3/RARα2 counterparts. In the case of the RARγ variants, RARγ1 and RARγ2 show intermediate levels of expression relative to RARα3/RARα2 and RARβ1/RARβ2. RARγ2 in primary tumors is more abundant than expected from the cell-line results, while RARγ3 is by far the least abundant species. The expression of RARα3/RARα1, RARα3/RARα2, RARα3/RARα4, RARα2/RARα4, RARγ1/RARγ2, RARγ1/RARγ3, and RARγ2/RARγ3 across the tumor samples is highly correlated (Supplementary Fig S8).

The plots illustrate the average expression levels of the indicated mRNAs (mean ± SD of two replicates) in tumor samples classified as ATRA-sensitive (Sens) and ATRA-resistant (Res) according to the response of Ki67. *Significantly different (*P*-value < 0.05, Student's *t*-test).

As for possible associations between RAR splicing variants and tumor cell phenotype, in accordance with the cell-line data, the content of RARa3 is generally higher in *Luminal*, relative to *TN* cancers and HER2^+^ tumors with no co-amplification of the *ERBB2* and *RARA* loci (Fig7A). In the case of the RARβ variants, expression is similar in the tissue samples and cell lines, as the average levels of RARβ1 are significantly more abundant in *TN* (*Basal*) than in all the other tumor sub-types (Fig7A). In terms of possible associations between RAR splicing variants and ATRA sensitivity, only the levels of RARα3 are significantly higher in sensitive than in refractory tumors (Fig7B).

In conclusion, the complement of RAR splicing variants in breast tumors and derived cell lines is not entirely superimposable. Nevertheless, the profiles of RAR splicing variants in our cohort of mammary tumors support a major role of RARα3 in the anti-tumor responses to ATRA, which is in line with the conclusions drawn in cell lines.

### RARα protein and ATRA sensitivity in breast cancer cell lines

**Figure 8:**
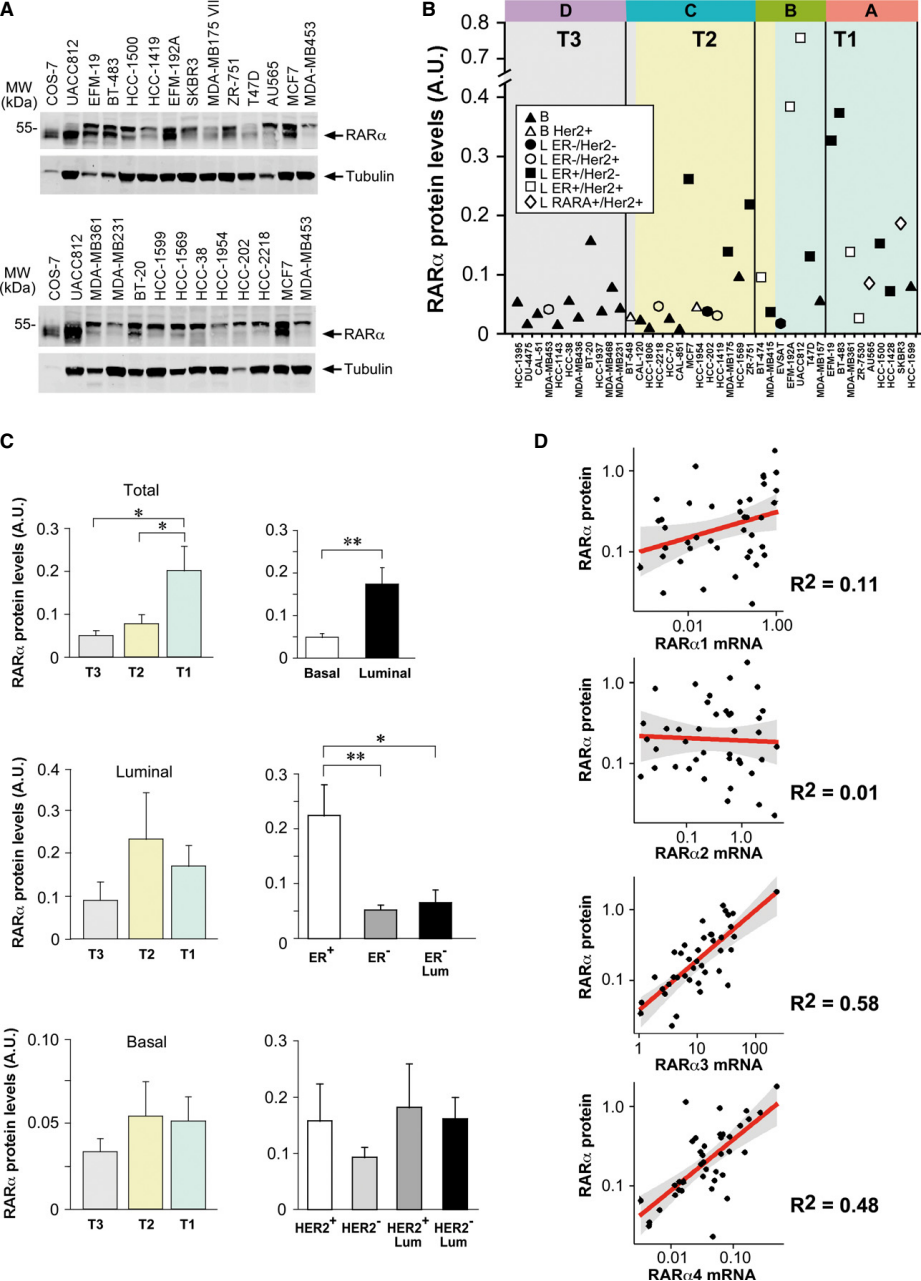
RARα protein and ATRA sensitivity in breast cancer cell lines Total proteins were extracted from logarithmically growing cell lines and subjected to Western blot analysis.
The panel illustrates representative Western blots for a number of cell lines. The blots were sequentially developed with RARα-specific and control tubulin antibodies. The positions of the RARα and tubulin bands (right) along with the position of a relevant molecular weight marker (left) are indicated. To normalize the Western blot signals in different gels, the same preparation of RARα-transfected *COS-7* cell extracts (COS-7) and *MCF7* extracts was loaded in each gel. The blots are representative of at least two independent experiments providing similar results.

Given the observed relevance of the RARα3 transcript in our models, the basal levels of the corresponding RARα protein were determined in breast cancer cell lines with a specific antibody (Fig8A and B). The average levels of RARα are significantly higher in T1 versus T3 *ATRA score* groups (Fig8C), and the same trend is observed if the analysis is restricted to *Luminal* cells. Larger amounts of RARα are also observed in *Luminal* versus *Basal* and ER^+^ versus ER^−^ cell lines. As for possible correlations with the RARα mRNA variants across the cell lines, the highest *R*^2^ values were calculated for the RARα protein and the RARα3/RARα4 mRNAs (Fig8D). This indicates that the protein is encoded by either the RARα3 or the RARα4 transcript. Given the low relative expression levels of RARα4, we favor RARα3.

The quantitative results obtained after densitometric analysis of the RARα bands are plotted against the *ATRA scores*. Cell lines are grouped according to the *ATRA score* (A–D groups and T1–T3 tertiles).

Left: The upper graph indicates the averages expression levels of the RARα protein in T1–T3 cell lines. The middle and lower graphs indicate the average expression levels after stratification of the cell lines for the *Luminal* (six cell lines in each of the T1 and T3 groups) and the *Basal* (six cell lines in each of the T1 and T3 groups) phenotype, respectively. Right: The levels of the RARα protein in *Basal* and *Luminal* cell lines (upper graph), in ER^+^, ER^−^*,* and *Luminal* ER^−^ cell lines (middle graph) and the indicated classes of HER2^+^ and HER2^−^ cell lines (lower graph) are shown. *Significantly different (*P*-value < 0.05, Student's *t*-test). **Significantly different (*P*-value < 0.01, Student's *t*-test).

The plots show the correlation curves between the levels of the indicated RARα-variant transcripts and the RARα protein.

### Effects of RAR agonists/antagonists in breast cancer cells

**Figure 9:**
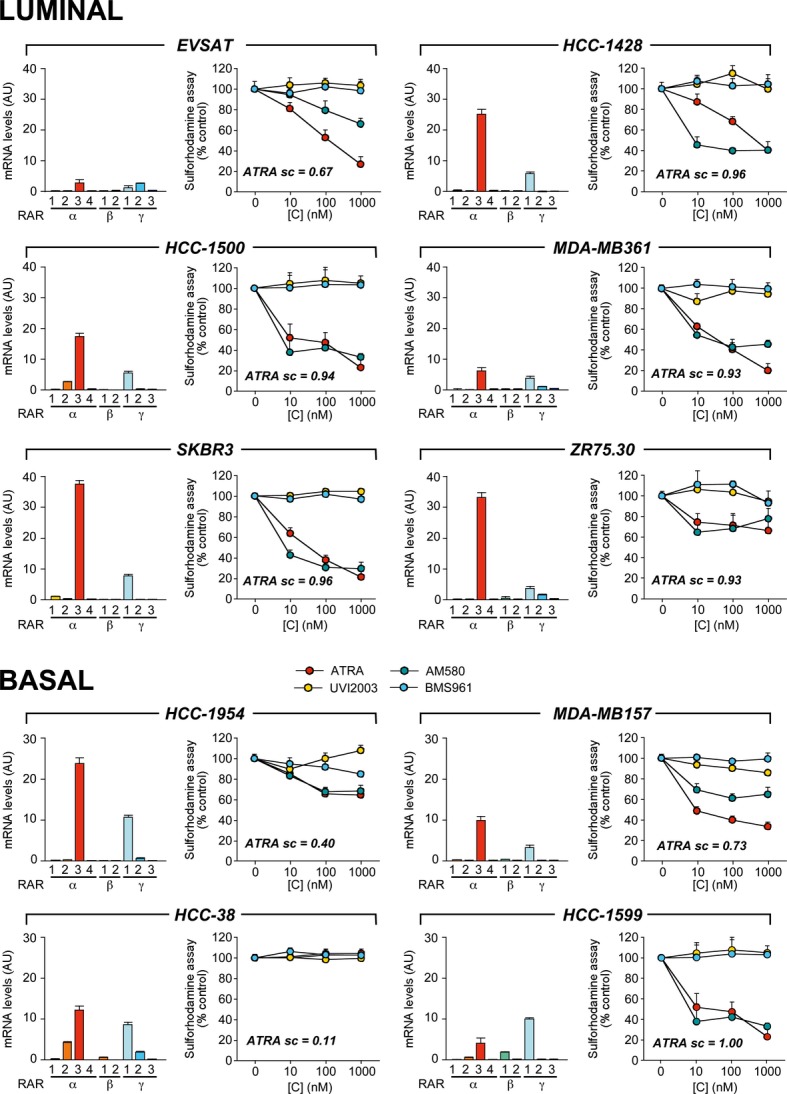
Effects of RAR agonists on the growth of Luminal and Basal breast cancer cell lines The indicated *Luminal* and *Basal* cell lines were challenged with increasing concentrations of ATRA, the RARα agonist, AM580, the RARβ agonist, UVI2003, and the RARγ agonist, BMS961, for 6 days. The complement of RAR-variant transcripts expressed in each cell line is shown in the left bar graphs (mean ± SD of two replicate measurements). The growth curves (sulforhodamine assay) of the cell lines are illustrated by the right linear plots. The results are expressed in % values relative to the corresponding control dishes treated with vehicle alone (right graphs). Each result is the mean ± SD of five replicate wells. ATRA sc = ATRA score.

The functional role of RARα in the anti-proliferative action of ATRA was evaluated in *Luminal* and *Basal* cell lines with different *ATRA scores* and RAR-variant expression profiles (Fig9) with a pharmacological approach, using the validated (Supplementary Fig S9) AM580 RARα agonist (Gianni *et al*, 1996), the UVI2003 RARβ agonist (Alvarez *et al*, 2014), and the BMS961 RARAγ agonist (Gianni *et al*, 1993). The cell lines were challenged with increasing concentrations of ATRA, AM580, UVI2003, and BMS961 for 3 (data not shown) and 6 days prior to evaluation of cell growth. In the ATRA-sensitive *Luminal* lines, AM580 is the only agonist which inhibits growth in a dose-dependent manner. In ER^+^/HER2^−^ *HCC-1428* cells, AM580 is more effective than ATRA, while the opposite is true in the ER^−^/HER2^−^ *EVSAT* counterpart. In the remaining *Luminal* lines, AM580 and ATRA show similar efficacy. AM580 is also the sole agonist inhibiting the growth of the retinoid-sensitive *Basal* cell lines, *HCC-1599*, *MDA-MB157*, and *HCC-1954*. In these cellular contexts, no significant difference in the anti-proliferative activity of AM580 and ATRA is noticeable. AM580, UVI2003, BMS961, and ATRA are equally ineffective in retinoid-resistant *HCC-38* cells.

To corroborate the results obtained with the RAR agonists, we evaluated the effects of the RARα antagonist, ER50891 (Kikuchi *et al*, 2001; Somenzi *et al*, 2007), and the RARβ/γ antagonist, CD2665 (Szondy *et al*, 1997), on ATRA-dependent growth inhibition of *HCC-1428* and *SKBR3* cells, which are characterized by very high *ATRA scores*. To obtain maximal blockade of the two RARs without off-target effects, cells were treated with 100 nM ATRA and 3 μM of ER50891 or CD2665, as this concentration of the antagonists blocks the trans-activating potential of ATRA in a RARα- and RARβ/γ-specific fashion, respectively (Supplementary Fig S10). In *HCC-1428* and *SKBR3* cells, only ER50891 blocks the anti-proliferative action of ATRA (Supplementary Fig S11).

### RARα and ATRA sensitivity: over-expression and knock-down studies

**Figure 10:**
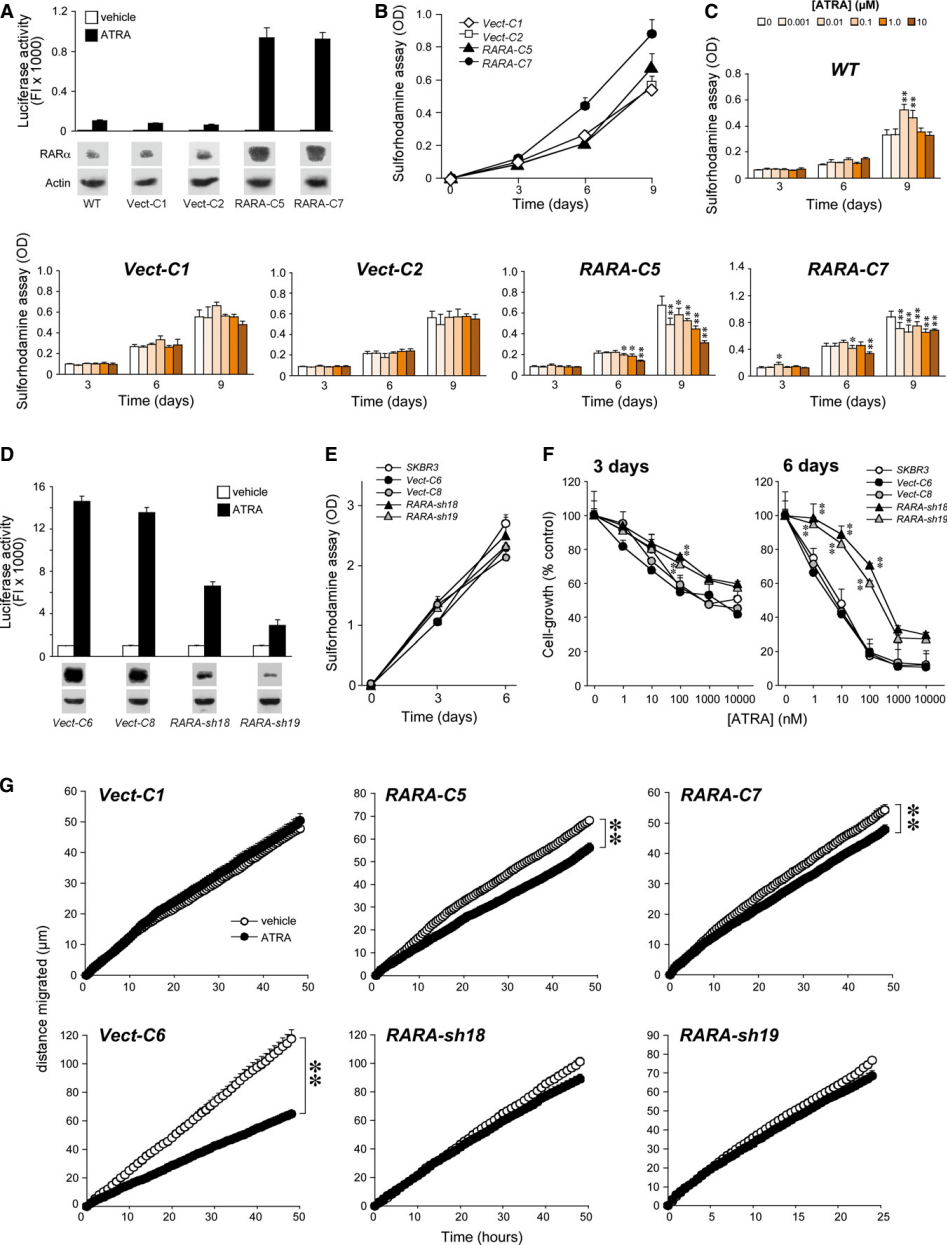
Over-expression of RARα1/3 in ATRA-resistant *MDA-MB453* cells and silencing of RARα1/3 in ATRA-sensitive SKBR3 cells A RARα1/3 plasmid construct and the corresponding control void vector were stably transfected into ATRA-resistant *MDA-MB453* cells. Two cell clones over-expressing RARα (*RARA-C5* and *RARA-C7*) and two appropriate control clones (*Vect-C1* and *Vect-C2*) were isolated. A RARα3 shRNA plasmid construct and the corresponding void vector were stably transfected into ATRA-sensitive *SKBR3* cells. After selection, two cell clones silenced for RARα3 (*RARA-sh18* and *RARA-sh19*) and two appropriate control clones (*Vect-C6* and *Vect-C8*) were isolated.
A, D The indicated clones and the parental cell line (*WT*) were transiently transfected with the *RARE-DR5-Luc* retinoid reporter construct and the level of luciferase activity was measured 24 h after treatment with vehicle (DMSO) and ATRA (100 nM), as illustrated in the upper bar graph. Each value is the mean ± SD of three replicate cultures. The levels of the RARα protein measured in the indicated clones by Western blot analysis is shown under the bar graph. To demonstrate that similar levels of total proteins were loaded in each lane, the β-actin band signal obtained after re-blotting of the gel is shown. FI = Fluorescence intensity.

To obtain direct proof that the RARα3 protein is mediating the action of ATRA, we over-expressed it in retinoid-resistant, HER2^+^/ER^−^, and *Luminal MDA-MB453* cells. Two RARα-over-expressing (*RARA-C5* and *RARA-C7*), two vector-transfected control (*Vect-C1* and *Vect-C2*) clones, and the parental *MDA-MB453* cells (*WT*) were used in comparative experiments. *WT*, *Vect-C1*, and *Vect-C2* express barely detectable levels of the RARα protein, while large amounts of the product are synthesized by *RARA-C5* and *RARA-C7* cells (Fig10A). *RARA-C5* and *RARA-C7* express a transcriptionally active RARα form, as indicated by ATRA-dependent activation of the luciferase-based retinoid reporter, *DR5-RARE-Luc*. Over-expression of RARα does not exert major effects on the basal growth rate of the *MDA-MB-453* clones (Fig10B). Upon treatment with increasing concentrations of ATRA for 3, 6, and 9 days, *Vect-C1* and *Vect-C2* and *WT* cells are equally unresponsive to retinoid-dependent growth inhibition (Fig10C). In contrast, *RARA-C5*/*RARA-C7* proliferation is inhibited dose- and time-dependently by ATRA. Thus, stable over-expression of RARα renders *MDA-MB-453* cells sensitive to the retinoid with an ∼4-fold increase in the calculated *ATRA score* at 9 days.

B, E The panels illustrate the growth curves of the indicated *MDA-MB453* and *SKBR3* clones and the *SKBR3* parental cell lines (*WT*) measured with the sulforhodamine assay.

C The bar graphs illustrate the effect of increasing concentrations of ATRA on the growth of the indicated *MDA-MB453* clones and the parental cell line. The cell lines were challenged with vehicle (DMSO) or ATRA for 3, 6, and 9 days prior to the sulforhodamine assay. OD = optical density at 540 nm. Each value is the mean ± SD of five replicate culture wells.

F The graphs illustrate the effect of increasing concentrations of ATRA on the growth of the indicated *SKBR3* clones and the parental cell line. The cell lines were challenged with vehicle (DMSO) or ATRA for 3 and 6 days prior to the sulforhodamine assay. Each value is the mean ± SD of five replicate culture wells.

G The panel illustrates the effects exerted by ATRA (0.1 μM) on random cell motility of the indicated *MDA-MB453* and *SKBR3* clones. The results are representative of two independent experiments. Each value is the mean ± SE of the motility of at least 60 cells. Data information: * and **, significantly different from the corresponding vehicle-treated control (*P-value < 0.05, **P-value < 0.01, Student's *t*-test).

In a mirror series of experiments, we knocked down RARα in the retinoid-sensitive HER2^+^/ER^−^ and *Luminal SKBR3* cells by stable transfection of a RARα1/3-targeting shRNA. The two shRNA-transfected *RARA-sh18* and *RARA-sh19* clones express < 10% of the RARα protein levels in the parental (data not shown) and void vector-transfected *Vect-C6* or *Vect-C8* cells (Fig10D). Transfection of *DR5-RARE-Luc* in *RARA-sh18* and *RARA-sh19* cells demonstrates inhibition of ATRA-dependent transcriptional activity relative to the *Vect-C6* or *Vect-C8* counterparts (Fig10D). While the shRNA constructs and void vectors do not alter the basal growth rate of *SKBR3* cells (Fig10E), RARα knockdown attenuates the anti-proliferative action of ATRA (Fig10F). Attenuation is observed at concentrations of ATRA between 0.001 and 0.1 μM and tends to be lost at the two highest concentrations considered, where ATRA exerts off-target effects.

We evaluated whether modulation of RARα has any effect on ATRA-dependent expression of four direct retinoid target genes. ATRA-dependent induction of CYP26A1, CYP26B1, BTG2, and RARRES3 is not observed in *Vect-C2* cells (Supplementary Fig S12A). In contrast, ATRA induces the expression of the first three transcripts in *RARA-C5* cells. Similar differential effects are observed if the levels of the two retinoid-dependent epithelial differentiation markers, β-catenin and SMAD3 (Paroni *et al*, 2012), are measured in *Vect-C1*, *Vect-C2*, *RARA-C5*, and *RARA-C7* cells before and after treatment with ATRA (Supplementary Fig S12B). Conversely, ATRA-dependent induction of the CYP26A1, CYP26B1, BTG2, and RARRES3 mRNAs as well as the β-catenin and SMAD3 proteins observed in *Vect-C8* cells is blocked in *RARA-Sh18* cells. Thus, RARα is the predominant mediator not only of the anti-proliferative, but also of the transcriptional effects afforded by ATRA in the two breast cancer cells.

To determine whether RARα mediates other ATRA-dependent responses of relevance for the anti-tumor activity of the retinoid, we measured single-cell random motility, as the process is a determinant of invasive/metastatic behavior and it is inhibited by ATRA in breast cancer cells (Terao *et al*, 2011). As expected from the results in parental lines (data not shown), *MDA-MB453*-derived *Vect-C1* cells are unresponsive to the anti-motility action of ATRA (Fig10G), while *SKBR3*-derived *Vect-C6* cells respond with a significant reduction in random motility. RARα over-expression sensitizes *RARA-C5* and *RARA-C7* cells to ATRA, while RARα knockdown induces ATRA resistance in *RARA-sh18* and *RARA-sh19* cells. These data support a key role of RARα in mediating the anti-metastatic activity of ATRA.

### Identification of gene sets associated with ATRA sensitivity

Besides RARα, other gene products are likely to play a role in the anti-tumor action of ATRA. Thus, we looked for genes whose basal levels of expression are correlated to the *ATRA score* across our panel of cell lines using the microarray/RNA-seq databases and a regressed Random Forest approach (Supplementary Fig S13). The goal was the generation of ranked lists of genes associated with ATRA sensitivity based on the variable importance scores (Supplementary Table S4).

**Figure 11:**
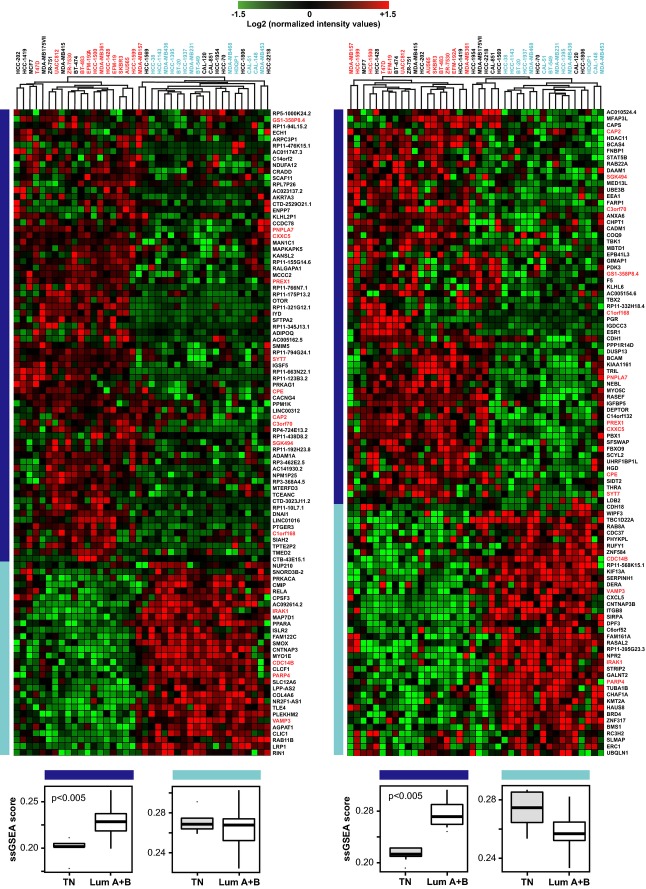
Gene sets associated with ATRA sensitivity Using the microarray and RNA-seq data associated with the breast cancer cell lines, two *ATRA score*-associated gene lists ranked for their variable importance were generated. Upper Panels: The gene-expression results of the first 100-ranking genes in the RNA-seq (left) and microarray (right) datasets were used to perform a cluster analysis of the breast cancer cell lines according to the gene-expression profiles. Data are expressed using a log_2_ scale of the expression signal intensity after normalization of the data across the different cell lines. The genes marked in red are present in both the microarray and the RNA-seq gene sets. The cell lines marked in red are those belonging to the *ATRA score* T1 group and are sensitive to ATRA, while the ones marked in blue belong to the T3 group and are refractory to the retinoid. The left dark blue lines indicate the genes with higher levels of constitutive expression in the ATRA-sensitive cell lines, while the light blue lines indicate the genes with higher levels of basal expression in the ATRA-refractory cell lines. Lower Panels: The box plots show the enrichment score (single sample Gene Set Enrichment Analysis, ssGSEA) of the microarray (left) and RNA-seq (right) gene sets in the *TN* (patients 9, 22, 23, 31, 50) and *Lum* (patients 13, 18, 27, 36, 41, 44, 55, 60, 61, 62, 64) tumors cultured in the absence of ATRA for 48 h. The *P*-values of the enrichment are indicated.

We generated two distinct expression heat-maps of the top 100 RNA-seq (Fig11, left) and microarray (Fig1, right) genes associated with ATRA sensitivity in the two databases. Fourteen of the genes are common to the microarray and RNA-seq gene sets. This is a high proportion considering the large difference in the quantifiable gene products between the two datasets (RNA-seq = 57,789; microarray = 15,543). Cluster analysis of both the microarray and RNA-seq data allows a clear separation of the lines belonging to the T1 and T3 groups identified by the *ATRA score*. Although our gene sets may contain elements specific to *Luminal* or *Basal* cell lines, as indicated by the presence of three PAM50 genes (estrogen receptor 1, *ESR1*; progesterone receptor, *PGR*; CXXC finger protein 5, *CXXC5*), it must be noticed that they do not simply stratify the cell lines according to the *Luminal* or *Basal* phenotype. To validate the expression results with an independent assay, we performed quantitative real-time PCR on 14 selected genes. The PCR, microarray, and RNA-seq results are concordant (Supplementary Fig S14).

By far the highest ranking retinoid nuclear receptor in the list generated from the microarray data is RARα, standing within the first 3.6% of the ranked mRNAs. RARα is also highly ranked in the RNA-seq list (top 16.5%). Some of the 100 top-ranking genes present in both lists may be of interest for the anti-tumor action of ATRA. For instance, *CXXC5* is a retinoid-inducible gene (Knappskog *et al*, 2011; Astori *et al*, 2013), and it mediates the proliferative responses of IGFs and HER2 in breast cancer (Montero *et al*, 2011, 2013). The ATRA-regulated SYT7 (Synaptotagmin-VII) (Ekici *et al*, 2008) and VAMP3 (vesicle-associated-membrane protein-3) proteins control the homeostasis of micro-vesicles, which, in turn, regulate mammary tumorigenesis (Wright *et al*, 2009). A splicing variant of *CPE* (carboxypeptidase-E) stimulates growth, and it is a biomarker for mammary tumor metastatic spread (Lee *et al*, 2011). In *IRAK1* (interleukin-1-receptor-associated kinase-1) knockout macrophages, RARα expression is higher than in the parental counterparts (Maitra *et al*, 2009).

The two identified gene sets may be useful for the stratification of patients who are likely to benefit from retinoid-based therapeutic approaches. We evaluated whether primary *Luminal* tumors, which are generally responsive to the anti-proliferative action of ATRA, are enriched for elements present in both gene sets (Fig1, bottom), comparing the 11 *Luminal* and 5 *TN* tumors used to assess the genomic effects of ATRA (see Fig2). The enrichment (single sample Gene Set Enrichment Analysis, ssGSEA) of genes whose basal levels of expression is higher in ATRA-sensitive (T1 group) than in ATRA-refractory (T3 group) cell lines is significantly higher in *Luminal* relative to *TN* tumors. In contrast, the enrichment of genes whose basal levels of expression is lower in the T1 than in the T3 group tends to be lower in *Luminal* than *TN* tumors. The two identified gene sets are the basis for the generation of an optimized gene signature predictive of ATRA sensitivity.

**Figure 12:**
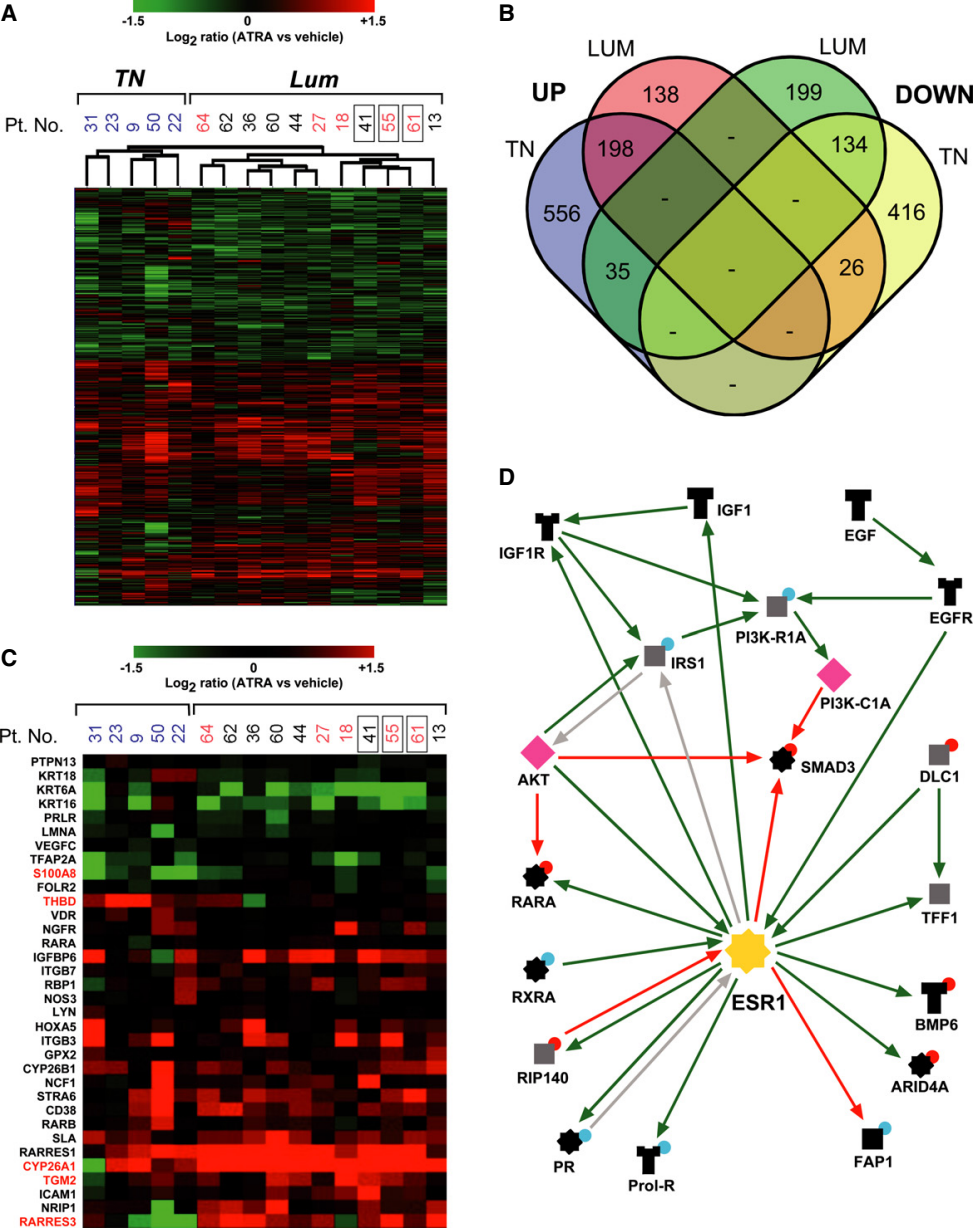
ATRA-dependent perturbations of the transcriptome in primary tumors Tissue slices corresponding to the indicated patients were treated with vehicle (DMSO) or ATRA (0.1 μM) for 48 h. Whole-genome gene expression studies were performed on the extracted total RNA using a microarray platform.
The heat-map shows the genes significantly up- or down-regulated by ATRA (*P* < 0.005, paired *t*-test) in either *Luminal-A* and *-B* (*Lum*) or triple-negative (*TN*) tumors, and the results are expressed as the log_2_ ratio observed between the ATRA and vehicle-treated samples.

A Venn diagram of the genes up- or down-regulated by ATRA in *TN* and *Lum* tumors is shown. The number of genes commonly or selectively regulated in *TN* and *Lum* tumors is indicated.

The heat-map shows the regulation patterns of the retinoid-dependent genes significantly modulated by ATRA (*P* < 0.001) in either *TN* or *Lum* tumors. The symbols highlighted in red represent the five genes differentially and significantly regulated by ATRA in *Lum* versus *TN* tumors.

The panel shows the estrogen-receptor (ESR1) pathway, which is significantly enriched for genes regulated by ATRA in *Lum* tumors. The green arrows indicate up-regulatory or stimulating interactions, while the red arrows indicate down-regulatory or inhibitory interactions. The gray arrows indicate unknown types of interactions. The red and blue dots above the protein symbols indicate the effect of ATRA in *Lum* tumors (red = up-regulation; blue = down-regulation).

### Transcriptional responses to ATRA in short-term cultures of mammary tumors

The results obtained in the cell lines and the short-term cultures of primary tumors support the concept that *Luminal* and ER^+^ phenotypes are positive determinants, while *Basal* and ER^−^ phenotypes are negative determinants of sensitivity to the anti-proliferative action of ATRA. To evaluate the transcriptional effects of the retinoid, we performed microarray gene-expression studies in 16 of the 45 primary-tumor samples profiled with the Ki67 biomarker (see Fig3). The cohort analyzed consisted of 11 *Luminal* and 5 *TN* cases. Microarray data were validated by quantitative PCR on a selected number of tumors and genes (*CYP26A1*, *CYP26B1*, *RARRES3*, and *BTG2*) (Supplementary Fig S15).

ATRA exerts major quantitative effects on the transcriptomes not only of *Luminal*/ER^+^ tumors, but also of *TN* cancers (Fig12A and Supplementary Table S5). Cluster analysis of the regulated transcripts results in a clear separation between *Luminal*/ER^+^ and *TN* tumors on the basis of the genomic responses to ATRA. It is interesting to notice that cluster analysis groups together the three Ki67-unresponsive *Luminal*/ER^+^ cases (Patients No. 41, 55 and 61). A total of 1,702 genes are significantly regulated by ATRA (*P* < 0.005, paired *t*-test) in either *TN* or *Luminal*/ER^+^ tumors. Approximately 20% of the genes (up-regulated genes = 198; down-regulated genes = 134) are similarly modulated by ATRA in both the *Luminal*/ER^+^ and *TN* cases (Fig2B).

We focused our attention on the subset of genes identified as retinoid targets in various cell types (Balmer & Blomhoff, 2002, 2005; Topletz *et al*, 2015). Among these 402 genes, 34 are significantly up- or down-regulated by ATRA (*P* < 0.001, paired *t*-test) in tissue slices derived from either *Luminal*/ER^+^ or *TN* tumors (Fig2C). Five of these genes are differentially regulated in *Luminal*/ER^+^ and *TN* tumors. The up-regulation of RARRES3 (retinoic acid receptor responder 3), TGM2 (transglutaminase 2), S100A8 (S100 calcium binding protein A8), and CYP26A1 (cytochrome P-450 26A1) is significantly higher in *Luminal*/ER^+^ tumors. In contrast, THBD (thrombomodulin) is significantly up-regulated only in *TN* tumors. *RARRES3* up-regulation in *Luminal*/ER^+^ may play a role in the anti-motility and anti-metastatic effects of ATRA (Nwankwo, 2002; Terao *et al*, 2011), as the factor has been shown to suppress metastases to the lung in breast cancer (Errico, 2014; Morales *et al*, 2014). In contrast, increased induction of *CYP26A1* may be detrimental for the anti-tumor action of ATRA, as the enzyme metabolizes and inactivates the retinoid (Thatcher *et al*, 2010; Topletz *et al*, 2012).

To define the biochemical pathways regulated by ATRA and potentially involved in the anti-tumor action of the retinoid, we performed gene-network enrichment analysis of the microarray data, focusing on *Luminal*/ER^+^ tumors. Among the top 10 processes enriched (Supplementary Table S6), the ER nuclear signal transduction pathway is of interest for its role in *Luminal* breast cancer growth. For instance, down-regulation of IRS1 and one of the regulatory subunit of PI3K by ATRA are likely to block the proliferation of *Luminal*/ER^+^ tumors caused by the growth factors IGF1 and EGF. Out of the 28 types of interactions between couples of proteins belonging to the ER pathway (Supplementary Table S7), 17 are consistent with an inhibition of the ER pathway by ATRA. Taken together, the data demonstrate an anti-estrogenic action of the retinoid in primary tumors, which is in line with what was reported in breast cancer cell lines (Hua *et al*, 2009).

We performed an interactome analysis (Fig12D) looking for gene products significantly over-connected in the network modulated by ATRA in *Luminal*/ER^+^ tumors. We focused on two groups of genes relevant for the molecular mechanisms underlying the anti-tumor action of ATRA, that is, transcription factors and kinases (Supplementary Table S8). As for transcription factors, the list of over-connected genes contains *RARA*, *RARG*, and *RXRA*. The presence of ESR1 and ESR2 among the top-ranked transcription factors is in line with the process enrichment analysis described above. Finally, the inclusion of STAT1, STAT5B, and STAT3 is of relevance given the cross talk between retinoid receptors and this group of transcription factors in acute myeloid leukemias (Gianni *et al*, 1997). As for the kinases, PI3K and AKT stand out, as ATRA has been shown to inhibit the two corresponding signal transduction pathways in certain breast cancer cell lines (Paroni *et al*, 2012).

## Discussion

Exploitation of the clinical potential of ATRA requires definition of the sensitive mammary tumor subtypes and the molecular determinants of this sensitivity. In this study, we examined the responsiveness of a large panel of breast cancer cell lines, recapitulating the heterogeneity of the disease, to the anti-proliferative action of ATRA. A *Luminal* phenotype and ER expression are identified as major determinants of ATRA sensitivity. In contrast, a *Basal* phenotype, which is characteristic of *TN* tumors, is associated with ATRA refractoriness. The observations made in cell lines reflect the situation delineated in primary tumors using short-term tissue-slice cultures. We propose that ATRA should be used in a neo-adjuvant or adjuvant setting for the treatment and chemoprevention of *Luminal* ER^+^ tumors. In ER^+^ breast cancer, ATRA may represent a rational addition to anti-estrogens particularly in conditions of induced resistance to these agents (Belosay *et al*, 2006; Johansson *et al*, 2013). Despite their importance, the *Luminal*/*Basal* phenotypes and ER positivity/negativity are not sufficient determinants of ATRA sensitivity or resistance. In fact, there is a minority of the *Luminal* or ER^+^ cell lines and tumors which are refractory to the retinoid and a few *Basal* cell lines and tumors responding to ATRA. This indicates that factors other than the cell origin control the responsiveness of breast cancer cells to this anti-tumor agent.

The results obtained in short-term tissue-slice cultures demonstrate that ATRA exerts major quantitative effects on the transcriptomes not only of *Luminal*/ER^+^ tumors, but also of *TN* cancers. Thus, our transcriptomic data support the concept that the general refractoriness of *TN* tumors and the corresponding *Basal* cell lines to the anti-proliferative action of ATRA is not associated with a similar resistance to the transcriptional effects of the retinoid. In contrast, it is likely that ATRA sensitivity of *Luminal*/ER^+^ relative to *TN/Basal* tumor cells is the consequence of different transcription programs activated by the retinoid in the two cell types. The different complement of RAR isoforms and splicing variants present in *Luminal*/ER^+^ relative to *TN/Basal* tumor cells may be at the basis of these differential responses to ATRA.

The biological activity of ATRA is deemed to be mediated by the RXR/RAR and RXR/PPARβ/δ transcription factors via the distinct cytosolic binding proteins, CRABP2 and FABP5 (Shaw *et al*, 2003; Schug *et al*, 2007; Kannan-Thulasiraman *et al*, 2010). The correlative results obtained in cell lines and breast tumors indicate that expression of the RARα3 mRNA and the corresponding protein is directly associated with ATRA sensitivity, the *Luminal* phenotype, and ER positivity. The role of RARα in the anti-proliferative responses triggered by ATRA is supported by functional studies involving specific pharmacologic RAR agonists/antagonists performed in selected *Luminal* and *Basal* cell lines. RARα over-expression and knockdown experiments provide direct evidence for the involvement of the receptor not only in ATRA-dependent growth inhibition, but also in other aspects of ATRA anti-tumor activity. In addition, RARα is a biomarker of ATRA sensitivity and the major target for retinoids in breast cancer. This suggests that specific RARα agonists should be developed for the management of the disease to avoid side effects and toxicity associated with the clinical use of a pan-RAR agonist like ATRA (Garattini *et al*, 2007b, 2014).

Although RARα is an important mediator of ATRA anti-tumor activity, it is unlikely to represent the only determinant of sensitivity. The search for other genes outside the retinoid pathway performed in this study resulted in the definition of two gene sets whose basal expression levels are associated with retinoid sensitivity/resistance in our panel of cell lines. These gene sets are relevant from both a basic and an applied perspective. At the basic level, the two gene sets provide information on previously unrecognized genes and gene networks which may control/influence the sensitivity of cancer cells to ATRA. Our gene sets show a significant overlap (M. Bolis, unpublished observations) with the gene-expression signatures determined for PI3K-inhibitors (Daemen *et al*, 2013). This suggests that part of the anti-proliferative action of ATRA may involve inhibition of the PI3K pathway, which is often turned on in breast cancer cells. The contention is supported by the presence of PI3K among the over-connected kinases in the network of gene products modulated by ATRA in primary tumors challenged with ATRA *ex vivo*. With respect to this, a major link may be represented by *PREX1* (RAC-exchanger-factor-1), an important determinant of the sensitivity of breast cancer cells to PI3K inhibitors (Ebi *et al*, 2013). Interestingly, combinations of PI3K inhibitors and ATRA show additive or synergistic growth effects in selected breast cancer cell lines (MT, unpublished results). At the applied level, the two gene sets contain possible pharmacological targets for the design of therapeutic combinations based on ATRA or derived retinoids. In addition, these gene sets have the potential to be optimized in view of their use as diagnostic tools for the selection of breast cancer patients who may benefit from retinoid-based treatments.

In conclusion, this work is a first step toward a rational use of ATRA and derived retinoids in breast cancer. The data obtained with both the cell lines and the short-term tissue cultures indicate that ∽70% of *Luminal* breast cancers are likely to be responsive to ATRA. As ATRA is characterized by low toxicity as well as mild side effects, our data strongly suggest that the compound is of potential interest in the adjuvant therapy of the majority of *Luminal* breast cancer with particular reference to ER^+^ tumors. Indeed, the results obtained represent the rationale for an independent clinical trial (AZ and EG, personal communication), which we will conduct in post-menopausal patients suffering from ER^+^ breast cancer aimed at evaluating the efficacy of ATRA addition to aromatase inhibitors.

## Materials and Methods

### Chemicals plasmids and cell lines

The following compounds were used: ATRA (Sigma-Aldrich, https://www.sigmaaldrich.com), AM580 (Tocris, http://www.tocris.com), BMS961 (Tocris), ER50891 (Tocris), CD2665 (Tocris), and UVI2003 (a kind gift of Dr. Angel De Lera, Universidade de Vigo, Spain). Sulforhodamine was from Sigma-Aldrich Co. A list of the cell lines, their characteristics, and origin is available in Supplementary Table S1. The plasmid constructs used for RARα3 over-expression in *MDA-MB453* cells and knockdown in *SKBR3* cells are described below.

### Plasmid construction

To generate the RARα plasmid used for the over-expression in *MDA-MB453* cells, 5′ FLAG-tagged RARα1/3 cDNA was introduced into *pcDNA3*, using the NdeI and XhoI sites in the multiple cloning region downstream of the pCMV promoter. To obtain the RARα silencing construct, a custom-synthesized double-stranded DNA coding for a RARα-targeting shRNA (5′-GATCCGCGGGCACCTCAATGGGTACTTCCTGTCAGATACCCATTGAGGTGCCCGCTTTTTG-3′, the underlined sequence corresponds to nt 629–646 of the NM_001145301.2 sequence, Sigma-Aldrich) was introduced into the *pGreenPuro* plasmid (System Biosciences Inc., http://www.systembio.com), using the EcoRI and BamHI sites in the multiple cloning region downstream of the H1 gene promoter. The RARα over-expressing *MDA-MB453* clones and the *RARA*-silenced *SKBR3* clones were selected, after transfection with Fugene HD (Promega, www.promega.com), in the presence of 400 μg/ml G418 and 1 μg/ml puromycin (Sigma-Aldrich), respectively.

### Short-term tissue slice cultures

Tissue cultures of primary breast tumors were performed as described (van der Kuip *et al*, 2006). Briefly, tissue slices (thickness, 200 μm) deriving from surgical specimens of 45 breast cancer patients who underwent a diagnostic *Tru-cut* procedure were challenged with vehicle (DMSO) or ATRA (0.1 μM) for 48 h in Mammary Epithelial Cell Growth Medium (Lonza, Allendale, NJ). At the end of the treatment, samples were fixed, paraffin-included, and dissected into 5-μm slices, which were subjected to immuno-histochemical staining with an antibody targeting the Ki67 proliferation-associated marker. The percentage of Ki67-positive tumor cells in the various samples was assessed in a quantitative manner by automatic image analysis, and the results are illustrated. Scoring of Ki67 was blinded as to treatment. Each value represents the mean ± SE of at least five separate fields for each experimental sample. The fresh primary tumor samples used for the short-term tissue slice cultures aimed at assessing ATRA sensitivity were supplied by Fondazione S. Maugeri, Pavia. All the procedures were approved by the internal ethical committee of the Fondazione S. Maugeri, and an informed consent for the donation of the sample was obtained from patients.

### ATRA score

Cell lines were exposed to increasing concentrations of ATRA (0.001–10.0 μM) for 3, 6, and 9 day, and cell growth was determined with sulforhodamine assays (Skehan *et al*, 1990; Voigt, 2005; Vichai & Kirtikara, 2006). A detailed description of the *ATRA scores* and associated mathematical equations and models is available in Supplementary Methods.

**The paper explained**

**Problem**

All-*trans* retinoic acid (ATRA) is the primary vitamin A metabolite and a promising agent in the treatment/chemoprevention of breast cancer. Breast cancer is very heterogeneous, being a collection of different diseases. A rational use of ATRA in breast cancer requires the definition of the sensitive subtypes and the molecular determinants underlying retinoid sensitivity.

**Results**

We examined ATRA sensitivity in a large panel of breast cancer cell lines, recapitulating the heterogeneity of the disease after development of a new parameter (*ATRA score*) defining ATRA sensitivity in a quantitative manner. *Luminal* and ER^+^ (estrogen-receptor-positive) cell lines are generally sensitive to ATRA. In contrast, refractoriness or low sensitivity is associated with a *Basal* phenotype and HER2 positivity. The associations between cellular phenotype and ATRA sensitivity are confirmed using short-term tissue-slice cultures of primary tumors, which also result in the definition of the modifications in the transcriptome afforded by ATRA in *Luminal* and *Basal* tumors. Using a retinoid-pathway-oriented approach, RARα is identified as the only member of the retinoid pathway directly associated with the *Luminal* phenotype, estrogen positivity, and ATRA sensitivity. Studies in selected *Luminal* and *Basal* cell lines with RAR-specific agonists/antagonists confirm that RARα is the principal mediator of ATRA responsiveness. In addition, RARα over-expression sensitizes ATRA-resistant cells to the retinoid. In contrast, RARα silencing in ATRA-sensitive cells abrogates the activity of the retinoid. All this is paralleled by similar effects on ATRA-dependent inhibition of cell motility, indicating that RARα mediates also ATRA anti-metastatic effects. Whole-genome gene-expression data allow the definition of two overlapping gene sets expressed in basal conditions and characterized by predictive potential and associated with ATRA sensitivity in breast cancer cell lines and tumors.

**Impact**

From a basic point of view, we provide information as to the breast cancer cellular phenotypes and the RAR isoform regulating ATRA sensitivity. In addition, we identify a first list of genes of functional relevance for the anti-tumor activity of ATRA and derived retinoids in breast cancer. From an applied perspective, the study provides fundamental information for the development of retinoid-based therapeutic strategies aimed at the stratified treatment of breast cancer. Finally, the data suggest that therapeutic strategies based on the use of RARα-specific retinoids may overcome the toxicity problems associated with the clinical use of a pan-RAR agonist like ATRA.

### Xenotransplants of *HCC-1599* cells

*HCC-1599* cells (1 × 10^7^/animal) were injected subcutaneously on both flanks of female 6-week-old SCID mice weighing ∽18 g (Harlan Laboratories, http://www.harlan.com). All the experiments were performed following approval of the internal Ethical Committee on Animal Experimentation and were conducted in compliance with the Italian legislation. Tumor volume was determined with a caliper and by magnetic resonance imaging (MRI, Supplementary Methods).

### PCR and Western blot analyses

Real-time PCR was performed using Taqman assays (Terao *et al*, 2011). Amplimers and Taqman probes (Life Technologies Italia, Monza, Italy) are listed in Supplementary Methods. Western blots were performed with RARα (Gianni *et al*, 2012), β-actin, tubulin, SMAD3 (Paroni *et al*, 2012), and β-catenin (Paroni *et al*, 2012) antibodies.

### Gene-expression studies in short-term tissue cultures of primary tumors

Tissue slices were incubated with vehicle (DMSO) or ATRA (0.1 μM) for 48 h. Total RNA was extracted with the miRNeasy Mini kit (QIAGEN), labeled with the Lowinput Quick Amp labeling kit (Cy3 mono color, Agilent), and hybridized to whole-genome gene expression microarrays (Agilent). Fluorescent signals were determined and quantified with an Agilent microarray laser scanner. The microarray raw data and experimental protocols were deposited in the Arrayexpress database (accession No. E-MTAB-3313).

### Bioinformatic analysis of the gene-expression microarray and RNA-seq data

Gene expression data for the cell lines were derived from the Affymetrix GeneChip Human Genome U133 Plus 2.0 arrays (provided by CCLE, http://www.broadinstitute.org/ccle). *RAW* sequencing data (Illumina-paired end reads) were derived from two distinct datasets. The first dataset is publicly available in the CCLE project and. BAM files where downloaded through the *cgdownload* utility from the Cancer Genomics Hub (CGHub/UCSC, https://cghub.ucsc.edu). Sequencing data (.FASTQ files) for those cell lines that were not part of this first set were downloaded from a second GenBank dataset under the accession GSE48216 (GenBank). The heat-maps were generated using the algorithms available in T-Mev (http://www.tm4.org). Further details on the bioinformatic analyses performed on all the gene-expression data are available in the appropriate sections of the Supplementary Information.

## Supporting Information

Supplementary Table S8

Review Process File
