# Supplementary material for: Cellular and molecular determinants of all-trans retinoic acid sensitivity in breast cancer: Luminal phenotype and RARα expression
Source: EMBO Mol Med. 2015 Apr 17;7(7):950–72. doi: 10.15252/emmm.201404670 (PMC4520659; doi:10.15252/emmm.201404670)
Supplement: Supplementary file 1 [file emmm0007-0950-sd1.pdf]

SUPPLEMENTARY INFORMATION

**Title**: Cellular and molecular determinants of *all-trans* retinoic acid sensitivity in breast cancer

Floriana Centritto, Gabriela Paroni, Marco Bolis, Silvio Ken Garattini, Mami Kurosaki, Maria Monica Barzago, Adriana Zanetti, James Neil Fisher, Mark Francis Scott, Linda Pattini, Monica Lupi, Paolo Ubezio, Francesca Piccotti, Alberto Zambelli, Paola Rizzo, Maurizio Gianni', Maddalena Fratelli, Mineko Terao and Enrico Garattini

SUPPLEMENTARY METHODS Page: 2-9

SUPPLEMENTARY REFERENCES Page: 10

SUPPLEMENTARY TABLE AND FIGURE LEGENDS Page: 11-19

SUPPLEMENTARY FIGURES S1-S13 Page: 20-34

SUPPLEMENTARY METHODS

*Gene expression - Microarray*

Gene expression data for the cell lines were derived from the Affymetrix GeneChip Human Genome U133 Plus 2.0 arrays (provided by CCLE - http://www.broadinstitute.org/ccle). Platform annotations were updated to the latest release based on the na.34 probe set descriptions for the Affymetrix U133plus2 array (Release 34). Probes that were devoid of a gene-name association in the na.34 dataset were annotated, when possible, through *GeneAnnot* (weizmann.ac.il). The U133plus2 RAW cell-line expression data were pre-processed with the RMA algorithm in R and normalized using quantile normalization. Gene expression values were expressed in a log2 scale. The heatmaps were generated using hierarchical clustering and Pearson's uncentered algorithm as distance measure which is available in T-Mev (http://www.tm4.org).

*Gene expression – RNA-Seq*

*RAW* sequencing data (*Illumina-paired end reads*) were derived from two distinct datasets. The first dataset is publicly available in the CCLE project and .BAM files where downloaded through the *cgdownload* utility from the Cancer Genomics Hub (CGHub/UCSC-https://cghub.ucsc.edu). The .FASTQ files were subsequently reconstructed from the down-loaded .BAM files through *Bam2FastQ*script(http://genome.sph.umich.edu/wiki/BamUtil). Sequencing data (.FASTQ files) for those cell lines that were not part of this first set were down-loaded from a second GEO dataset under the accession GSE48216 (GEO). RNA-Seq data analysis was performed using the latest Gencode annotations (v19) based on human genome assembly hg19 (UCSC-Santa Cruz). The alignment of sequencing reads to the genome was performed using STAR (https://code.google.com/p/rna-star), adopting default parameters. Gene-oriented expression tables for the 40 cell lines were computed using *Cuffnorm*, which is available in the latest release of *Cufflinks v.2.2.1* (cufflinks.cbcb.umd.edu).

*Gene network enrichment analysis*

We performed enrichment analysis on the genes regulated by ATRA in Luminal tumors ($p<0.005$, paired t-test) on Metacore-annotated process networks (http://thomsonreuters.com/metacore). Each process represents a preset network of protein interactions characteristic of the process itself. Enrichment analysis is performed through the hypergeometric test and corrected for multiple testing calculating the false discovery rate (FDR). For the ESR1 (estrogen receptor 1) signaling pathway, we built a network reporting all the interactions among the genes regulated by ATRA and the most relevant neighbors.

*Clinical datasets*

Gene expression data of more than 1000 primary breast tumors were obtained from the TCGA dataset (The Cancer Genome Atlas - cancergenome.nih.gov). For this study we used level3 TCGA RNA-Seq data, which were processed and normalized (upper quartile normalization, reference genome: hg19).

*RandomForest – Ranking of features*

The Random Forest (*Breiman, Leo (2001). "Random Forests"*) algorithm was used to identify features (genes) associated with ATRA response. Random Forests regression was performed using the R 'Random Forest' library with response as the ATRA-sensitivity score. 30000+1 trees were grown (odd number ensures a fully deterministic model) using default settings (mtry=n/3). The aim of the current work was not to create a predictive fingerprint, but rather to generate a ranked list of genes potentially associated with ATRA-sensitivity by comparing multiple platforms (Microarray/RNA-seq). We computed rankings for both micro-array and RNA-seq data separately, and determined a combined rank based on the geometric mean of the variable importance for those genes that were represented in both technologies. All available cell lines were used to compute feature-importance and ranked gene lists were produced for both RNA-seq and

microarray data. Microarray data were pre-processed mainly because of the background noise that is intrinsic to this technology. Preliminary unsupervised filters based on variance and signal detection above background were applied to the CCLE-microarray dataset. The Coefficient of Variation (defined as $CV = \sigma/\mu$) was calculated for each probe-set. A conservative variance filter was applied, discarding probes that had a Coefficient of Variation (CV) <**0.2**. Probe-sets that showed an expression value below log2(15) = 3.9 in 80 percent of the cell lines were discarded regardless of their Coefficient of Variation. Additionally, array-control probe-sets were removed (probe name prefix "AFFX-"). Multiple probe-sets pointing to the same gene symbol were allowed because they do not necessarily target the same transcript or isoform.

*Taqman assays*

The amplimers and Taqman probes used for the reverse-transcriptase RT-PCR assays of the following transcripts were custom synthesized by Life Technologies Italia (Monza, Italy): RARα1 (NM_000964- forward primer = 5'-GGAATCCTGAATCGAGCTGAGA-3', nucleotides 36-57; reverse primer = 5'-CAGTTCTGTGAGCTGGCACTTT-3', nucleotides 236-215; Taqman probe = 5'- AGCAGCATCACAGGACAT-3', nucleotides 112-129); RARα4 (NM_001145302- forward primer = 5'-GCACCAGCTTCCAGTTAGTGGAT-3', nucleotides 725-747; reverse primer = 5'-CTTGTTTCGGTCGTTTCTCACA-3', nucleotides 791-770; Taqman probe = 5'-CACACCATCCCCAGCCA -3', nucleotides 752-768); RARγ3 (NM_001243732-forward primer = 5'-GCCGGACTTGAATCTTTTGC-3', nucleotides 419-438; reverse primer = 5'-GGATGCTTCGGCGAAAGAA-3', nucleotides 486-468; Taqman probe = 5'-CGCCAGCCTGCAAT-3', nucleotides 450-464). The inventoried Taqman assays for the indicated transcripts were purchased from Life Technologies Italia: RARα2 (Hs00940453_m1), RARα3 (Hs00940455_m1), RARβ1 (Hs00233405_m1), RARβ2 (Hs00977143_m1), RARγ1 (Hs00171273_m1), RARγ2 (Hs00233337_m1), CYP26A1 (Hs00175627_m1), CYP26B1 (Hs01011223_m1); RARRES3 (Hs01058986_m1), BTG2 (Hs00198887_m1), 18S

(Hs99999901_s1), B2M (Hs99999907_m1), β-actin (Hs01060665_g1), CNTNAP3 (Hs01674935_m1), CDC14B (Hs00372920_m1), VAMP3 (Hs00922166_m1), PARP4 (Hs00173105_m1), THRA (Hs00268470_m1), NEBL (Hs01590594_m1), EEA1 (Hs00929215_m1), CXXC5 (Hs00212840_m1), BRD4 (Hs04188087_m1), STAT5B (Hs00273500_m1), PPP2RIB (Hs00988483_m1), PBX1 (Hs00231228_m1), MEIS2 (Hs00542638_m1), TNFRSF10B (Hs00366278_m1), COPS7B (Hs00224139_m1), DDX46 (Hs01557619_m1), RBM45 (Hs00396493-m1).

*Magnetic Resonance Imaging (MRI)*

Animals were anesthetized with isoflurane in a mixture of $O_2$ (30%) and $N_2O$ (70%). Body temperature was maintained at ~37°C by a warm water circulated heating cradle. Imaging was performed on a 7T small bore animal Scanner (Bruker Biospec, Ettlingen, Germany). Two actively decoupled radio frequency coils were used: a volume coil of 7.2 cm diameter used as the transmitter and a 2x2 array surface coil as the receiver. A 2D RARE (rapid acquisition with relaxation enhancement) T2-weighted sequence triggered by respiration was performed to assess tumor volume quantification. The morphological images were obtained with an in-plane resolution of 117x78 µm (matrix = 298x256 and Field of View = 3.5x2.0 cm), slice thickness = 500 µm; TR = 4000 ms, effective TE = 38 ms and a RARE factor of 8, for 2 averages. The volume measurements of structural MRI images were obtained manually using a custom-made software. The tumor boundaries were manually chosen and drawn on the images for volumetric assessment. Data from each animal were obtained by the integration of averaged ROI area for slice thickness.

*Determination of the ATRA-score*

Many colorimetric/fluorimetric assays are available for testing the anti-proliferative properties of drugs *in-vitro*. The output of all these assays are values (e.g. absorbance) associated with the number of cells in a well/plate at a specific time. Typically, the assays are used to assess

the response to a number of drug concentrations and they measure the concentration-response of the output-signal ratios for treated over control samples (%T/C) defining the drug concentration producing 50% inhibition ($IC_{50}$). The resulting values are heavily dependent not only on the arbitrary sampling time-point, but also on the growth kinetics of an untreated reference control (determining the T/C denominator). Thus the standard methods adopted to calculate the $IC_{50}$ are not suitable to compare the effects of a drug in different cell lines characterized by different growth kinetics. To obtain an $IC_{50}$ value taking into account the growth characteristics of the control cells, two time points for the measurement are often considered. Usually, though not necessarily, one of the time points is represented by the start of the treatment (time zero). The data are subsequently analyzed with two different formulae that describe two distinct cases: 1) the drug reduces cell-proliferation, yet an increase in total cell number relative to time zero is observed; 2) the total cell number decreases. In the NCI protocol (http://dtp.nci.nih.gov/branches/btb/ivclsp.html, Shoemaker 2006), the percent growth-inhibition (*%GI*) is calculated according to the equations: a) *%GI* = [(Ti-Tz)/(C-Tz)] x 100, for concentrations at which Ti≥Tz; b) *%GI* = [(Ti-Tz)/Tz] x 100, for concentrations at which Ti<Tz. In the two equations, C and Ti are the cell number-related signals of controls and treated samples with a concentration “i”, while Tz is the signal at time zero. It is noticeable that the first equation measures the residual fraction of the growth unaffected by treatment, so that the real proportion of affected or “inhibited” cells is 100 - *%GI*. For instance, *GI25* is a concentration producing a *%GI* = 75%. Only when there is a net loss of cells following treatment (i.e. Ti<Tz) is the presence of a cytotoxic effect proved, otherwise a pure cytostatic effect cannot be excluded. Clearly, cytostatic and cytotoxic effects are often contemporarily present, the former prevailing at low concentrations and the latter at high concentrations. In principle, it should be possible to deconvolute the complexity of the response to treatment by separating the cytotoxic from the cytostatic effects *via* measurement of the differential perturbations in the cell-cycle phases and the time-dependence of the response at each cell cycle checkpoint. However this kind of

analysis requires both an experimental and computational effort that make the approach unsuitable for screening purposes (Lupi et al. 2004, Ubezio et al. 2009, Falcetta et al. 2013).

To compare the efficacy of several drugs in a panel of 60 cancer cell-lines after 72 hours of continuous treatment, the NCI selected three concentrations obtained from each concentration-response outline: *GI50* (producing 50% GI), *TGI* (total-growth-inhibition for which Ti=Tz) and *LC50* (causing 50% reduction in Ti compared to Tz) indicating a net loss of cells following treatment. In practice, a single score, usually the *GI50*, is used to compare drug efficacy across cell lines, the higher is the score the less effective is the drug. However, the *GI50* value may be imprecise when the descent of the concentration-response curve is outside the range of the tested concentrations. In particular, *GI50* may exceed the highest concentration of the test drug in the case of very resistant cell-lines. Reciprocally, the value may be lower than the lowest tested concentration in the case of very sensitive cell-lines. In both cases, a defined *GI50* cannot be calculated without the adoption of unreliable extrapolations outside the data range. In such circumstances, it may be useful to reverse the perspective on the concentration-response curve, i.e. comparing the *%GI* at the same concentration instead of comparing the concentrations at the same inhibitory level. In this case, however, when considering a single concentration, the differences across cell-lines flatten at the extremes, so that "sensitive" cell-lines with similarly low *%GI* may have very different *GI50*, slightly or substantially below the test concentration. Conversely, "resistant" cell lines with *%GI* near 100 may have a *GI50* slightly or substantially above the test concentration.

Given all the above considerations and the specificity of the molecular mechanisms underlying the anti-tumor activity of ATRA, we deemed it necessary to have a score that recapitulates the characteristics of a concentration-response curve with a single number to overcome the problems associated with the determination of a single growth-inhibition point or a single drug concentration point. The requirement prompted us to develop a new score to profile the sensitivity of our cell-line panel to ATRA. To this purpose, we designed a score (*ATRA-score*)

which is calculated from four concentrations of the ATRA concentration-response curves: *GI25*, *GI50*, *GI75* and *TGI*. The *ATRA-score* is based on the growth-inhibitory activity of the retinoid determined with the colorimetric sulforhodamine assay (Skehan et al. 1990). In the experimental protocol used in the present study, each cell line was exposed to five concentrations of ATRA: 0.001, 0.01, 0.1, 1 and 10 μM. As the doubling time of the cell lines was variable (3.5±2.8 days, range 1 to 9 days), the use of a score taking into account this variability was mandatory. For this reason and given the slow kinetics of the response to ATRA we chose to consider the cell-number related sulforhodamine absorbance value at day 3 and day 6. We named $T_i(6)$, C(6), the absorbance at day 6 of treated (with a drug concentration "i") and control samples respectively. C(3) is the absorbance of controls at day 3. The *%GI* induced by ATRA concentration "i" was calculated, only for concentrations for which $T_i(6) \geq C(3)$, according to the formula: *%GI*(i) = 100 x [$T_i(6)$-$T_i(3)$] / [C(6)-C(3)]. The %GI(i) was fitted with a Hill function and the *GI25*, *GI50* and *GI75* ATRA concentrations were calculated from the best fit function, if at least three data points with *%GI* between 5 and 95% could be fitted and the $R^2$ of the fit was >0.8. Otherwise a linear interpolation approach was applied to calculate the concentrations. The *TGI* was calculated by linear interpolation of the absorbance values between the two subsequent concentrations for which $T_i(6) \geq T_i(3)$and $T_{i+1}(6) < T_{i+1}(3)$. In case $T_{10\mu M}(6) > T_{10\mu M}(3)$, we set TGI>10 μM.

A resistance score (*ReSc*) for each cell line was calculated in two steps. In the first step, the cell line is assigned to one of five categories considering the response to the highest ATRA concentration (10 μM), according to the following rules: **category 1**: TGI ≤ 10μM; **category 2**: GI75 ≤ 10μM and TGI > 10μM; **category 3** : GI50 ≤ 10μM and GI75 > 10μM; **category 4**: GI25 ≤ 10μM and GI50 > 10μM; **category 5**: GI25 >10μM. Thus, the most sensitive cell lines are grouped in **category 1** and the most resistant ones in **category 5**. The category grossly identifies the location of the concentration-response curve relative to a reference concentration (10 μM). A second step is required to set the position of the curve within each category precisely. This is obtained with the quantity more appropriate for each category (either TGI or GIx). ReSc is

calculated as the sum of two contributions, a category penalty A and an intra-category score B, according to the following table:

*Resistance Score formulae (ReSc=**A**+**B**)*

| | **A** | **B** |
|---|---|---|
| **Category 1** | 0 | +log10(TGI)+1 |
| **Category 2** | 5 | +log10(GI75)+1 |
| **Category 3** | 10 | +log10(GI50)+1 |
| **Category 4** | 15 | +log10(GI25)+1 |
| **Category 5** | 20 | +($\%GI_{10\mu M}$-75)/25 |

The intra-category score B (0<B<5) enabled us to obtain a continuous score, ranking cell lines within a category, according to the value of *TG1* (**category 1**), *GI75* (**category 2**), *GI50* (**category 3**), *GI25* (**category 4**). In the case of **category 5**, as even the *GI25* was higher than 10μM, the B score was calculated on the basis of the $\%GI_{10\mu M}$ value. This value was close to 0 when $\%G_{10\mu M}$ was slightly higher than 75%, 1 when *%GI* =100% . Values higher than 1 were allowed when $T_{10\mu M}(6)>C(6)$ and cell growth was enhanced by treatment. Finally, to define a score increasing with the efficacy of ATRA, the resistance score was reversed and scaled in a range between 0 and 1 (between *ReSc-max* and ReSc-min, the maximum and minimum *ReSc* values in our set of cell lines), using the “rescale” function (package plotrix) in R, to obtain the final *ATRA-score* according to the formula: *ATRA-score* = (*ReSc-max* – *ReSc*)/(*ReSc-max* – *ReSc-min*).

SUPPLEMENTARY REFERENCES

-Falcetta F., Lupi M., Colombo V., Ubezio P. (2013) Dynamic Rendering of the Heterogeneous Cell Response to Anticancer Treatments. PLoSComput Biol. 2013 Oct;9(10):e1003293.

Shoemaker, R. H. The NCI60 Human Tumour Cell line Anticancer Drug Screen. Nature Reviews, 6: 813-823, 2006.

- Gianni M, Peviani M, Bruck N, Rambaldi A, Borleri G, Terao M, Kurosaki M, Paroni G, Rochette-Egly C, Garattini E. (2012) p38αMAPK interacts with and inhibits RARα: suppression of the kinase enhances the therapeutic activity of retinoids in acute myeloid leukemia cells. Leukemia 26: 1850-1861.

-Lupi, M., Matera, G., Branduardi, D., D'Incalci M. and Ubezio, P. (2004) Cytostatic and cytotoxic effects of topotecan decoded by a novel mathematical simulation approach. Cancer Res. 64: 2825-2832.

-Ubezio, P.,Lupi, M., Branduardi, D.; Cappella, P., Cavallini, E., Colombo, V., Matera, G., Natoli, C., Tomasoni, D., D'Incalci, M. (2009) Quantitative assessment of the complex dynamics of G1, S and G2M checkpoint activities. Cancer Res. 69: 5234-5240.

-Skehan, P., Storeng, R., Scudiero D. *et al*. (1990) New colorimetric cytotoxicity assay for anti-cancer-drug screening. J Natl Cancer Inst. 82: 1107-1112.

SUPPLEMENTARY TABLE AND FIGURE LEGENDS

**Suppl. Table S1** *Characteristics and origin of the breast cancer cell lines*

The table lists the characteristics and the origin of the panel of breast cancer cell lines used in the study. The cell lines were obtained directly from the ATCC (American Type Culture Collection, http://www.atcc.org) and the DSMZ GmbH (Deutsche Samlung Von Mikroorganismen und Zellkulturen, http://www.dsmz.de) repositories. All the cell lines were grown in optimal culture conditions according to the instructions of the two providers.

**Suppl. Table S2** *ATRA-score, growth-inhibition associated indexes and duplication time of the CCLE panel of breast cancer cell lines*

The table contains a characterization of the breast cancer cell lines used in the study for their response to ATRA. The terms, CATEGORY, GI25, GI50, GI50, GI75, TGI and %GI10µM refer to the parameters described in Supplementary Methods which were used for the calculation of the *ATRA-score*. nd = not determinable.

**Suppl. Table S3** *Primary tumors used for the short-term tissue slice cultures: patient characteristics*

The table lists the characteristics of the patients whose primary surgical samples were used for the short-term tissue slice cultures. ER-positivity = estrogen receptor positivity; PR-positivity = progesterone receptor positivity. The amplification of the RARA locus was measured by quantitative real-time PCR using the DNA extracted from the primary tumors (Paroni, G *et al.* 2011).

**Suppl. Table S4** *List of ATRA-sensitivity associated genes*

The table lists all the 57,797 genes that were used for the random forest analysis (RNASeq data). The genes are ranked based on their variable importance value, which is expressed in percent increase in mean squared error (% inc MSE). A second Random Forest model was trained for microarrays probes and variable importance metrics were computed in the same way. Not all the genes in the list have a counterpart in the Affymetrix platform, but for each gene we annotated the corresponding microarray probe, if this was available. When multiple probes were targeting the same gene, we selected the one that had the highest variable importance metrics. The annotated probes were then ranked based on their percent increase in mean squared error (% inc MSE). Additionally, for those genes that had a match in both platforms, we computed a third rank which is equal to the geometric mean (RANK_GEOMEAN) of the previously defined rankings (RNA-Seq/Microarray). Furthermore, for those genes that were cross-validated using RT-PCR, we provide the Pearson's correlation coefficient (r) and its squared value (r^2) based on the correlation between PCR and RNA-Seq expression values.

**Suppl. Table S5** *Transcriptome perturbations afforded by ATRA in short-term primary tumor cultures*

The table contains the list of genes which were significantly altered by ATRA in either *Luminal* or *TN* tumors ($p<0.005$, paired t-test). The data are expressed as the log2 ratio of gene expression in ATRA vs. vehicle treated samples. The p-values of the following comparisons are reported: ATRA vs. vehicle in *TN* tumors; ATRA vs. vehicle in *Luminal* tumors; $\log_2$ ratio in *TN* vs *Luminal* tumors.

**Suppl. Table S6** *Process network enrichment analysis of the genes regulated by ATRA in Luminal tumors*

The table lists the top-10 rankings of the enriched process networks. The table reports the p-value of the hypergeometric test, the FDR (false discovery rate), the number and the list of objects in the networks.

**Suppl. Table S7** *Interactions of the process network "ESR1nuclear signaling pathway" in Luminal tumors*

The table lists all the interactions depicted in Fig. 12D and describes the type of these interactions with relevant references. The last four columns show the significant changes in the expression levels of the network nodes afforded by ATRA.

**Suppl. Table S8** *Interactome analysis*

The tables lists the top-ranked transcription factors and protein kinases identified by interactome analysis with Metacore. The "IDs in active dataset" column lists the gene-symbols of the over-connected objects present in the list of genes regulated by ATRA in primary breast tumor samples (active dataset, Fig. 12A). the "Object Name" column contains the gene-symbol of the over-connected object. The "Actual" column describes the number of connections of the specified object with the active dataset. The "n" column describes the total number of objects in the active dataset. "R" is the total number of connections the specified object has with the entire database. "N" is the total number of objects in the database. The "Expected" column describes the number of connections that the listed object is expected to have with the active dataset purely by chance. The "Ratio" column contains the number of actual connections of the specified object with the active dataset divided by the expected number of connections. The "p-value" describes the probability of the observed ratio of connections with the specified object occurring purely by chance. The "Z-score" gives a measure of how saturated is the total number of connections that the object has with

the objects of the active dataset. The higher is the Z-score the more saturated is the number of the object's connections.

**Suppl. Fig. S1** *Classification of the CCLE panel of breast cancer cell lines according to the whole-genome gene-expression profiles*

The figure shows a dendrogram clustering all the breast cancer cell lines belonging to the Broad Institute Cancer Cell Line Encyclopedia (CCLE) database according to the PAM50 gene-expression fingerprint determined under basal conditions. The two main arms of the cluster distinguish the cell lines according to the luminal and basal phenotypes. The cell lines used in this study are indicated in black, while those which were not considered are marked in grey.

**Suppl. Fig. S2** *Body weight change in animals bearing HCC-1599 cell derived tumors*

Immuno-deficient SCID mice were xenografted subcutaneously with $10x10^6$ *HCC-1599* cells on both sides. One week after transplantation 10 animals/experimental group were treated intra-peritoneally with vehicle (DMSO) or two doses of ATRA (7.5 mg/kg and 15.0 mg/Kg) once/day, 5 days a week for a total of 24 days, as indicated. At the end of this period, treatment was discontinued until sacrifice. The body weight was determined on the indicated days. Each point is the mean±SE of 10 animals.

**Suppl. Fig. S3** *Associations between components of the retinoid signaling pathway and ATRA-sensitivity*

We analyzed the gene-expression microarray and RNA-seq data associated with 40 of the breast cancer cell lines, respectively. Using a gene-oriented approach, we evaluated associations between any of the indicated retinoid receptors/binding proteins and the cellular phenotype as well as ATRA-sensitivity. For this analysis, we considered the basal expression levels of the transcripts after stratification of the cell lines for the indicated parameter. The panels show only the genes for

which no statistically significant association was determined and complements the data illustrated in Fig. 3. Cell lines were ranked according to the *ATRA-score* and subdivided in ascending tertiles (T1-T3). The box plots demonstrate that the indicated transcripts show no significant difference in the expression levels in the tertile of cell lines characterized by the highest *ATRA-scores* (T1) relative to the tertile showing the lowest *ATRA-scores* (T3). Lack of significant differences is observed in the total (left), in the luminal (center) and in the basal (right) pool of cells, following analysis of either the microarray and the RNA-seq data. The number of cell lines in the T1 and T3 groups of cells considered is detailed in the Legend to Fig. 3. Abbreviations: fpkm = fragments per kilobase of exon per million fragments mapped.

**Suppl. Fig. S4** *Structure of the RAR/RXR genes and relative mRNA/protein products*

The exonic structure of the genes coding for the human RARA, RARB, RARG, RXRA, RXRB and RXRG genes along with the corresponding chromosomal location are indicated. The black boxes indicate the coding exons from exon 2 to exon 7. Below each gene the structure of the corresponding transcript variants is shown on the left side. The first ATG start-codon and the stop-codon are indicated with a vertical red line. The structure of the encoded proteins from the NH- to the COOH teriminus (left to right) are indicated on the right. Boxes drawn in different colors represent the known structural domains of the various receptors. The accession number of each mRNA variant and protein product is indicated in parenthesis.

**Suppl. Fig. S5** *RARα1/RARα2/RARα3/RARα4 mRNA expression in breast cancer cell lines*

Total RNA was extracted from the indicated cell lines during the logarithmic phase of the growth. Amplification of the *RARα1*, *RARα2*, *RARα3* and *RARα4* mRNAs was performed with specific Taqman assays and normalized for the expression of the constitutive control B2M mRNA. (**A**) The quantitative results obtained are plotted against the *ATRA-score*. The ATRA-sensitivity A-D groups and the ascending tertiles of sensitivity to the retinoid (T1-T3) are indicated. (**B**) The plots

show the correlation curves between the levels of the *RARα1*, *RARα2*, *RARα3* and *RARα4* variant transcripts. The grey area around the correlation line indicates the 90% confidence interval.

**Suppl. Fig. S6** *RARβ1 and RARβ2 mRNA expression in breast cancer cell lines*

Total RNA was extracted from the indicated cell lines during the logarithmical phase of the growth. Amplification of the *RARβ1* and *RARβ2* mRNA was performed with specific Taqman assays and normalized for the expression of the constitutive control B2M mRNA. (**A**) The quantitative results obtained are plotted against the *ATRA-score*. The ATRA-sensitivity A-D groups and the ascending tertiles of sensitivity to the retinoid (T1-T3) are indicated. (**B**) The plot shows the correlation curves between the levels of the *RARβ1* and *RARβ2* variant transcripts. The grey area around the correlation line indicates the 90% confidence interval. (**C**) The bar graphs indicate the expression levels of the *RARβ1* and *RARβ2* mRNAs in luminal *vs.* basal cells (upper panels), $ER^+$ *vs.* $ER^-$ or luminal $ER^-$ cells (middle panels) and $HER2^+$ *vs.* $HER2^-$ cells before and after stratification for the luminal phenotype (lower panels). *Significantly different (p-value < 0.05, Student's t-test). ** Significantly different (p-value < 0.01, Student's t-test).

**Suppl. Fig. S7** *RARγ1/RARγ2/RARγ3 mRNA expression in breast cancer cell lines*

Total RNA was extracted from the indicated cell lines during the logarithmic phase of the growth. Amplification of the *RARγ1*, *RARγ2* and *RARγ3* mRNAs was performed with specific Taqman assays and normalized for the expression of the constitutive control B2M mRNA. (**A**) The quantitative results obtained are plotted against the *ATRA-score*. The ATRA-sensitivity A-D groups and the ascending tertiles of sensitivity to the retinoid (T1-T3) are indicated. (**B**) The plots show the correlation curves between the levels of the *RARγ1*, *RARγ2* and *RARγ3* variant transcripts. The grey area around the correlation line indicates the 90% confidence interval. (**C**) The bar graphs indicate the expression levels of the *RARγ1*, *RARγ2* and *RARγ3* mRNAs in luminal *vs.* basal cells (upper panels), $ER^+$ *vs.* $ER^-$ or luminal $ER^-$ cells (middle panels) and $HER2^+$ *vs.* $HER2^-$ cells

before and after stratification for the luminal phenotype (lower panels). *Significantly different (p-value < 0.05, Student's t-test). ** Significantly different (p-value < 0.01, Student's t-test).

**Suppl. Fig. S8** *Correlations between RAR variant transcripts in primary mammary tumors*

The plots show the correlation curves between the levels of the indicated RAR variant transcripts in the tumor samples. The $R^2$ correlation values of each plot are indicated. The grey area around the correlation line indicates the 90% confidence interval.

**Suppl. Fig. S9** *Selectivity and specificity of the RARα, RARβ and RARγ agonists*

*COS-7* cells were co-transfected with the indicated RAR-expressing plasmid and retinoid-responsive reporter construct (*DR5-RARE-Luc* and *β2-RARE-Luc*). Twenty four hours following transfection, cells were treated with 10 nM of the pan-RAR agonist, ATRA, the RARα agonist, AM580, the RARβ agonist, UVI2003, and the RARγ agonist, BM691, for a further 24 hours. The levels of luciferase activity were measured using a standard assay (Gianni' M. et al., 2012). The induction level of luciferase activity following 24-hours treatment with the four retinoids relative to vehicle-treated (DMSO) cells were determined. The data are expressed as the % induction of luciferase activity relative to the fold-induction value determined following treatment with ATRA. The results demonstrate the selectivity of the three agonists for the target RAR isoform. Only BMS961 shows the same activation of both RARβ and RARγ if the *β2-RARE-Luc* reporter is used.

**Suppl. Fig. S10** *Selectivity and specificity of the RARα and RARβ/RARγ antagonists*

*COS-7* cells were co-transfected with the indicated RAR-expressing plasmid and retinoid-responsive reporter construct (*DR5-RARE-Luc* and *β2-RARE-Luc*). Twenty four hours following transfection, cells were treated with vehicle (DMSO) or 100 nM of the pan-RAR agonist, ATRA, in the absence and presence of the RARα antagonist, ER50891 (3 µM), or the RARβ/γ antagonist,

CD2665 (3 μM), for a further 24 hours. The levels of luciferase activity were measured as in Suppl. Fig. 11. Each value is the mean±SD of two replicates. The results demonstrate the selectivity of the three antagonists for the target RAR isoform.

**Suppl. Fig. S11** *Effect of the RARα and RARβ/RARγ antagonists on ATRA-dependent growth inhibition in HCC-1428 and SKBR3 cells*

*HCC-1428* and *SKBR3* cells were challenged with vehicle or ATRA (100 nM), in the absence or presence of the RARα antagonist, ER50891 (3 μM), or the RARβ/γ antagonist, CD2665 (3 μM) for the indicated amount of time. The number of cells in the culture wells was calculated by the sulforhodamine assay. OD = optical density at 540 nm. Each result is the mean±SD of 5 replicate wells. ** Significantly lower than the corresponding vehicle treated controls (p-value < 0.01, Student's t-test).

**Suppl. Fig. S12** *ATRA effects on the expression of retinoid-dependent genes in RARα over-expressing and knock-down breast cancer cell lines*

The indicated RARα over-expressing and knock-down cell clones as well as the corresponding negative control cell lines were treated with ATRA (100 nM) for 24 hours. (**A**) Total RNA was extracted from the tissue slides corresponding to the indicated samples. The RNA was subjected to RT-PCR analysis following reverse transcription to determine the effects of ATRA treatment on the expression of the indicated direct retinoid target genes. Each value represents the mean±SD of 3 replicate cultures. (**B**) Cell extracts from 3 pooled cell cultures were subjected to Western blot analysis for the indicated proteins. Actin was used as a loading control.

**Suppl. Fig. S13** *Strategy used for the definition of the gene-expression fingerprint associated with ATRA-sensitivity in breast cancer cell lines*

Flow chart of the procedure used for the definition of the relative importance of the genes represented in the microarray and RNA-seq databases for the sensitivity of the breast cancer cell lines, using a Random Forest approach. The flow chart summarizes the approach used and detailed in the Supplementary Methods section. The approach resulted in the definition of the gene-expression fingerprints shown in Fig. 7 and the gene rankings described in Suppl. Table S5.

**Suppl. Fig. S14** *PCR validation of the basal gene expression data present in the microarray and RNA-seq databases associated with breast cancer cell lines*

Total RNA extracted from each of the breast cancer cell lines used in the study was subjected to real time PCR amplification of the indicated mRNAs (gene symbols on the right of the plots) using specific Taqman assays, following reverse transcription. The plots show the correlation curves between the levels of the indicated transcripts determined by PCR analysis and the corresponding expression values calculated from the microarray and RNA-seq datasets. Abbreviations: fpkm = fragments per kilobase of exon per million fragments mapped.

**Suppl. Fig. S15** *PCR validation of the basal and ATRA-modulated gene expression data determined in short-term tissue cultures of primary breast tumors*

Short-term tissue cultures from the indicated patients (see number above each dot) were treated with vehicle (DMSO) or ATRA (0.1 mM) for 48 hours. Following reverse transcription, RNA extracted from each sample was subjected to real time PCR amplification using Taqman assays specific to the indicated transcripts. The plots show the correlation curves between the levels of the indicated transcripts determined by PCR analysis and the corresponding expression values calculated from the microarray data. The grey area around the correlation line indicates the 90% confidence interval.

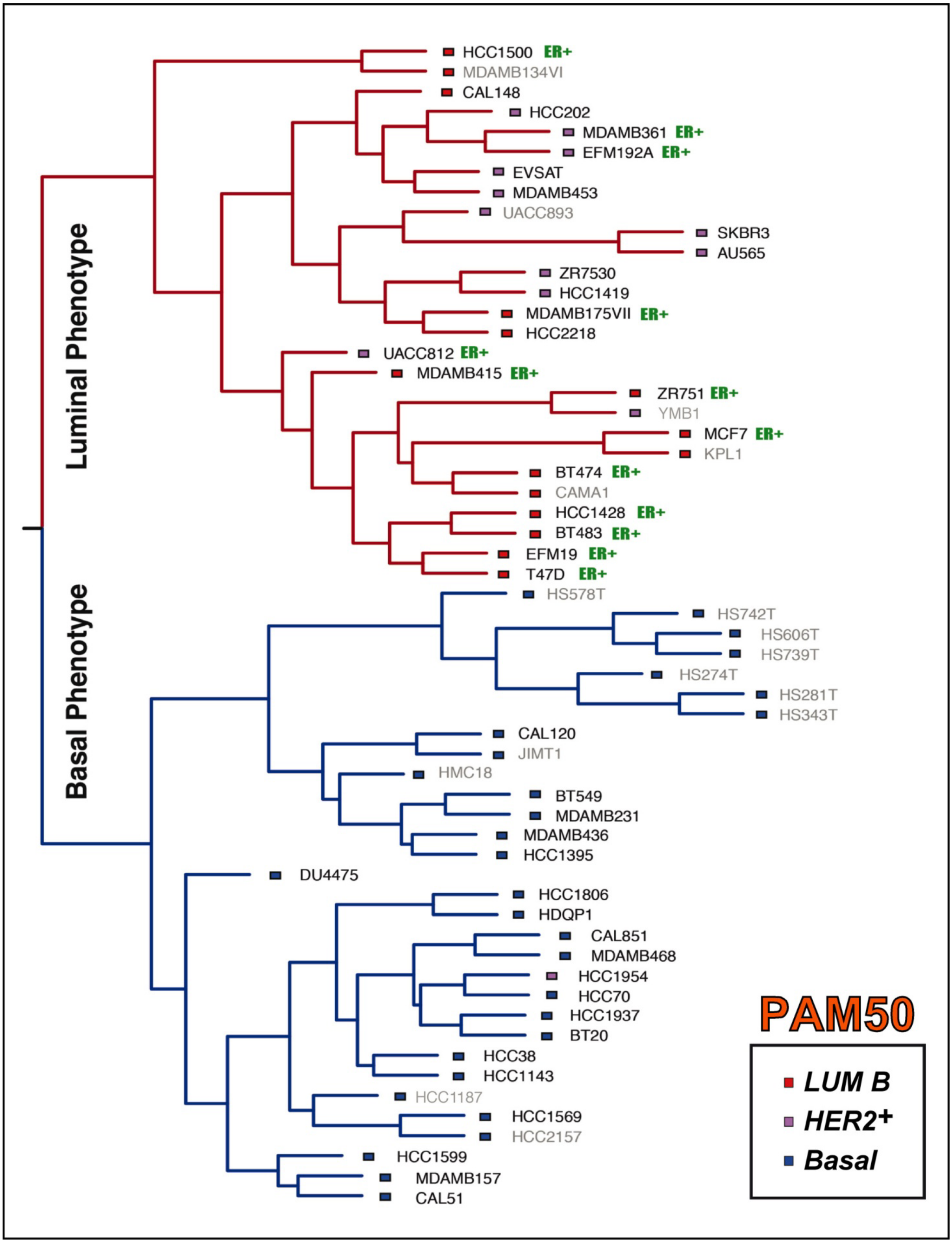

Luminal Phenotype
Basal Phenotype
HCC1500 ER+
MDAMB134VI
CAL148
HCC202
MDAMB361 ER+
EFM192A ER+
EVSAT
MDAMB453
UACC893
SKBR3
AU565
ZR7530
HCC1419
MDAMB175VII ER+
HCC2218
UACC812 ER+
MDAMB415 ER+
ZR751 ER+
YMB1
MCF7 ER+
KPL1
BT474 ER+
CAMA1
HCC1428 ER+
BT483 ER+
EFM19 ER+
T47D ER+
HS578T
HS742T
HS606T
HS739T
HS274T
HS281T
HS343T
CAL120
JIMT1
HMC18
BT549
MDAMB231
MDAMB436
HCC1395
DU4475
HCC1806
HDQP1
CAL851
MDAMB468
HCC1954
HCC70
HCC1937
BT20
HCC38
HCC1143
HCC1187
HCC1569
HCC2157
HCC1599
MDAMB157
CAL51
PAM50
LUM B
HER2+
Basal


**Suppl. Fig. S1**

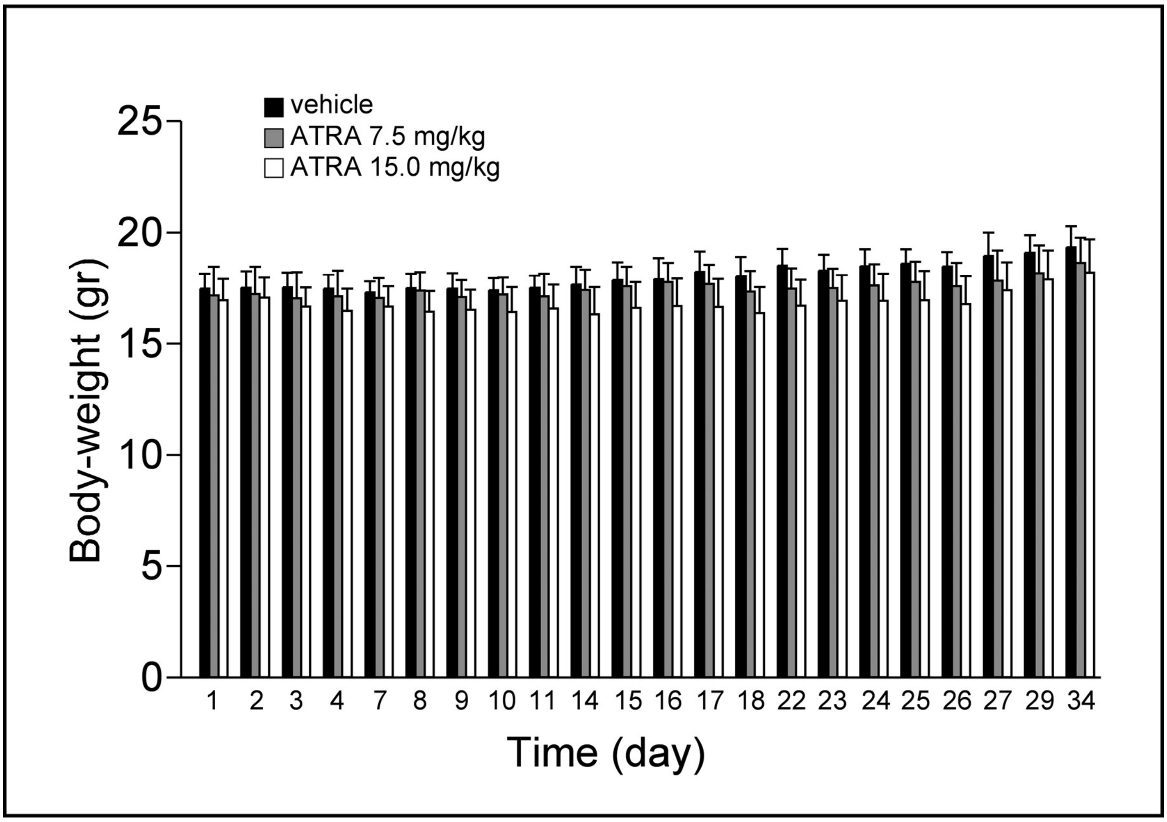
vehicle
ATRA 7.5 mg/kg
ATRA 15.0 mg/kg
Body-weight (gr)
25
20
15
10
5
0
1 2 3 4 7 8 9 10 11 14 15 16 17 18 22 23 24 25 26 27 29 34
Time (day)

**Suppl. Fig. S2**

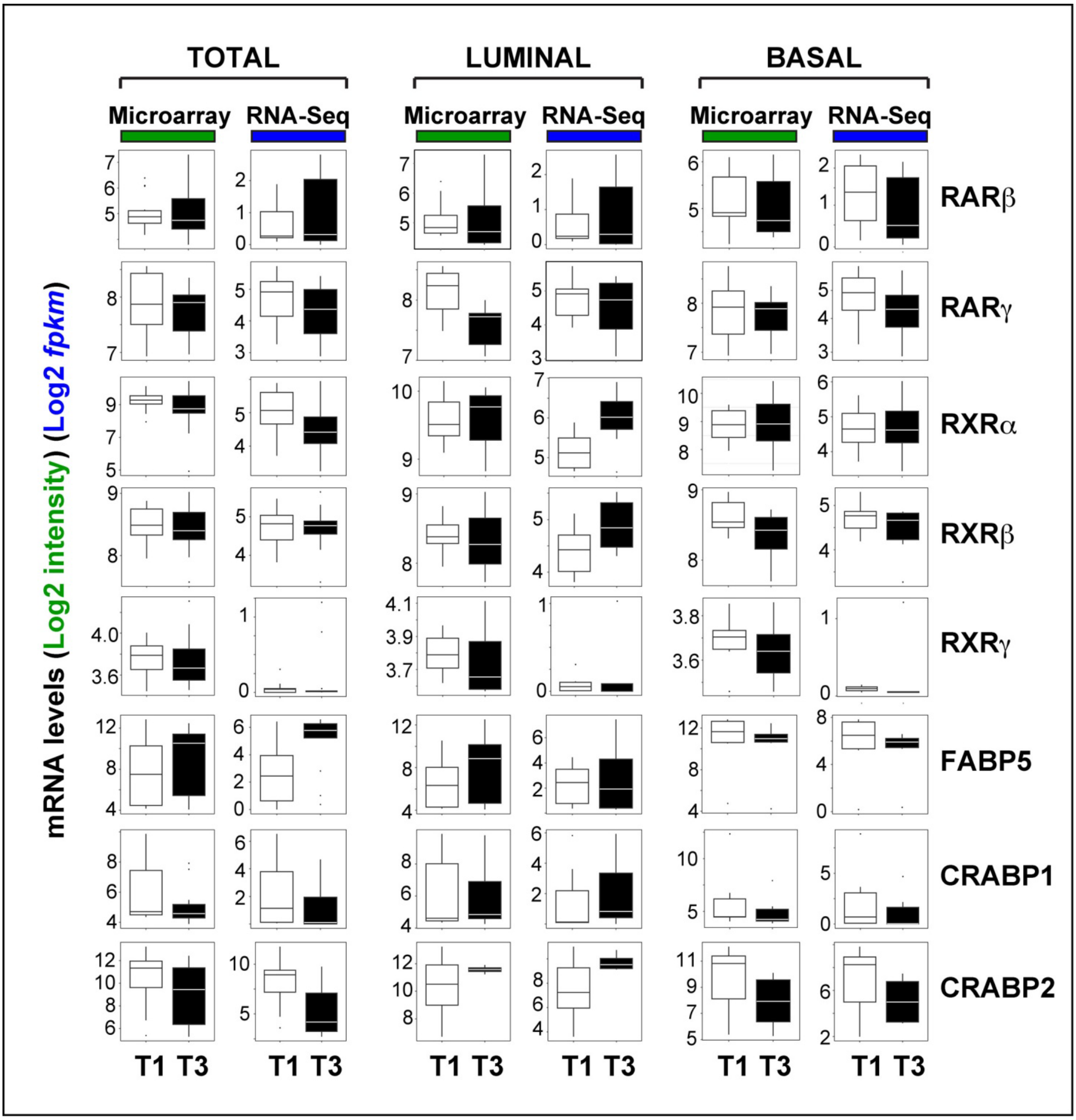

TOTAL
LUMINAL
BASAL
Microarray
RNA-Seq
mRNA levels (Log2 intensity) (Log2 fpkm)
RARβ
RARγ
RXRα
RXRβ
RXRγ
FABP5
CRABP1
CRABP2
T1 T3


**Suppl. Fig. S3**

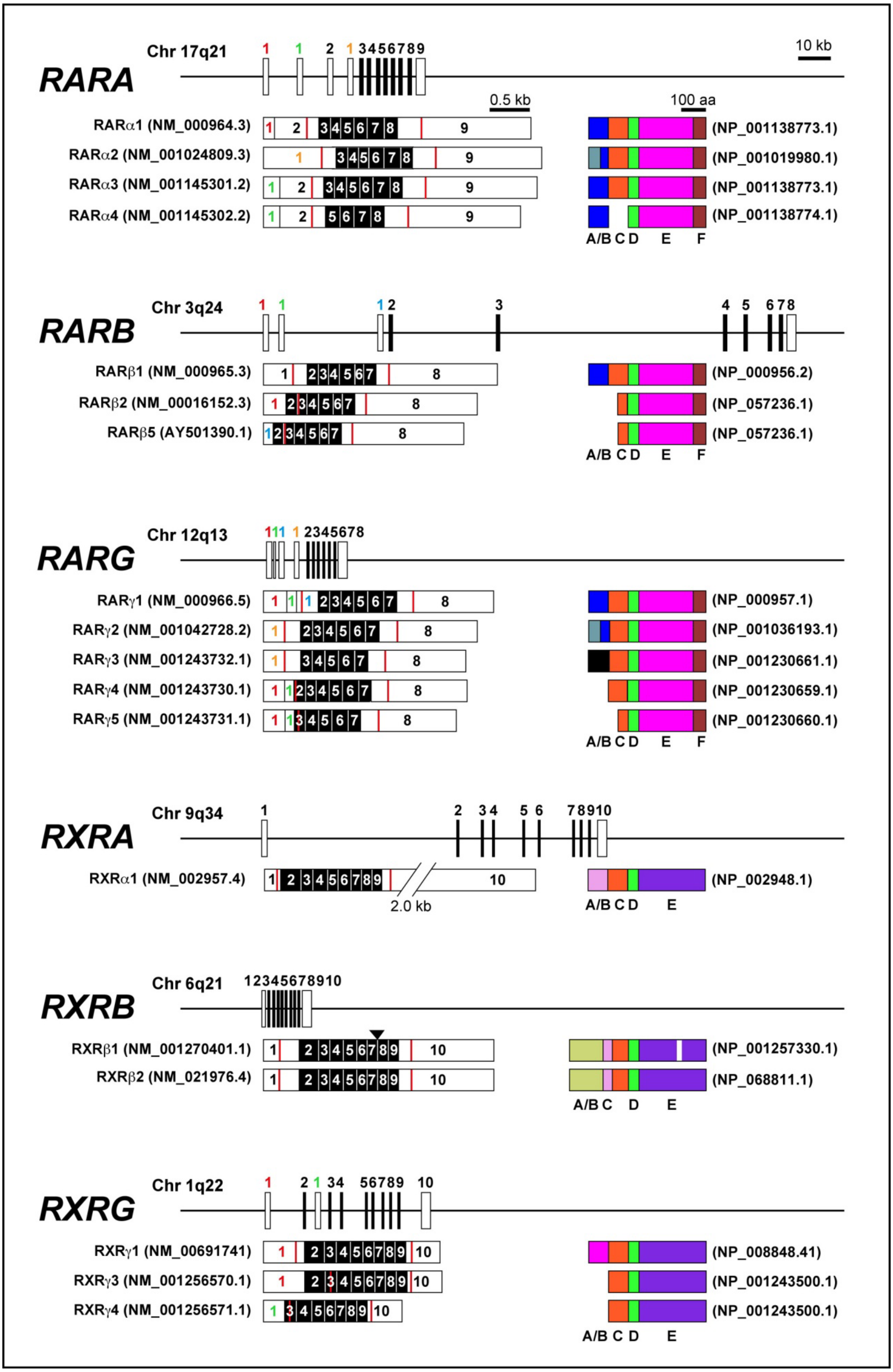

RARA
Chr 17q21
10 kb
0.5 kb
100 aa
RARα1 (NM_000964.3)
RARα2 (NM_001024809.3)
RARα3 (NM_001145301.2)
RARα4 (NM_001145302.2)
(NP_001138773.1)
(NP_001019980.1)
(NP_001138773.1)
(NP_001138774.1)
A/B C D E F
RARB
Chr 3q24
RARβ1 (NM_000965.3)
RARβ2 (NM_00016152.3)
RARβ5 (AY501390.1)
(NP_000956.2)
(NP_057236.1)
(NP_057236.1)
A/B C D E F
RARG
Chr 12q13
RARγ1 (NM_000966.5)
RARγ2 (NM_001042728.2)
RARγ3 (NM_001243732.1)
RARγ4 (NM_001243730.1)
RARγ5 (NM_001243731.1)
(NP_000957.1)
(NP_001036193.1)
(NP_001230661.1)
(NP_001230659.1)
(NP_001230660.1)
A/B C D E F
RXRA
Chr 9q34
RXRα1 (NM_002957.4)
2.0 kb
(NP_002948.1)
A/B C D E
RXRB
Chr 6q21
RXRβ1 (NM_001270401.1)
RXRβ2 (NM_021976.4)
(NP_001257330.1)
(NP_068811.1)
A/B C D E
RXRG
Chr 1q22
RXRγ1 (NM_00691741)
RXRγ3 (NM_001256570.1)
RXRγ4 (NM_001256571.1)
(NP_008848.41)
(NP_001243500.1)
(NP_001243500.1)
A/B C D E


**Suppl. Fig. S4**

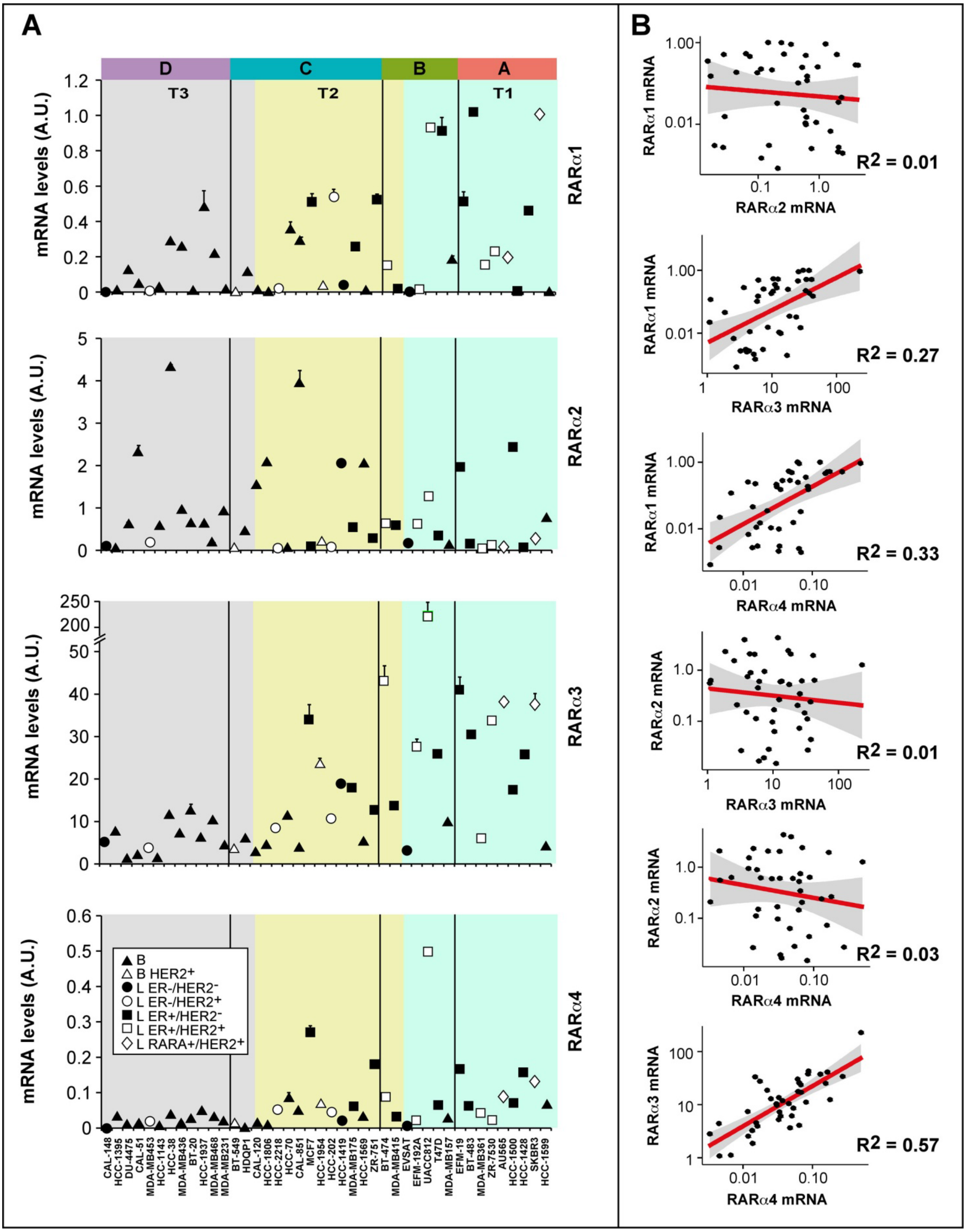
A
D
C
B
A
T3
T2
T1
mRNA levels (A.U.)
RARα1
RARα2
RARα3
RARα4
B
B HER2+
L ER-/HER2-
L ER-/HER2+
L ER+/HER2-
L ER+/HER2+
L RARA+/HER2+
CAL-148
HCC-1395
DU-4475
CAL-51
MDA-MB453
HCC-1143
HCC-38
MDA-MB436
BT-20
HCC-1937
MDA-MB468
MDA-MB231
BT-549
HDQP1
CAL-120
HCC-1806
HCC-2218
HCC-70
CAL-851
MCF7
HCC-1954
HCC-202
HCC-1419
MDA-MB175
HCC-1569
ZR-751
BT-474
MDA-MB415
EVSAT
EFM-192A
UACC812
T47D
MDA-MB157
EFM-19
BT-483
MDA-MB361
ZR-7530
AU565
HCC-1500
HCC-1428
SKBR3
HCC-1599
B
RARα1 mRNA
RARα2 mRNA
R2 = 0.01
RARα3 mRNA
R2 = 0.27
RARα4 mRNA
R2 = 0.33
R2 = 0.01
R2 = 0.03
R2 = 0.57

**Suppl. Fig. S5**

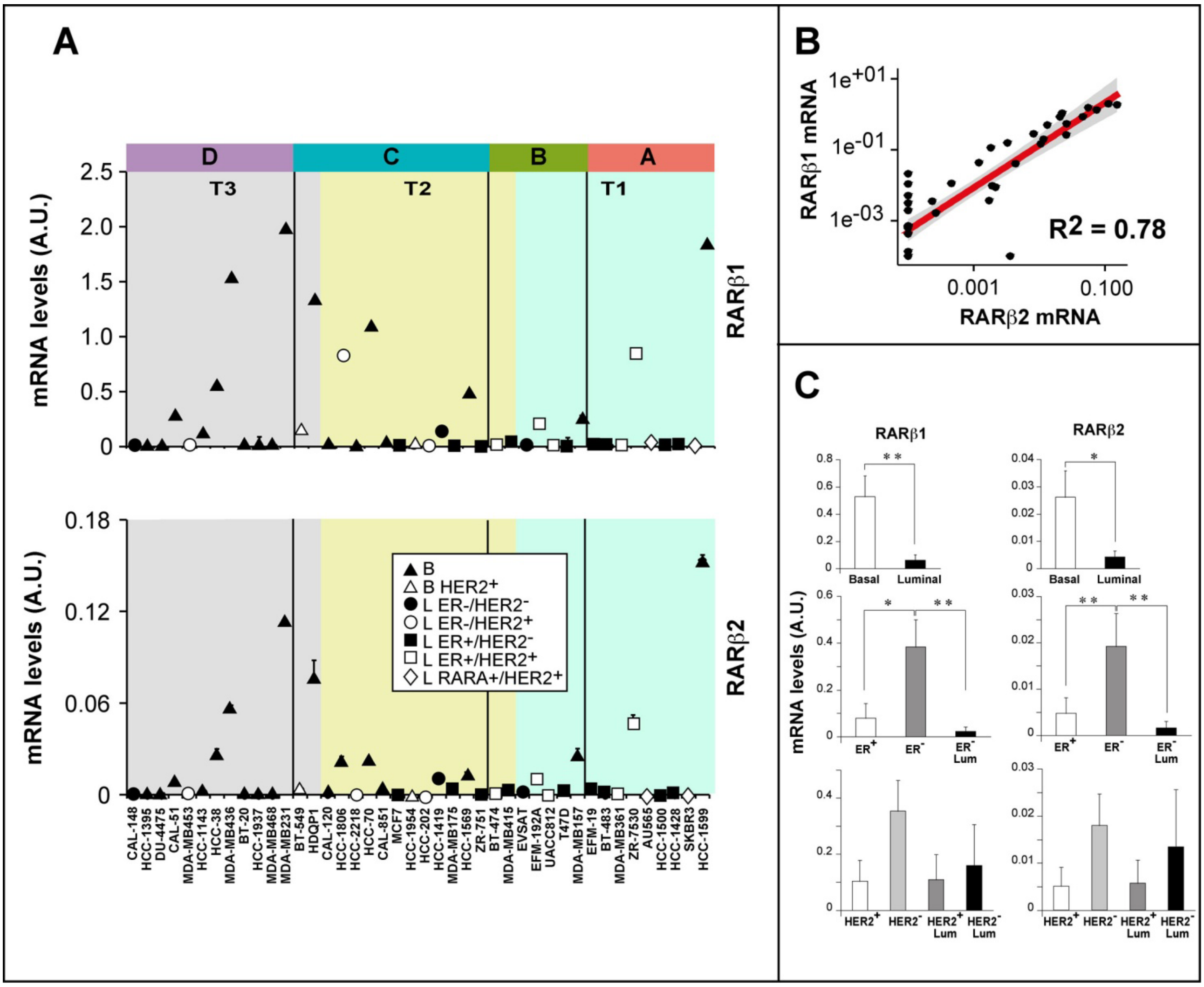
A
B
C
D
C
B
A
T3
T2
T1
mRNA levels (A.U.)
RARβ1
RARβ2
B
B HER2+
L ER-/HER2-
L ER-/HER2+
L ER+/HER2-
L ER+/HER2+
L RARA+/HER2+
RARβ1 mRNA
RARβ2 mRNA
R2 = 0.78
Basal
Luminal
ER+
ER-
ER- Lum
HER2+
HER2-
HER2+ Lum
HER2- Lum


**Suppl. Fig. S6**

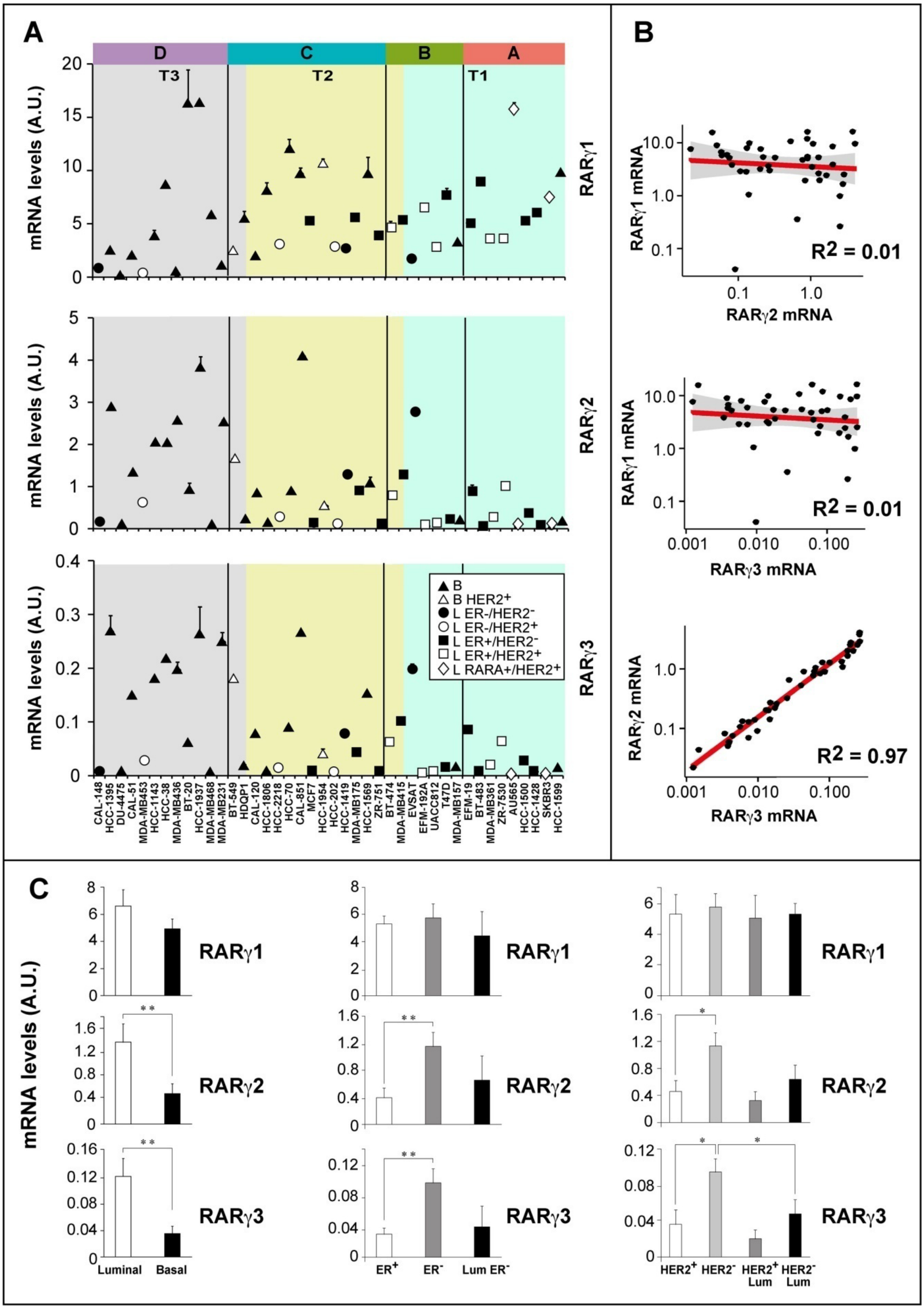
A
D
C
B
A
T3
T2
T1
mRNA levels (A.U.)
RARγ1
RARγ2
RARγ3
▲ B
△ B HER2+
● L ER-/HER2-
○ L ER-/HER2+
■ L ER+/HER2-
□ L ER+/HER2+
◇ L RARA+/HER2+
CAL-148
HCC-1395
DU-4475
CAL-51
MDA-MB453
HCC-1143
HCC-38
MDA-MB436
BT-20
HCC-1937
MDA-MB468
MDA-MB231
BT-549
HDQP1
CAL-120
HCC-1806
HCC-2218
HCC-70
CAL-851
MCF7
HCC-1954
HCC-202
HCC-1419
MDA-MB175
HCC-1569
ZR-751
BT-474
MDA-MB415
EVSAT
EFM-192A
UACC812
T47D
MDA-MB157
EFM-19
BT-483
MDA-MB361
ZR-7530
AU565
HCC-1500
HCC-1428
SKBR3
HCC-1599
B
RARγ1 mRNA
RARγ2 mRNA
RARγ3 mRNA
R2 = 0.01
R2 = 0.01
R2 = 0.97
C
mRNA levels (A.U.)
Luminal
Basal
ER+
ER-
Lum ER-
HER2+
HER2-
HER2+ Lum
HER2- Lum

**Suppl. Fig. S7**

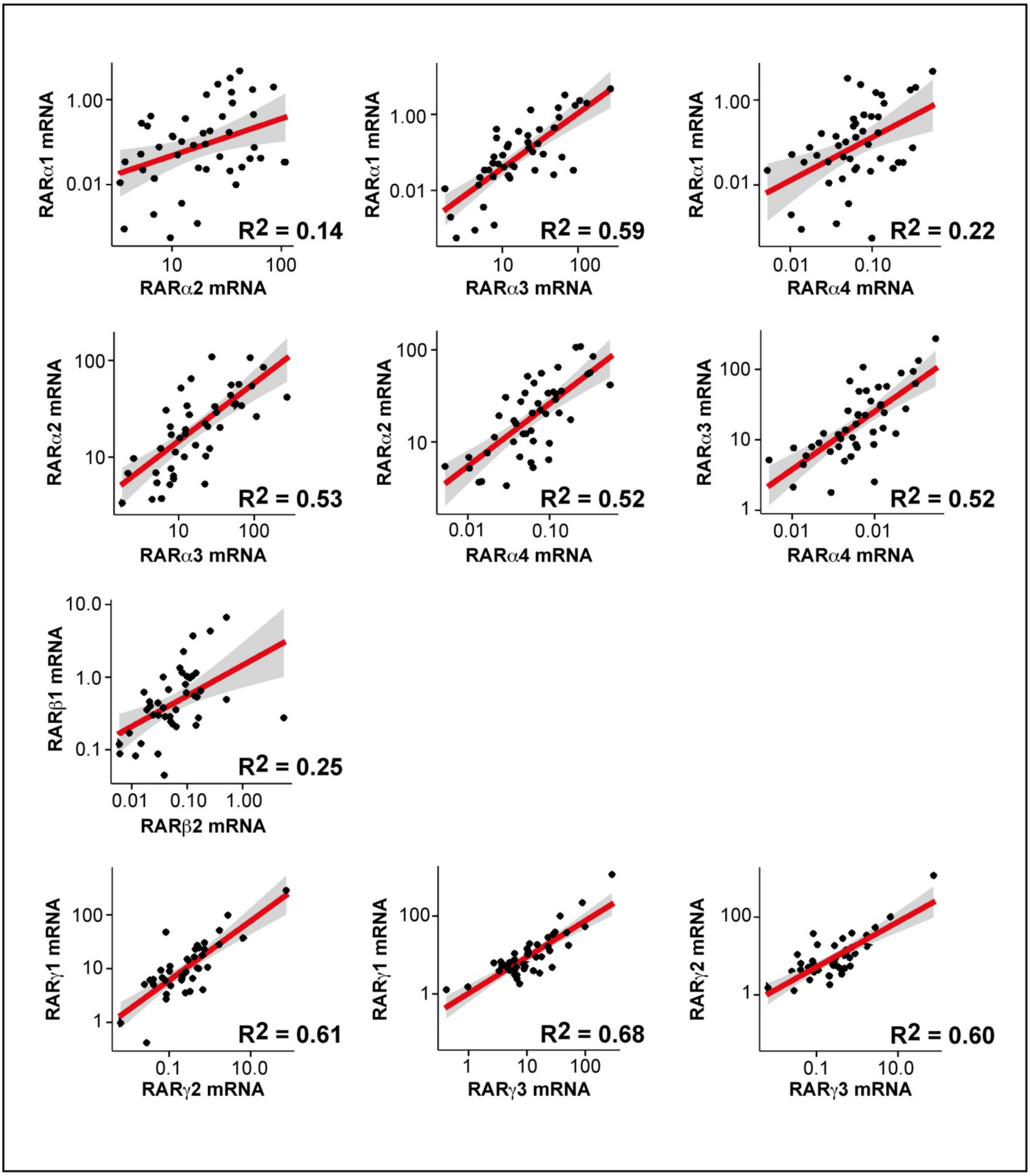
RARα1 mRNA
RARα2 mRNA
$R^2$ = 0.14
RARα1 mRNA
RARα3 mRNA
$R^2$ = 0.59
RARα1 mRNA
RARα4 mRNA
$R^2$ = 0.22
RARα2 mRNA
RARα3 mRNA
$R^2$ = 0.53
RARα2 mRNA
RARα4 mRNA
$R^2$ = 0.52
RARα3 mRNA
RARα4 mRNA
$R^2$ = 0.52
RARβ1 mRNA
RARβ2 mRNA
$R^2$ = 0.25
RARγ1 mRNA
RARγ2 mRNA
$R^2$ = 0.61
RARγ1 mRNA
RARγ3 mRNA
$R^2$ = 0.68
RARγ2 mRNA
RARγ3 mRNA
$R^2$ = 0.60

**Suppl. Fig. S8**

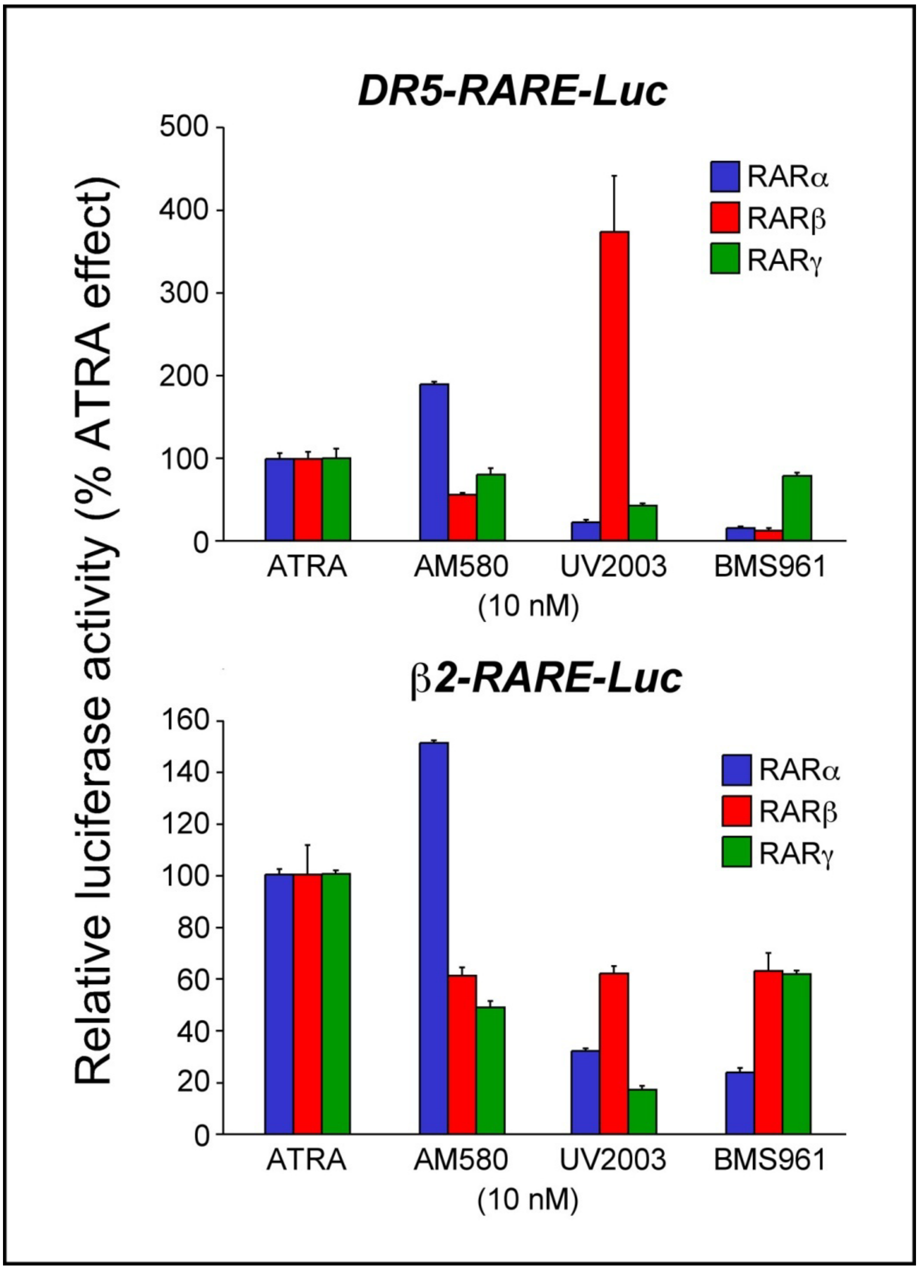
DR5-RARE-Luc
Relative luciferase activity (% ATRA effect)
500
400
300
200
100
0
RARα
RARβ
RARγ
ATRA
AM580
UV2003
BMS961
(10 nM)
β2-RARE-Luc
160
140
120
100
80
60
40
20
0
RARα
RARβ
RARγ
ATRA
AM580
UV2003
BMS961
(10 nM)

**Suppl. Fig. S9**

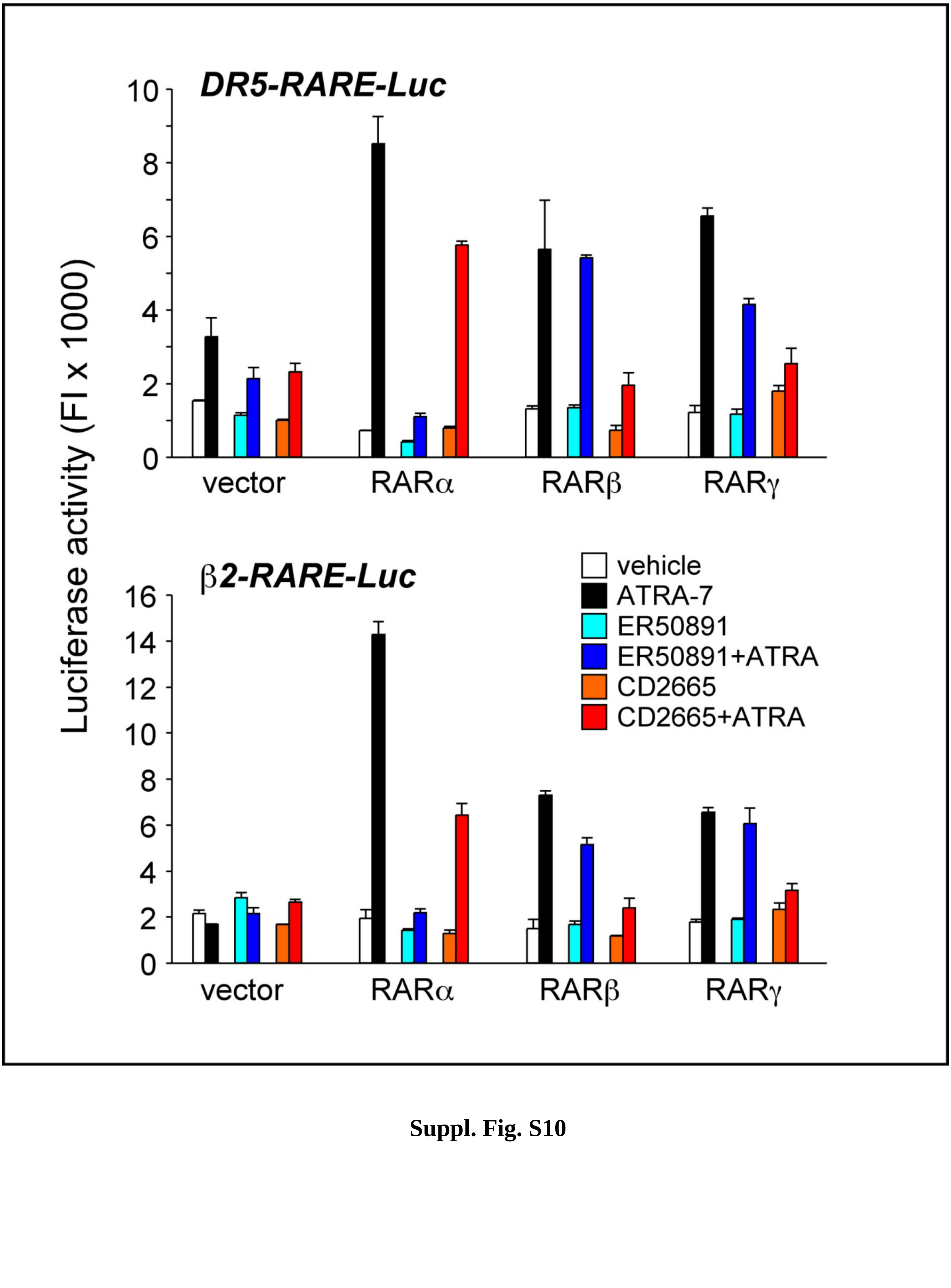


**Suppl. Fig. S10**

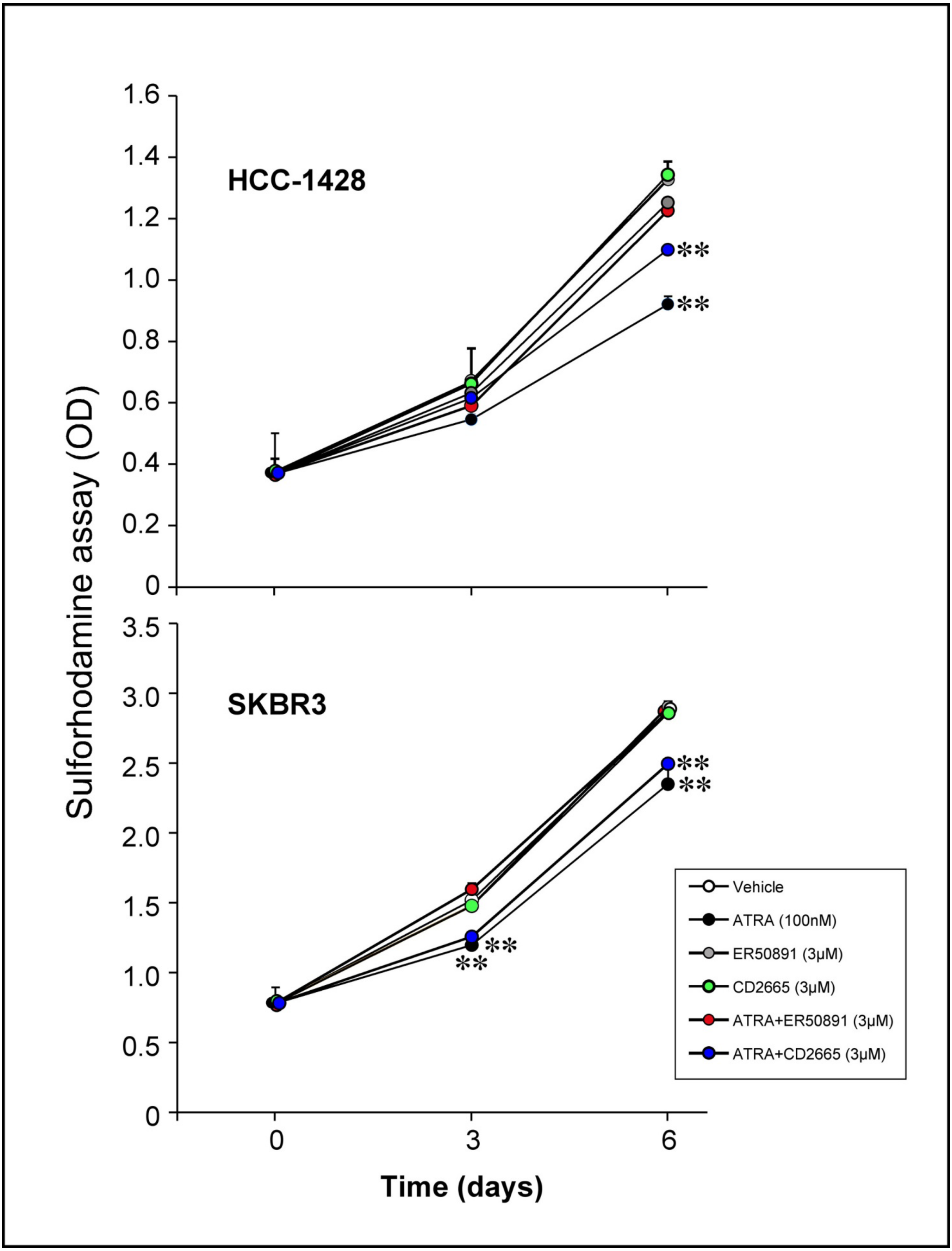


**Suppl. Fig. S11**

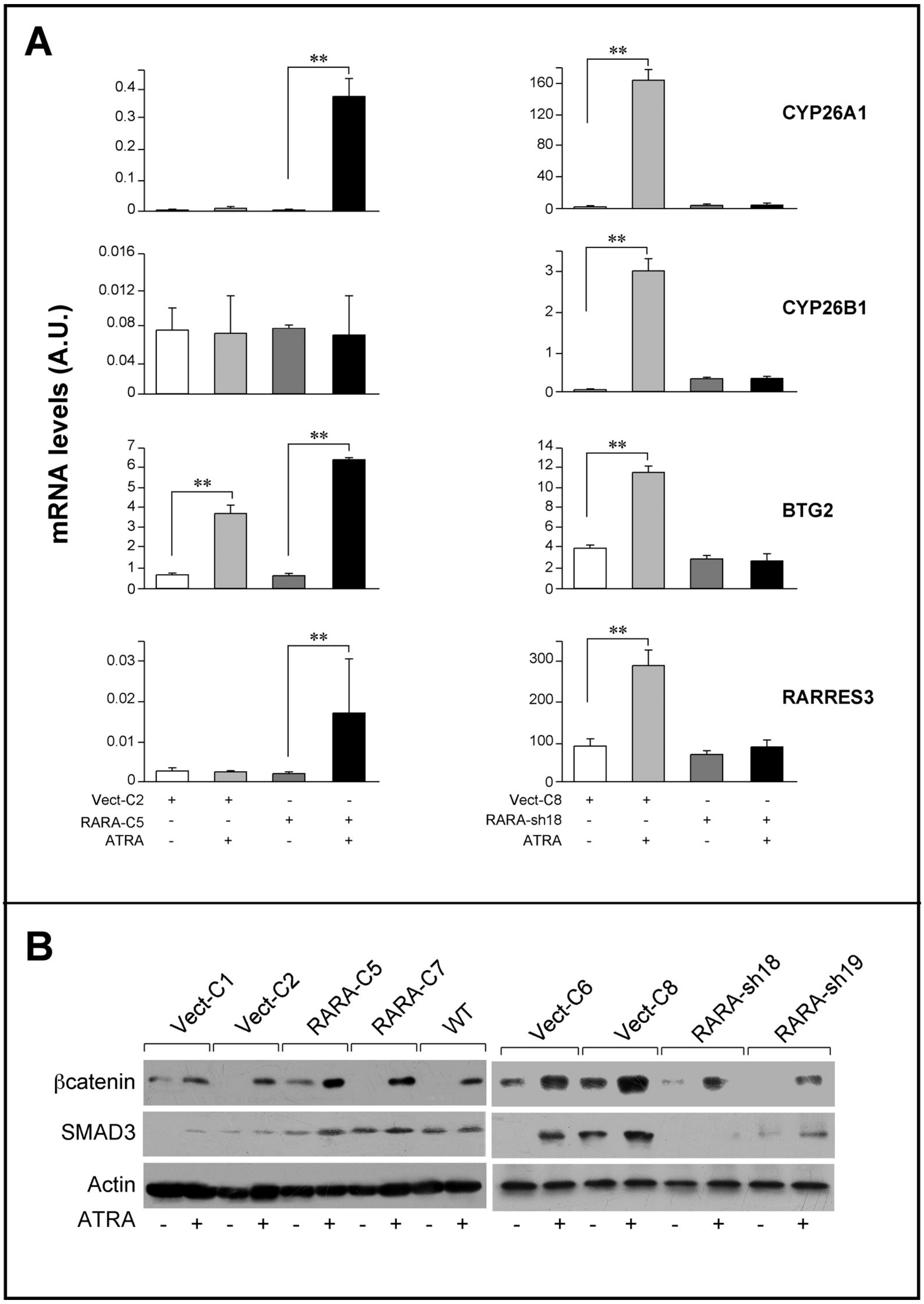


**Suppl. Fig. S12**

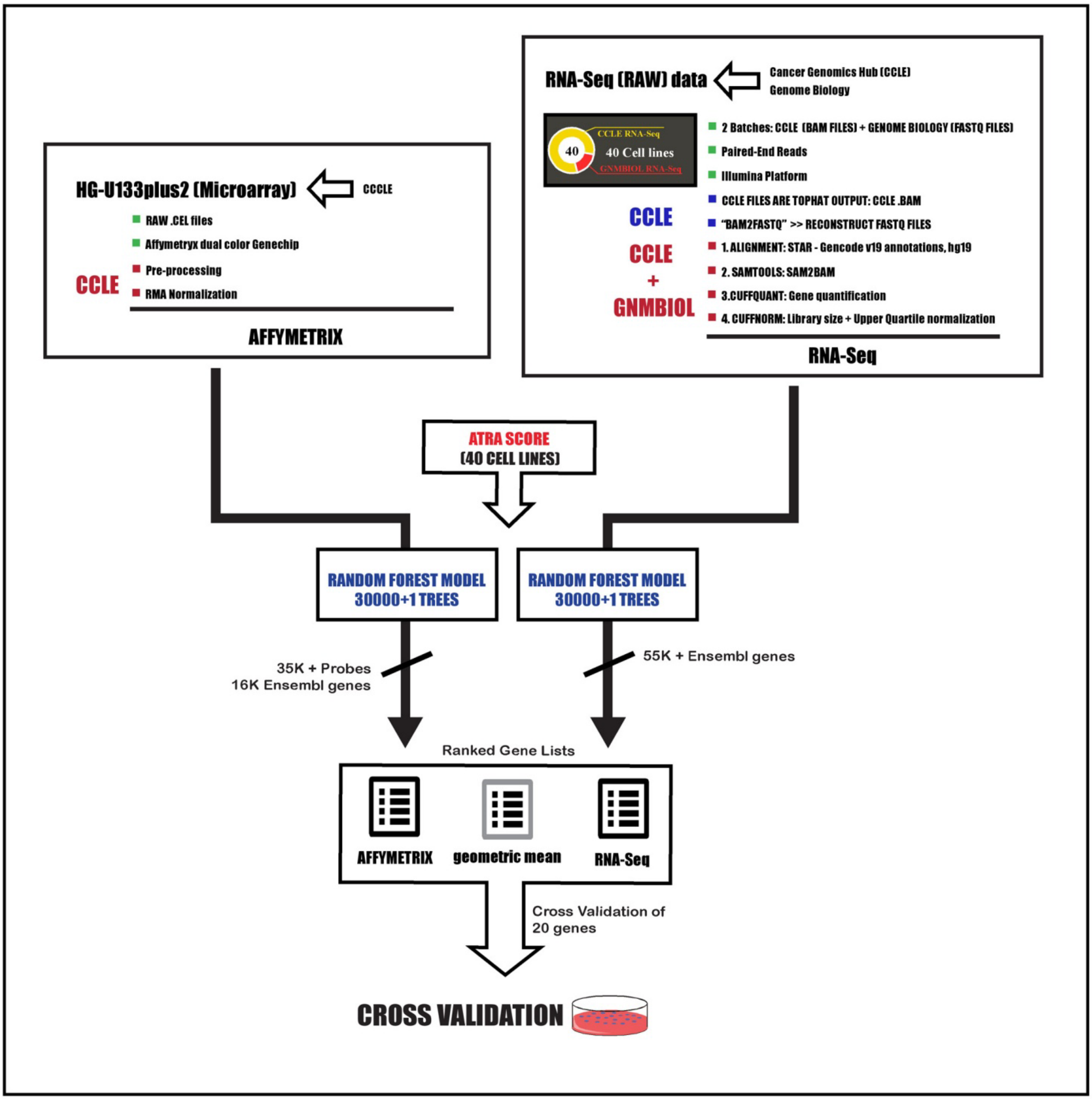


**Suppl. Fig. S13**

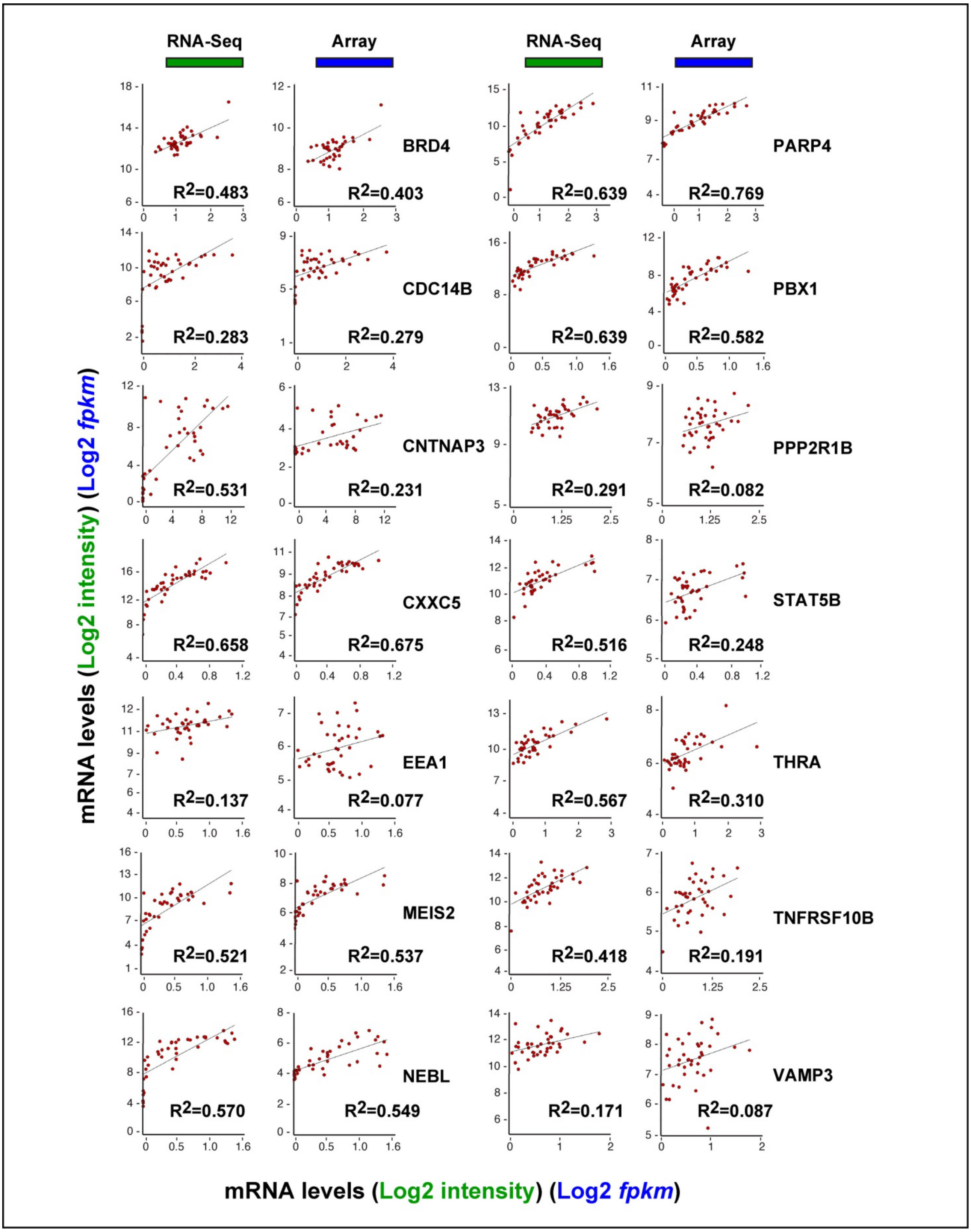


**Suppl. Fig. S14**

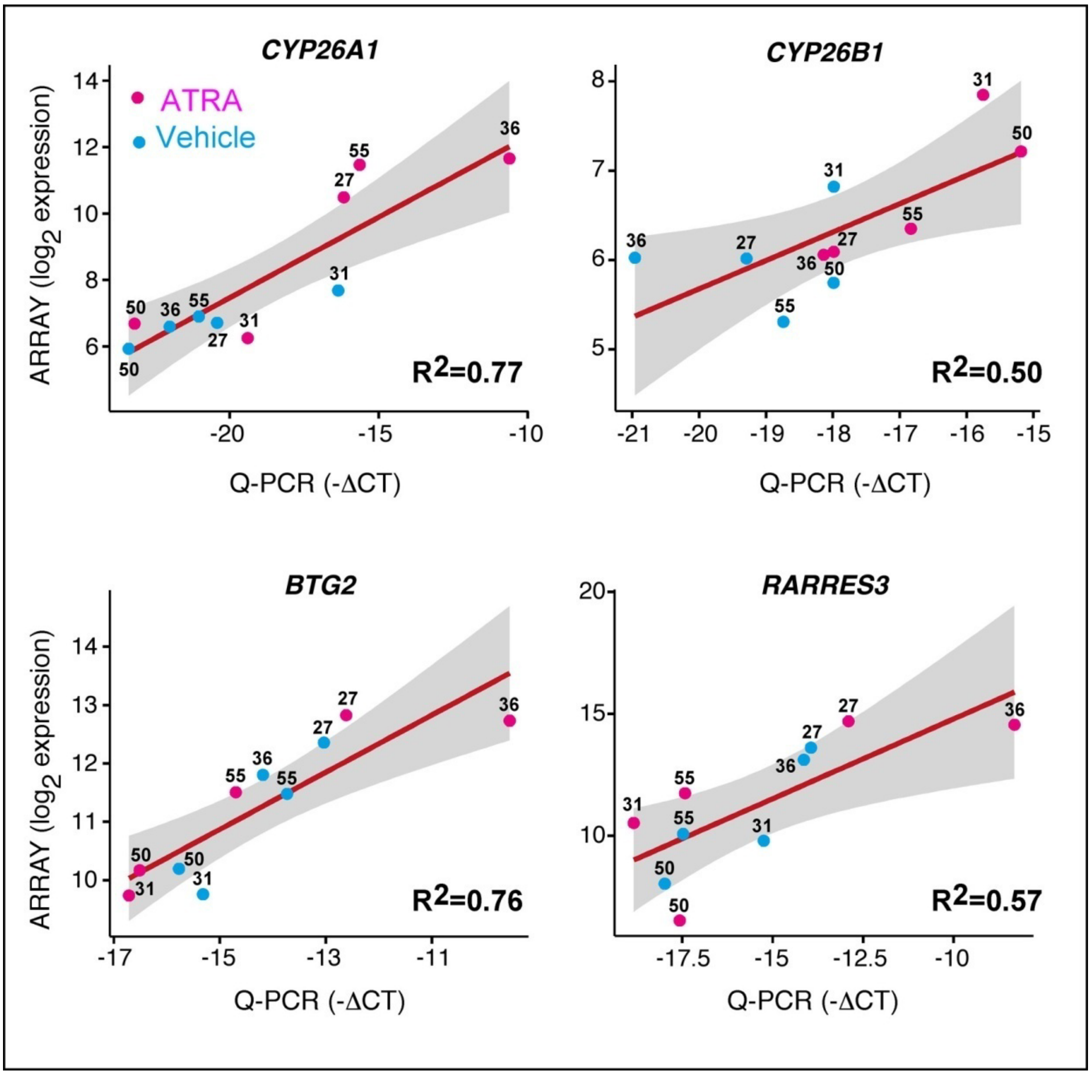


**Suppl. Fig. S15**
