# Supplementary material for: Cellular and molecular determinants of all-trans retinoic acid sensitivity in breast cancer: Luminal phenotype and RARα expression
Source: EMBO Mol Med. 2015 Apr 17;7(7):950–72. doi: 10.15252/emmm.201404670 (PMC4520659; doi:10.15252/emmm.201404670)
Supplement: Supplementary file 10 [file emmm0007-0950-sd10.pdf]

EMBO
Molecular Medicine

Cellular and molecular determinants of all-trans retinoic acid sensitivity in breast cancer: luminal phenotype and RAR expression

Floriana Centritto, Gabriela Paroni, Marco Bolis, Silvio Ken Garattini, Mami Kurosaki, Maria Monica Barzago, Adriana Zanetti, James Neil Fisher, Mark Francis Scott, Linda Pattini, Monica Lupi, Paolo Ubezio, Francesca Piccotti, Alberto Zambelli, Paola Rizzo, Maurizio Gianni', Maddalena Fratelli, Mineko Terao and Enrico Garattini

Corresponding author: Enrico Garattini, IRCCS-Istituto di Ricerche Farmacologiche "Mario Negri"

Review timeline:

Submission date:	19 September 2014
Editorial Decision:	23 October 2014
Revision received:	16 February 2015
Editorial Decision:	20 March 2015
Revision received:	23 March 2015
Accepted:	25 March 2015

Transaction Report:

(Note: With the exception of the correction of typographical or spelling errors that could be a source of ambiguity, letters and reports are not edited. The original formatting of letters and referee reports may not be reflected in this compilation.)

1st Editorial Decision 23 October 2014

Thank you for the submission of your manuscript to EMBO Molecular Medicine. We have now heard back from the three Reviewers whom we asked to evaluate your manuscript. We are sorry that it has taken longer than usual to get back to you on your manuscript. In this case we experienced unusual difficulties in securing appropriate reviewers and then obtaining their evaluations in a timely manner. Further to this, I wished to discuss the evaluations with my colleagues.

As you will see the issues raised are few but fundamental. Although I will not dwell into much detail, I would like to highlight the main points.

Firstly, while Reviewer 2 is clearly positive, Reviewers 1 and 3 mention similar concerns, albeit with different emphasis and perspective. The main one is the perceived lack of clinical relevance of your findings: Both Reviewers note that the excessive emphasis on cell lines vs. actual tumour samples detracts from potential usefulness and would have liked to see the contrary. Furthermore a most crucial point is that they fail to be convinced that the ATRA sensitivity score can actually be useful in the clinic to predict response in patients. In essence, while Reviewer 3 appears globally more positive, in aggregate the concerns are almost overlapping. Both, however do recognise the validity of your conclusions that RARalpha might be responsible for retinoid sensitivity,

Reviewers 1 and 3 clearly have diverging opinions on the methods used to collect the data on hormone responsiveness, and while we do not endorse the application of more advanced technologies for the sake of using them, we agree that Reviewer 1 does have a strong point that the information gathered might have been more useful to the community and overall more meaningful.

Reviewer 2, as mentioned is essentially positive, and raises a few issues. I do note, however, again an emphasis on the tumour data.

In conclusion, while publication of the paper cannot be considered at this stage, given the potential interest of your findings and after internal discussion, we have decided to give you the opportunity to address the above concerns.

We are thus prepared to consider a substantially revised submission, with the understanding that the Reviewers' concerns must be addressed with additional experimental data where appropriate and that acceptance of the manuscript will entail a second round of review. While Reviewer 1's point on overexpressing other RARs or PPAR appears of limited relevance for the study, we do ask you to address all the other points discussed above and the other items listed by the Reviewers. As for obtaining additional data from gene expression studies, as mentioned by Reviewer 1, I do encourage you to develop your study as far as realistically possible in this sense. The overall aim is to significantly upgrade the clinical relevance and usefulness of the dataset, which of course is of paramount importance for our title.

I understand that if you do not have the required data available at least in part, to address the above, this might entail a significant amount of time, additional work and experimentation and might be technically challenging, I would therefore understand if you chose to rather seek publication elsewhere at this stage. Should you do so, we would welcome a message to this effect.

Please note that it is EMBO Molecular Medicine policy to allow a single round of revision only and that, therefore, acceptance or rejection of the manuscript will depend on the completeness of your responses included in the next, final version of the manuscript.

As you know, EMBO Molecular Medicine has a "scooping protection" policy, whereby similar findings that are published by others during review or revision are not a criterion for rejection. However, I do ask you to get in touch with us after three months if you have not completed your revision, to update us on the status. Please also contact us as soon as possible if similar work is published elsewhere.

***** Reviewer's comments *****

Referee #1 (Comments on Novelty/Model System):

In order to meaningfully analyse breast cancer they should have incorporated more primary human samples.

Referee #1 (Remarks):

The authors of this paper characterize the retinoid responsiveness of 42 breast cancer cell lines and in additional experiments they use human cancer slices to provide support for their findings in a quasi in vivo setting. In addition they identify the predominant RAR isoform and link it to retinoid sensitivity and also make an attempt to define a signature of retinoid sensitivity by comparing microarray and RNA-Seq data.
The provided data is certainly useful characterization of the cell lines and have a limited utility in characterizing tumors as well.
However the approaches used are not really state of the art, most of the findings are trivial and incomplete and it is not clear how one would use it in proposing clinical studies.
Major and conceptual points of criticism:
1. Measuring cell proliferation, Ki67 positivity and a few target genes are not really state of the art. Hormonal responsiveness is measured by more complex gene expression studies, genome-wide localization (ChIP-Seq) and correlation and impact on major breast cancer driving oncogenic pathways and interaction with such pathways i.e ER mediated pathways in ER+ cells.
2. That is hardly surprising that the most abundant retinoic acid receptor (RARa) is responsible for the effect of ATRA. Although the study nicely documents it and it is commendable, alone this finding is not a major finding.
3. Although the authors reference Noa Noy's work on the dichotomy of ATRA signaling by RARa

and PPARb/d they completely ignore it their analysis. What happens if they overexpress another retinoid receptor or PPARb/d ? Is there an impact on the phenotype and/or gene expression profile for example?
4. The authors also completely ignore the literature on RXR mediated chemoprevention in breast cancer, mostly Powel Brown's work. ATRA would activate RXR regulated gene expression as well and it is likely to have an impact on cell proliferation, invasion etc.
5. I am puzzled by the retinoid signature data presented in the last part of the manuscript. What can this be used for ? Is it predictive in any ways for treatment, response, survival ?
6. Is there any evidence that retinoid signaling is active in breast cancer samples and it correlates with an of the clinical parameters ?
In summary, the authors provide a large amount of potentially useful data on retinoid-regulated cell proliferation and a few other parameters, but the study does not reach the level of a major journal in molecular medicine, because of the limited and somewhat outdated methodology and the not apparent clinical utility.

Referee #2 (Remarks):

The potential and use of retinoic acid as a cytostatic and differentiation treatment in cancer has long history. This manuscript provides an approach to stratifying breast cancer patients. The authors analyze 42 human breast cancer cell lines and 30 breast tumors to show that expression of the RARα3 mRNA and its corresponding protein are directly associated with retinoid-sensitivity, the luminal phenotype and ER-positivity. The comprehensive profiling of the cell lines and the introduction of a breast tumor slice short term cultures provides a robust and clinically relevant evaluation of response to all-trans retinoic acid (ATRA). The authors used TCGA data to confirm that FABP5 and PPARβ/δ are negatively associated with ATRA-sensitivity and experimentally manipulated RARα, which they identify as a gene that is highly ranked in the ATRA sensitivity fingerprint, to support functional relevance. The manuscript is clearly written and data analysis appears appropriate.

Minor Comments
1 One would like know whether ATRA exposure altered histology of tumor slice culture and to see examples of the readout of Ki67 included in the manuscript.
2 Were the proliferating cells in the tumor slice cultures phenotypically similar (e.g. ER-ve)?
3 The figure legends contain information that should be moved to methods (e.g. Figure 2 regarding slice preparation and analysis; Fig 10, methods for heatmap analysis).
4 Please note whether the scoring of Ki67 was blinded as to treatment or replicated by independent observers.

Referee #3 (Comments on Novelty/Model System):

1)the methodologies used are up to date and provide significant inside info to the RA sensitivity/ non-sensitivity.
2)Because of the broad scope of approach used (large number of cell lines, plus breast cancer tissues, plus in vivo model(s), a general conclusion relating RA sensitivity/ non-sentivity to other markers could be shown, although no perfect correlation between ER+/ER- , HER2+/HER2- luminal/basal and ATRA responsiveness/ unresponsiveness was observed.
3) the medical impact of this report could be significant since RA or derivatives could potentially lead to improved treatment of certain breast cancers

Referee #3 (Remarks):

The authors investigate ATRA sensitivity of a large number of breast cancer cell lines and extend their studies also to primary tumor slices. To measure ATRA sensitivity the authors develop a new ATRA score which they claim is necessary to accurately determine ATRA sensitivity. While there might be some benefit in their new scoring system this reviewer is not convinced that the NCI scoring system would have given relevant ATRA sensitivity scores, especially given the fact that the NCI scoring system is and has been used as a general system to identify many compounds of potential clinical relevance. Also the authors do not show any data that the outcome with their

system results in significantly different results. It is to be kept in mind that the ultimate goal here should be to provide data that ATRA or a selective derivative could be used as a novel adjuvant in breast cancer therapy and this reviewer does not see the relevance of this "new scoring system" for determining whether a patients breast cancer is responsive to retinoid. In contrast and importantly the authors point out that gene expression profiles developed here could be used to prescrene breast tumors for their sensitivity to retinoids. In this regard the findings reported here that luminal ER+ tumors are in general responsive. If their preliminary results mentioned in the discussion that approx. 70% of luminal breast cancers are responsive to ATRA, then considering the probably benign side effects of a retinoid adjuvant therapy all luminal breast cancers might be treated in a first approach. Overall the data provided here delineate strategies on novel retinoid therapies for breast cancer. An important finding is that a RARa subtype is responsible for the retinoid sensitivity and that the authors can support this finding with the usage of selective compounds . The large volume of cell line data are of interest in that they are consistent in what the authors find in real tumors but become less important the more data on actual tumors are presented. Accordingly It might be useful for the reader of this journal to summarize some of the cell line data and focus on the tumor data and gene expression data observed here upon ATRA treatment.

1st Revision - authors' response 16 February 2015

GENERAL COMMENTS

We read with great interest the comments of the reviewers and performed a number of new experiments that we included in the revised version of the manuscript. As requested by the reviewers, we increased the number of primary tumors on which we performed short-term tissue cultures from the original 30 to 45. We focused our attention on Luminal and ER^+ tumors, given their original propensity to undergo growth inhibition upon ATRA challenge. The results obtained on the new cases confirm that approximately 70% of Luminal A and Luminal B tumors are responsive to ATRA. The new primary tumors were used to expand the data on the expression of the various splicing variants of the RAR isoforms and to strengthen some of the observed associations between RARα, RARβ and RARγ splicing variants and tumor cell phenotypes as well as ATRA sensitivity. Once again the data confirm the relevance of RARα3 for the anti-tumor action of ATRA. In addition, we performed whole-genome gene-expression experiments on 16 of these tumors (11 Luminal and 5 Basal samples) following ex vivo challenge with vehicle and ATRA primarily to study and compare the type of transcriptional responses in Luminal and Basal cancers, as detailed in the new version of the manuscript. We performed a number of state-of-the-art bio-informatic post-hoc analyses on the results obtained. These studies are described and discussed in the entirely new last section of the manuscript entitled "*Transcriptional responses to ATRA in short-term cultures of mammary tumors*". The microarray gene expression data were deposited in the public Arrayexpress database (accession No. E-MTAB-3313) and the Reviewers can access the raw data using the following coordinates: Username: Reviewer_E-MTAB-3313; Password: dw7vwyG2. The new version of the manuscript contains a large number of new experimental data which forced us to increase the number of Figs. from 10 to 12, to expand the No. of Suppl. Tables from 4 to 8 (Suppl. Tables S1-S8) and the No. of Suppl. Figs from 13 to 15 (Suppl. Figs 1-15). The introduction of the new data led to an extensive reorganization of the manuscript. In spite of this, we kept the total length of the text around the suggested 60,000 characters (63,119 characters). We also expanded the SUPPLEMENTARY INFORMATION, as we moved there some of the original MATERIALS AND METHODS. We think that the comments of the Reviewers were instrumental in improving the quality of our manuscript and we take this opportunity to thank them for their precious help.

SPECIFIC RESPONSES TO THE REVIEWERS'COMMENTS

Reviewer No. 1

As requested by the reviewer, we have increased the number of primary tumors analyzed from 30 to 45, extending predominantly the number of Luminal A and Luminal B samples given the fact that these types of breast cancer are particularly sensitive to the action of ATRA. In addition, we performed a series of new whole-genome gene-expression experiments on tissue slices incubated with vehicle and ATRA, as detailed in the response to point 1 (see below). These last new data are

contained in Fig. 12 and validated in Suppl. Fig. S15. The new gene-expression data were the object of a number of state-of-the-art bio-informatic analyses which are described in Fig. 12 and Suppl. Tables S5-S8. All these new data are described and discussed in the RESULTS (page 16, lines 4-26; page 17, lines 1-25; page 18, lines 1-2) under the heading "*Transcriptional responses to ATRA in short-term cultures of mammary tumors*" and DISCUSSION (page 18, lines 21-26; page 19, lines 1-3) sections. Detailed answers to the specific points raised by the Reviewer are presented below.

1. The focus of the studies performed with the short-term tissue cultures is to validate and extend the results obtained in the large panel of cell lines analyzed. In particular, the major point of the experiments is to confirm that a luminal phenotype and ER-positivity are two major determinants of the anti-proliferative action of ATRA. Though useful and appropriate for the above mentioned goals of the study, the model of short term cultures has some limitations which need to be considered for the choice of an appropriate readout of ATRA sensitivity. With respect to this, the selection of Ki67 as the readout of the anti-proliferative activity of ATRA was dictated by two main considerations. Ki67 is an established marker routinely used in the clinics to assess the proliferation rate of mammary tumors. In addition, Ki67 is rapidly down-regulated by a number of anti-proliferative agents (see the referenced article by Alagesan et al, 2015), which has also been demonstrated in our experimental conditions. This last characteristic is of the outmost importance given the relatively short exposure times to ATRA that our tissue culture model allows. In fact, 48 hours was determined to be the maximal time interval maintaining tumor cell viability in tissue slices. To make these points clear and to explain the choice of Ki67, we added an appropriate explanatory paragraph in the RESULTS section (page 6, lines 21-26; page 7, lines 1-3).

To address the second point raised by the Reviewer, we performed whole-genome gene expression experiments in 16 of the breast cancer samples considered, as already mentioned in the general comments above. To this purpose RNA was extracted from tissue slices cultured in the presence of vehicle and ATRA and used for gene-expression microarray experiments. Eleven of the cases analyzed are luminal and ER^+, while 5 of them are basal. This allowed us to evaluate whether the basal and luminal/ ER^+ phenotypes, which are associated with general resistance and sensitivity to ATRA, show different gene-expression responses to the retinoid. We have characterized the transcriptional responses to ATRA in Luminal tumors indicating an anti-estrogenic action of the retinoid (Fig. 12D). The interactome analysis demonstrates an impact of ATRA on major breast cancer oncogenic pathways. We would like to point out that the limited quantity of tissue and other technical limitations did not allow us to perform the Chip-seq experiments, which are certainly going to be the object of future studies.

2. There is a long-standing debate as to which is the RAR isoform responsible for the anti-tumor responses to ATRA and derived retinoids. In particular, there are scattered data supporting the idea that RARβ is a major determinant of ATRA-dependent responses (see Connolly et al, 2013). In addition, significant levels of RARγ are present in breast cancer cell lines and mammary tumors, suggesting that this RAR isotype may have a role in the anti-tumor action of ATRA. With respect to this, it is interesting to notice that activation of RARγ has been reported to induce rather than inhibit the growth of tumor cells in a mouse model of breast cancer (Bosch et al, 2012) (page 10, line 8). It should also be pointed out that our work is the first one to report on the expression of the different splicing variants of RARs in breast cancer cells and primary tumors. Therefore, we believe that the identification of RARα3 as a major determinant of ATRA activity is a novel and important finding, as also acknowledged by Reviewers 2 and 3. In fact, it represents a fundamental step along the characterization of the molecular mediators of ATRA activity and it supports the design of therapeutic strategies based on the use of RARα agonists for the treatment and prevention of luminal breast cancer.

3. PPARβ/δ is the object of numerous analyses in our study, as indicated by the results presented in Fig. 4, Fig. 5 and Fig. 6A. As shown in the new version of Fig. 4, no difference in the basal expression levels of PPARβ/δ is observed if luminal and basal, ER^+ and ER^- or $HER2^+$ and $HER2^-$ cell lines are compared. In contrast, Fig. 5A and Fig. 5B indicate that the levels of PPARβ/δ are higher in basal than all the other breast cancer subtypes. Fig. 6A indicates that PPARβ/δ is higher in ATRA-sensitive basal cell lines. Thus, at present, the situation is complex and it does not allow to draw definitive conclusions as to the negative or positive action of PPARβ/δ on ATRA-dependent cell growth inhibition. Given this complexity, we did not perform any functional study on the receptor in our panel of cell lines and focused our interest on RARα3 instead. We feel that over-expression and silencing experiments on PPARβ/δ go beyond the scope of the present article.

However, the point raised by the Reviewer is of doubtless interest and future functional studies will address it.

4. To the best of our knowledge, which is based on a large number of available studies, ATRA is unable to bind and activate RXR. Indeed, the retinoid is a well established pan-RAR agonist, as it binds RARα, β and γ with the same affinity. The point was already discussed in the original version of the INTRODUCTION. We are aware of the seminal work performed by Powel Brown's group on the chemo-preventive potential of RXR activation. However, it should be noticed that this work was performed with bexarotene and rexinoids which, unlike ATRA, are specific RXR agonists. To make the whole point clear we added a short explanatory paragraph in the new version of the INTRODUCTION (page 3, lines 11-16) and we added five relevant references to Brown's work (page 3: Abba et al, 2008; Kim et al, 2006; Kong et al, 2005; Uray & Brown, 2011; Wu et al, 2002).

5. The whole chapter "*Identification of gene-sets associated with ATRA-sensitivity*" regarding the significance of the two gene-sets identified (see RESULTS section, page 14, from line14 onward) has been completely rewritten so as to include the new data presented in Fig 11. As stated from page 15, line 18: "…The two identified gene-sets may be useful for the stratification of patients who are likely to benefit from retinoid-based therapeutic approaches....... The two identified gene-sets are the basis for the generation of an optimized gene signature predictive of ATRA-sensitivity.…". To further clarify the point we added a number of comments on the issue in the DISCUSSION section (page 19, lines 22-26; page 20, lines 1-11). Please also notice that part of the text originally present in the RESULTS section aimed at discussing the potential significance of some of the individual genes present in the two gene-sets have been substantially reduced to make the text more concise and to the point (page 15, lines 6-17). To answer the specific question as to whether there is any association between the two gene-sets identified and any clinical parameter besides ER-positivity and luminality, we used the TCGA database. In particular, we evaluated correlations between overall survival or metastasis-free survival and the two RNA-seq and microarray derived gene-sets using the UCSC genome browser. We found no significant correlation and the results of these analysis are not reported in the manuscript.

6. The point was already partially considered in the original version of the manuscript, as we studied the profile of expression of the various receptors and retinoid binding proteins in the TCGA dataset and in our tissue slices under basal conditions (Fig. 4, Fig. 5 and Suppl. Fig S3). In addition, the new results of the microarray gene-expression analysis performed on short-term tissue-slice cultures demonstrate that a number of established retinoid-responsive genes are induced by ATRA (Fig. 12B). Some of them are indeed induced specifically in *Luminal* tumors, suggesting possible associations with responsiveness to the anti-proliferative action of ATRA (page 16, lines 21-26, page 17, lines 1-7). As for possible associations of any member of the retinoid receptors with clinical parameters, we performed the same type of analysis described under point 5 of the present letter, without finding any significant association, besides the described associations with the ER-positive and the luminal phenotypes. However further studies are in progress.

Reviewer No. 2

1. As suggested by the reviewer, we modified Fig. 2 to show representative fields of the immuno-histochemical analysis of Ki67 (now Fig. 3A). To this purpose, we added microscopic fields of tissue slices treated with vehicle and ATRA. We added one representative case for each of the *Luminal-A*, *Luminal-B*, $HER2^+$ with co-amplification of the *ERBB2*/*RARA* genes and triple-negative subgroups. In general, the slides prepared for the determination of Ki-67 do not show any major morphological changes of the tumour cells. This is partially expected, as treatment with ATRA is limited to 48 hours. In fact, according to the experience developed with our panel of cell lines, this time frame is likely to be too short and insufficient to induce morphological changes associated with the predicted cyto-differentiating and the potential apoptotic responses triggered by ATRA. Although the other point raised by the Reviewer is interesting, we feel that an appropriate analysis of this aspect of ATRA activity goes beyond the scope of the present study and may not be approachable with the model of short-term tissue-slice cultures. A short description of the new version of Fig. 3 is present on page 6, line 21-26 and page 7, lines 1-11. It should be noticed that panels C and D of the previous version of Fig. 2 are now panels A and B of the new version of Fig.

2. The description of the results illustrated by the new version of Fig. 2 have been moved on page 6, lines 7-16 under the heading "*Sensitivity of breast cancer cell-lines to retinoids*".

2. In ER^+ tumors, Ki-67 and ER staining of adjacent slides of several tumor specimens indicate that only a small fraction of the ER^+ tumor cells (up to 10%) is Ki67-positive. In ATRA-sensitive tumors, ER-positivity is stable even following treatment with the retinoid, while the number of Ki-67 positive cells decreases. Nevertheless, the available data do not allow us to draw any conclusion as to potential phenotypic differences between the two sets of cells.

3. As for the legend to Fig. 2 (now Fig. 3), we moved the methodological information regarding the preparation of the slices to the MATERIALS AND METHODS section (page 21, lines 24-26; page 22, lines 1-12), as suggested by the reviewer and we reorganized the legend to the new version of Fig. 3 accordingly. Similarly, the information on the cluster analysis present in the old version of Fig. 10 (now Fig. 11) is now available the MATERIALS AND METHODS section (page 23, lines 15-25).
4. Scoring of Ki67 was performed by several investigators and it was blinded as to treatment. This information is now available in the MATERIALS AND METHODS section (page 22, line 7).

Reviewer No. 3

1. The NCI scoring system is certainly a very efficient tool to assess the action of the vast majority of anti-tumor agents, although it is characterized by a number of limitations, which are thoroughly discussed in the Supplementary Methods section under the chapter entitled "*Determination of the ATRA-score".* However, we observe that proper NCI scores are rarely applied in the available literature. In many cases, the activity of a drug is described using IC_{50} values, which are calculated as the ratio between treated and control signals at a single and arbitrary time point. With respect to this, the NCI method made an important advancement, because growth inhibition is calculated using two time points: the first one is determined at the beginning of the treatment and the second one after 3 days. This method enables a first correction of the contribution to the score of the different growth-kinetic characteristics of each cell line. Nevertheless, it should be pointed out that determination of simple IC_{50} or GI_{50} values is generally appropriate for direct cyto-toxic agents, while it provides inaccurate results for a compound like ATRA. In fact, the anti-tumor action of ATRA is predominantly the result of anti-proliferative and cyto-differentiang effects. In particular, the compound is not a direct cytotoxic agent in breast cancer cells and many other tumor cell types. To clarify this point and to explain the necessity to develop a new sensitivity index, like the *ATRA-score*, for the profiling of our panel of cell lines, we added a whole paragraph in the RESULTS section (page 5, lines 12-20). We think that this addition addresses the point raised by the Reviewer. It should be noticed that strict application of the NCI scoring system to our panel of cell lines provides a profile of sensitivity to ATRA which is different from the one determined with the ATRA score and does not reflect the real relative sensitivity of each cell line to the retinoid (see Fig. 1 included in this response letter). Indeed, the R^2 correlation index between the ATRA-scores and the IC_{50} values calculated for 28 of the cell lines present in the panel is rather low, as shown in Figure, below. It should also be pointed out that the *ATRA-score* was developed and used only to define the relative sensitivity of our cell lines to ATRA. Nowhere in the article have we proposed the use of the *ATRA-score* to evaluate whether a breast cancer patient is responsive to ATRA. We feel that the additions made to the text address the points raised by the Reviewer.

2. The final and general comments made by the Reviewer very well summarize the take-home message of the entire work, which ultimately demonstrates that a significant proportion of luminal and ER^+ tumors are likely to benefit from retinoid-based therapeutic strategies. For this reason, we have partially rephrased these comments and added them in the last part of the DISCUSSION (page 20, lines 13-20), as we feel that they represent and excellent conclusion for our work. With respect to this, it is worthwhile mentioning that the results obtained represent the rational for an independent clinical trial, which we will conduct in post-menopausal and for which we have already obtained the approval of the Ethical Committee. As we think that this point may be of interest to the readership of EMBO Mol Med, we have mentioned it in the very last part of the DISCUSSION (page 20, lines 17-20).

3. To balance the data relating to the cell lines, we expanded the number of primary tumors (from 30 to 45) subjected to the short-term tissue culture studies. The increase in the number of primary tumors analyzed does not alter the major result of the study, i.e. a relatively large proportion of luminal and ER^+ tumors is sensitive to the action of ATRA. In addition and as suggested also by Reviewer No. 1, we performed high-throughput gene-expression analysis on 16 samples to define the differential genomic effects exerted by ATRA in *Luminal* and *TN* or *Basal* tumors. The data are presented in Fig 13 and Suppl. Tables S5-S8. The results are described in the RESULTS section under the heading "*Transcriptional responses to ATRA in short-term cultures of mammary tumors* " from page 16, line 4 on. The results obtained are interesting in so far as they demonstrate that TN tumors are not unresponsive to the genomic effects of ATRA, in spite of their resistance to the anti-proliferative action of the retinoid. However, the type of genes whose expression is modulated by ATRA in Luminal and TN tumors is remarkably different.

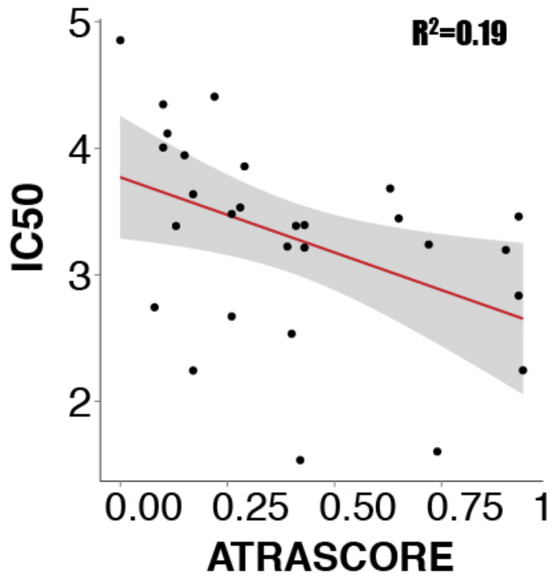

Figure *Correlation graph*

2nd Editorial Decision 20 March 2015

Thank you for the submission of your revised manuscript to EMBO Molecular Medicine. We have now received the enclosed reports from the referees that were asked to re-assess it. One Reviewer (#2) was unavailable. Since s/he had only minor issues, your revision was checked at the editorial level regarding the points s/he raised.

As you will see the reviewers are now globally supportive and I am pleased to inform you that we will be able to accept your manuscript pending the following final amendments:

1) I note that in the legend to Table 1 you state "Significant p-values for the indicated comparisons (Student's t-test) are shown in red", but I cannot see any red lettering. Also, please clarify that the P values indicated in the various figures are indeed P values by adding "P=". You might want to decrease the font size slightly. Finally, please also list the actual P values for the table section of figure 5.

2) Every published paper now includes a 'Synopsis' to further enhance discoverability. Synopses are displayed on the journal webpage and are freely accessible to all readers. They include a short standfirst as well as 2-5 one sentence bullet points that summarise the paper. Please provide the standfirst including the short list of bullet points that summarise the key NEW findings. The bullet points should be designed to be complementary to the abstract - i.e. not repeat the same text. We encourage inclusion of key acronyms and quantitative information. Please use the passive voice. Please attach this information in a separate file or send them by email, we will incorporate it accordingly.

© EMBO

Please submit your revised manuscript within two weeks. I look forward to seeing a revised form of your manuscript as soon as possible.

***** Reviewer's comments *****

Referee #1 (Remarks):

The inclusion of additional tumor samples and the global gene expression profiling strengthen manuscript. I still find it less to the point though.

Referee #3 (Comments on Novelty/Model System):

The authors have substantially revised the original manuscript be adding more convincing data. The date reported here are of significance for the possible advance of novel breast cancer treatment regiment(s) involving retinoic acid and/ or RAR selective selective retinoids.

2nd Revision - authors' response 23 March 2015

We made the minor modifications required under point 1 to various figures with particular reference to Fig. 5. Hoping that the manuscript may now be acceptable for publication in EMBO Mol Med, we thank you for Your help and look forward to receiving Your final decision on our manuscript.

© EMBO
